# A TDDFT investigation of the Photosystem II reaction center: Insights into the precursors to charge separation

**DOI:** 10.1073/pnas.1922158117

**Published:** 2020-08-03

**Authors:** Maeve A. Kavanagh, Joshua K. G. Karlsson, Jonathan D. Colburn, Laura M. C. Barter, Ian R. Gould

**Affiliations:** ^a^Department of Chemistry, Molecular Sciences Research Hub, Imperial College London, W12 0BZ London, United Kingdom;; ^b^Institute of Chemical Biology, Molecular Sciences Research Hub, Imperial College London, W12 0BZ London, United Kingdom;; ^c^Molecular Photonics Laboratory, School of Natural and Environmental Sciences, Newcastle University, Newcastle Upon Tyne NE1 7RU, United Kingdom;; ^d^School of Chemistry, University of St. Andrews, St. Andrews KY16 9ST, Scotland

**Keywords:** photosynthesis, TDDFT, Photosystem II, charge-separation precursors, structure–function relationship

## Abstract

Photosystem II (PS II) captures solar energy and directs charge separation (CS) across the thylakoid membrane during photosynthesis. The highly oxidizing, charge-separated state generated within its reaction center (RC) drives water oxidation. Spectroscopic studies on PS II RCs are difficult to interpret due to large spectral congestion, necessitating modeling to elucidate key spectral features. Herein, we present results from time-dependent density functional theory (TDDFT) calculations on the largest PS II RC model reported to date. This model explicitly includes six RC chromophores and both the chlorin phytol chains and the amino acid residues <6 Å from the pigments’ porphyrin ring centers. Comparing our wild-type model results with calculations on mutant D1-His-198-Ala and D2-His-197-Ala RCs, our simulated absorption-difference spectra reproduce experimentally observed shifts in known chlorophyll absorption bands, demonstrating the predictive capabilities of this model. We find that inclusion of both nearby residues and phytol chains is necessary to reproduce this behavior. Our calculations provide a unique opportunity to observe the molecular orbitals that contribute to the excited states that are precursors to CS. Strikingly, we observe two high oscillator strength, low-lying states, in which molecular orbitals are delocalized over Chl_D1_ and Phe_D1_ as well as one weaker oscillator strength state with molecular orbitals delocalized over the P chlorophylls. Both these configurations are a match for previously identified exciton–charge transfer states (Chl_D1_^+^Phe_D1_^−^)* and (P_D2_^+^P_D1_^−^)*. Our results demonstrate the power of TDDFT as a tool, for studies of natural photosynthesis, or indeed future studies of artificial photosynthetic complexes.

## Significance

Examining the excited states of the Photosystem II reaction center furthers our understanding of available charge separation pathways that lead to successful photosynthesis. Our results comprise the largest complete model of the Photosystem II reaction center to be described using time-dependent density functional theory reported in the literature to date. We reveal the molecular orbitals contributing to the excited states that are precursors to charge separation. We demonstrate that our model can successfully predict the action of specific mutations, a valuable tool for the agricultural industry. These models may also be beneficial in informing the design of artificial photosynthetic complexes as well as enhanced bioengineered photosystems.

During oxygenic photosynthesis, a photon of sunlight may be absorbed by any of the >250 chlorophylls or carotenoids in the Photosystem II (PS II) complex (1). This energy is then rapidly transferred to the reaction center (RC), where a remarkable charge separation (CS) process takes place, resulting in a radical pair species with oxidative ability, which is unparalleled in nature (+1.1 eV) (2). The RC pigments are bound by the protein chains D1 and D2, themselves being arranged in two nearly symmetrical branches (3). Each branch hosts a central chlorophyll *a* (Chl) molecule (P_D1_ and P_D2_, respectively), a second Chl molecule (Chl_D1_ and Chl_D2_), and a pheophytin (Phe) molecule (Phe_D1_, Phe_D2_), which together comprise the six core pigments of the PSII RC. Beyond these, a plastoquinone (Q_A_ and Q_B_) and a further peripheral Chl (Chlz_D1_ and Chlz_D2_) are found on each chain.

**Scheme 1.**
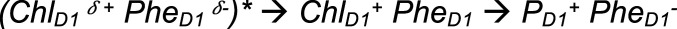


**Scheme 2.**
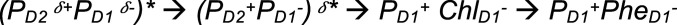


CS proceeds from an initial delocalized excited state (ES) (2), identified as a pair of exciton–charge transfer states (Chl_D1_^δ+^Phe_D1_^δ−^)* and (P_D2_^δ+^P_D1_^δ−^)* (4), down one of two charge-transfer (CT) pathways (5–7) (Schemes 1 and 2), producing radical pair species P_D1_^+^Phe_D1_^−^.

One of the two CT pathways may be favored due to the specific conformation of the protein (7) or the wavelength of incident light (8). Vibronic coupling between states has been identified (9), which facilitates switching between the two available pathways (10) to further assist efficient CS.

Histidine (His) residues ligate the two P chlorophylls in the wild-type (W-T) crystal structure (3). These ligands have high polarizability and will stabilize the positive charge on the P chlorophylls during the formation of the primary radical pair state P^+^Phe_D1_^−^ (11). The relative perturbation of the W-T absorption spectrum following mutation near the P_D1_ and P_D2_ sites, respectively, suggests that the cationic charge in the radical pair is in fact shared by the two P chlorophylls, with around 80% localized on P_D1_ and 20% on the P_D2_ (11–14). In particular, replacement of these His residues in site-directed mutagenesis studies (11, 15, 16) has allowed the spectral features of the P chlorophylls’ absorption to be identified, with P_D1_ absorption peaks at 672.5 nm (at 80 K) and 433 nm (at 298 K) and P_D2_ at 436 nm (at 298 K) (11).

The powerfully oxidizing cation of the P^+^ Phe_D1_^−^ radical pair proceeds to abstract an electron from substrate water, which is bound at the oxygen-evolving complex via a tyrosine residue, Tyr_z_ (17). After four successive excitations and electron-transfer cycles, two water molecules are oxidized to form one oxygen molecule as a byproduct (18, 19). Meanwhile, Phe_D1_^−^ transfers an electron to Q_A_, which in turn reduces the loosely bound Q_B_. After a second successive excitation and electron-transfer cycle, Q_B_^−^ is further reduced to Q_B_^2−^ and becomes protonated to plastoquinol. Plastoquinol then leaves the binding site to carry its reducing equivalents over to Photosystem I and is replaced by a new plastoquinone (20).

## Site-Directed Mutagenesis

Due to the considerable congestion of the PS II RC absorption spectrum, identifying intermediate states in the CT process poses a difficult challenge (1). Site-directed mutagenesis has therefore been employed to pinpoint the cofactors that are implicated in these intermediates (4, 11, 15, 16, 21–24).

A number of investigations have selected the ligands of P_D1_ and P_D2_ (residues D1-198 and D1-197) as the sites for mutation. These sites each host His residues in the W-T and examples can be found in the literature of their mutation to alanine (Ala), asparagine (Asn), leucine, serine (Ser), glutamine (Gln), and glutamate (Glu) (4, 11, 15, 16, 23, 24). In the case of mutation to smaller amino acids Ala and Asn, there is likely to be a water molecule coordinating the P chlorophyll Mg atom (11, 13, 15, 16, 23) in place of the nitrogen on the His. In this work, we have chosen to model two mutants, D1-His-198-Ala (H198A) and D2-His-197-Ala (H197A), each with water molecules inserted to coordinate the Mg atoms.

Evidence from experimental studies of the His-198-Ala mutant (replacement of the P_D1_ His ligand with an Ala) show that this mutation increases the stability of the P^+^ cation (11, 15, 16) possibly by stabilization of charge onto the new water ligand to the P_D1_ chlorophyll (13). The mutation is observed to increase the quantum yield of the primary radical pair state P^+^Phe_D1_^−^, which is due to a lower free energy of the primary radical pair (15). Also observed for this mutant is a lower fluorescence yield (15) and a greatly increased rate of recombination of the radical pair state relative to the W-T (11). Despite increasing radical pair yield, overall, the photosynthetic activity for this mutant would be expected to be lower than for the W-T, as the driving force for secondary CT step Y_Z_P^+^ → Y_Z_^+^P is diminished. This is evidenced by the oxygen evolution rate for His-198-Ala mutant being 50 to 70% that observed for the W-T (11).

In the case of the analogous D2 branch mutant His-197-Ala, a slightly increased rate of recombination of the radical pair suggests that the P^+^ cation has been stabilized by the mutation but to a lesser extent than the D1 branch mutation (11). This is explained by the mutation stabilizing the P_D2_ chlorophyll to cation localization and the positive charge of the resultant P^+^ cation being more evenly shared by P_D1_ and P_D2_.

Explicit quantum mechanical (QM) modeling of these mutant RCs allows us to closely examine the effect of the mutation on the molecular orbitals, something that has not been achievable before now.

## Models of the RC

Complementing the vast body of experimental work on the RC are various computational studies of the system (2, 5, 6, 10, 13, 14, 25–35). Early theoretical studies of the PSII RC revealed that there must be some key structural differences between this and the purple bacterial RC. This was proven first by the Multimer model (2), which considered weak excitonic interactions between the majority of RC cofactors and could produce a good approximation of the experimental absorption spectrum despite there being no crystal structure data available at the time. As improvements in the resolution of crystal structures of PSII were published (3, 36, 37), it enabled new models to be built, based upon these parameters (13, 26–28, 32, 33). These new models incorporated the coupling between pigments (32) and/or the atomic positions of the pigments themselves (13, 26, 33) as determined from the crystal structure. Among the latter, the size of the model became restrictive, and care had to be taken in choosing how much of the protein environment could be included and at what level of theory (26–28). Several authors opted for an idealized model to reduce computational cost (26, 28); however, in one example, it was shown that inclusion of the phytol chains in single residue QM calculations emphasized the preference for the active branch CT pathway over the inactive (33). Additionally, there is some debate in the literature as to whether geometry optimization (13, 28, 33), or relaxation using molecular dynamics (27, 34, 38), of the crystal structure atomic positions is necessary to produce an accurate model of the PS II RC or whether the crystal structure geometries are sufficient (25, 26). A number of alternative mathematical models have also been designed where model parameters are based upon fits to experimental spectroscopic data (5, 10, 29, 35, 39).

We believe the RC lends itself best to treatment as a supermolecule in order to accurately describe its ESs, which are likely to be delocalized across multiple pigments due to the weak coupling between all core RC pigments. So far, this has been a difficult computational task, due to the high computational demand associated with a quantum system of this size. The Multimer model (2, 32) and its relatives (5, 10, 29, 35, 39), for example, use simple dipole approximations for the chromophores (33, 35, 40) and consider only local excitations of the chromophores (2), a single CT state (5), or only active branch chromophores (10, 29). Other QM models have broken up the QM region into constituent chromophores (14, 33, 38, 41, 42) and/or used hybrid methods to mitigate the large computational costs (13, 14, 28, 38). The six RC chromophores (without phytol chains) were included in a supermolecular configuration interaction singles calculation in 2011 (26). Previously, the largest (∼300 atoms) time-dependent density functional theory (TDDFT) model of the RC includes six chromophores and two quinones reduced to their rings only (no side chains or phytol chains included in the QM region) (28).

Our most complete QM model, reported here, comprises 6 chromophores (including side chains and full phytol chains) along with 23 whole residues and 2 water molecules, which have been explicitly included (1,299 atoms), in keeping with recent insight (43–45). Therefore, the present work constitutes the largest complete ES QM model of the PSII RC reported to date, using the robust method of TDDFT.

TDDFT is based on the ground state method of density functional theory, which uses charge density as an analog to the electronic wavefunction. TDDFT provides a method to solve the many body time dependent Schrödinger equation as a way to calculate excitation energies and oscillator strengths (46).

Long-range hybrid-corrected functional wB97x-D was employed in this work as this has been shown to be effective for diffuse (delocalized) and CT-like ESs (47, 48).

## Results

### Four W-T Models.

Four W-T models were made, labeled henceforth as models 1 to 4 (details in Table 1), all including the six core chlorins (P_D1_, P_D2_, Chl_D1_, Chl_D2_, Phe_D1_, and Phe_D2_) and four chlorophyll ligands: His-198; His-197 coordinating P_D1_ and P_D2_, respectively; and two water molecules coordinating the Chl_D1_ and Chl_D2_ chlorophylls.

**Table 1.** Excited state energies, oscillator strengths, and dominant transitions for states 1 to 6 for W-T models 1 to 4

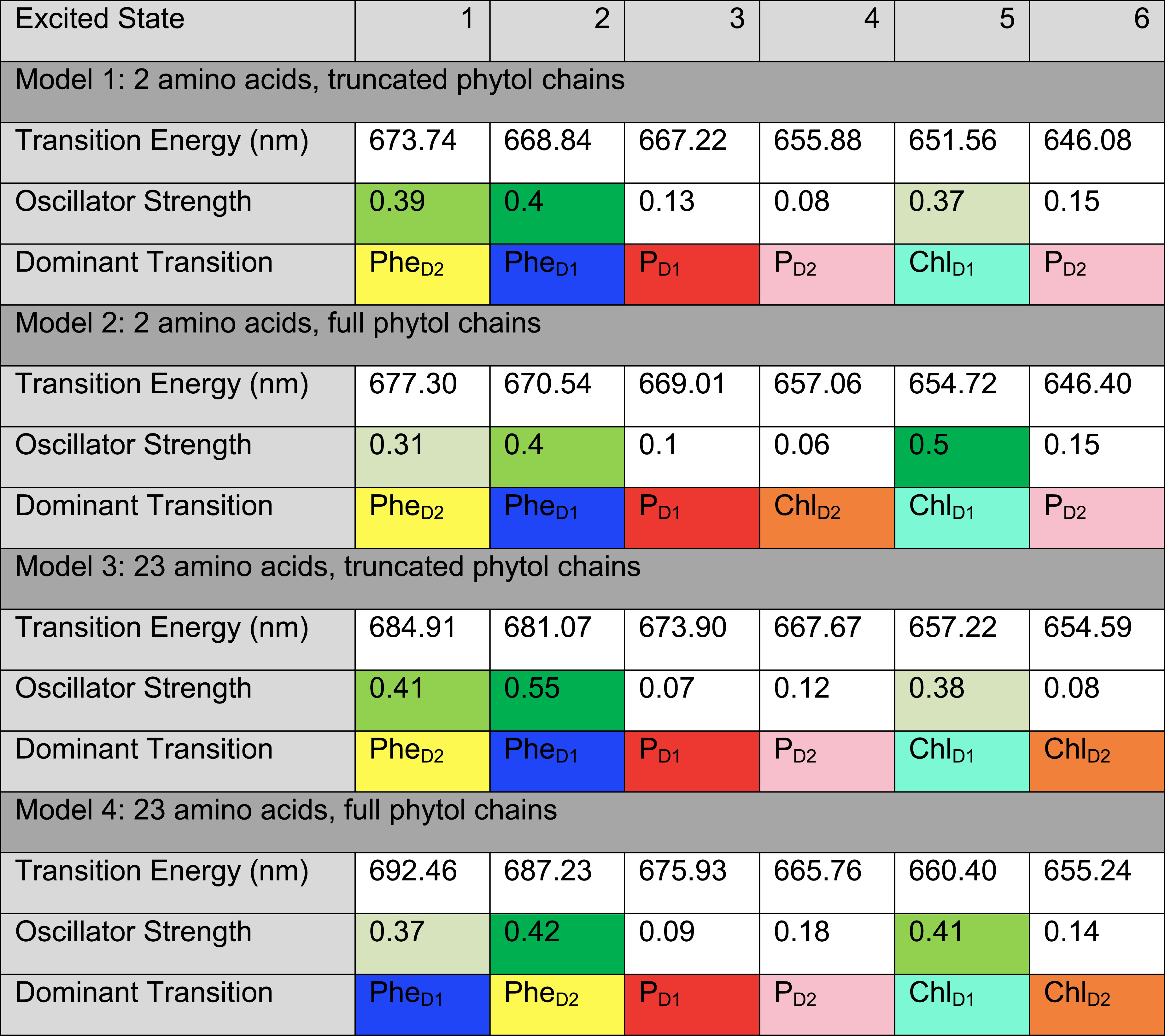

Results are as calculated using wB97x-D/6-31G (d, p). Bright oscillator strength states are colored green with darkest green indicating the highest oscillator strength state. Dominant transitions are also colored by cofactor as in Figs. 2*A* and 4: Phe_D1_, blue; Chl_D1_, aquamarine; P_D1_, red, P_D2_, pink; Chl_D2_, orange; and Phe_D2_, yellow. States 7 to 22 for all models were also calculated; these results are discussed further in subsection Higher Energy ESs and full results are presented in *SI Appendix*, Tables S1*–*S4 *A*–*C*.

**Fig. 1.**
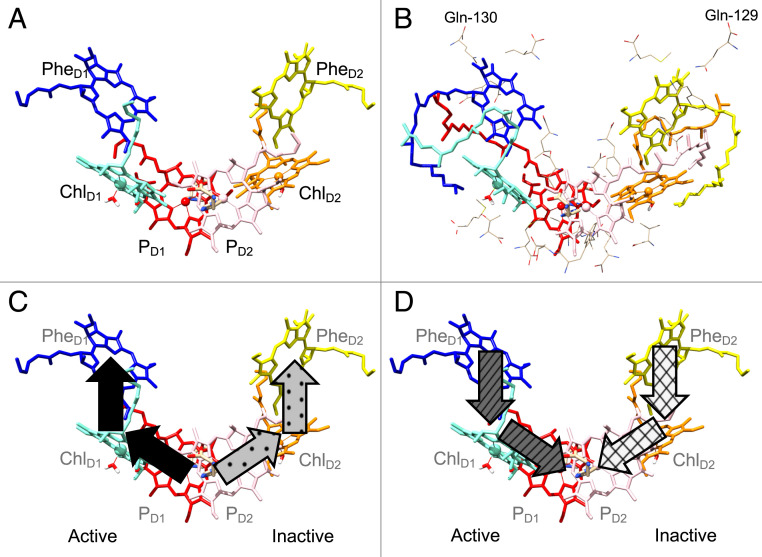
(*A*) Model 1 structure including the six chlorins with phytol chains truncated up to the methyl group on C7, two waters coordinating the Chl_D1_ and Chl_D2_, and two His residues coordinating the P chlorophylls. Cofactors are colored as in Table 1 and Figs. 2*A* and 4: Phe_D1_, blue; Chl_D1_, aquamarine; P_D1_, red; P_D2_, pink; Chl_D2_, orange; and Phe_D2_, yellow. (*B*) Model 4 including the six cofactors with full phytol chains, two water molecules, and 23 amino acid residues (wire structure). All residues lie within 6 Å of the porphyrin ring-centers except Gln-130 and Gln-129 (labeled). (*C*) Black arrows show CT up the active branch (from P chlorophylls toward Chl_D1_ or Phe_D1_ or from Chl_D1_ toward Phe_D1_), dotted arrows show CT up the inactive branch (from P chlorophylls toward Chl_D2_ or Phe_D2_ or from Chl_D2_ toward Phe_D2_). (*D*) Striped arrows show CT down the active branch (from Phe_D1_ chlorophylls toward Chl_D1_ or P chlorophylls or from Chl_D1_ toward P chlorophylls), and hashed arrows show CT down the inactive branch (from Phe_D2_ chlorophylls toward Chl_D2_ or P chlorophylls or from Chl_D2_ toward P chlorophylls).

Models 1 and 3 had the phytol chains of the six chlorins truncated up to the methyl group on C7, and for model 2 and 4, they were left as full-length chains (see structures of model 1 and 4 in Fig. 1 *A* and *B*). Models 3 and 4 included a further 21 whole, neutral amino acid residues (terminated COOH/NH_2_), including the nearest residues to the centers of the six porphyrin rings (within 6 Å radius), in addition to Gln-130 (D1 branch) and Gln-129 (D2 branch) (Fig. 1*B*). For a list of residues included please, see *SI Appendix*, Table S0.

ESs are characterized by a weighted combination of transitions, represented by pairs of occupied and virtual molecular orbitals (MOs), where the weights are given by the computed configuration-interaction coefficients. Each ES can be labeled as an excitation of the cofactor, which contributes most highly to the occupied MOs involved in the major transitions (highest configuration-interaction coefficient) of that ES. The contribution of each cofactor to an MO was determined as the sum of atomic contributions to that MO over all atoms in that cofactor (49).

The six lowest-energy ESs of the four W-T models, as calculated using TDDFT with the wB97x-D (47, 48) functional and 6-31G (d, p) (50–56) basis set, are described here. The major transitions and oscillator strengths for states 1 to 6 are presented in Table 1.

For all W-T models, the brightest states, the first, second, and fifth, correspond to the Phe excitations and the Chl_D1_ excitation. From Table 1, we note that a higher oscillator strength is calculated for state 5 (Chl_D1_ excitation) for the full phytol chain models 2 and 4 in comparison for models 1 and 3. Whereas adding amino acids to the models results in higher oscillator strengths for states 1 and 2 (Phe excitations): without the phytol chains (models 1 and 3), we see an increase of oscillator strengths for states 1 and 2 from 0.39 and 0.4 to 0.41 and 0.55 on adding the amino acids. Similarly, with the phytol chains (model 2 and 4), states 1 and 2 increase in oscillator strength from 0.31 to 0.37 and from 0.4 to 0.42, respectively (Table 1).

When comparing models 3 and 4, it can be seen that the energies of the Phe excitations are shifted by addition of phytol chains when the 23 amino acids are present, including the phytol chains raises the energy of Phe_D2_ excitation above Phe_D1_ (Table 1). When including the surrounding amino acids in models 3 and 4, the Chl_D2_ excitation appears highest in energy, whereas for the two models without the amino acids, the Chl_D2_ excitation is lower in energy (model 2) or does not dominate any of these states (model 1).

**Fig. 2.**
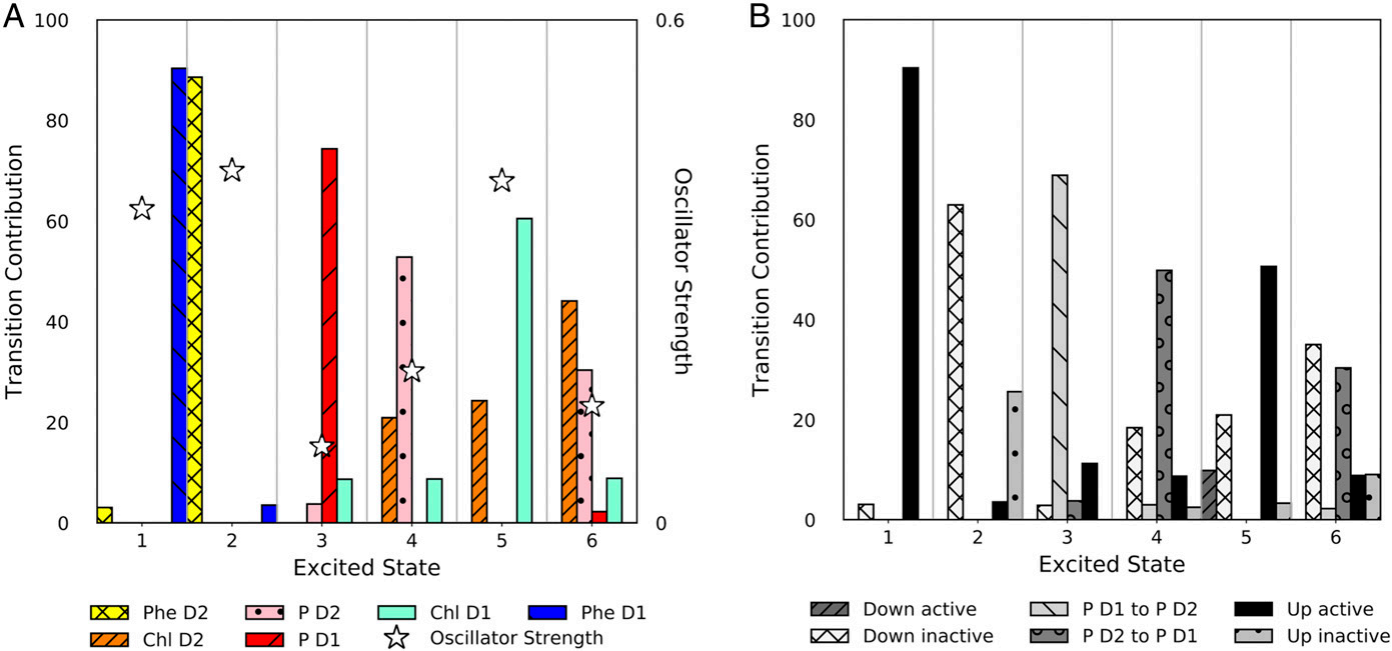
(*A*) Contribution from occupied orbitals on each cofactor for transitions contributing to each state for W-T model 4—with 23 amino acids and full phytol chains. Oscillator strength for each state is plotted on secondary *x* axis in white stars. (*B*) CT character of transitions contributing to each state for W-T model 4—with 23 amino acids and full phytol chains. Direction of CT is shaded as in Fig. 1 *C* and *D*. (States 7 to 22 for model 4 were also calculated; these results are discussed further under the subheading Higher Energy ESs and full results for all models are presented in *SI Appendix*, Tables S1–S6 *A* and *B*.)

**Fig. 3.**
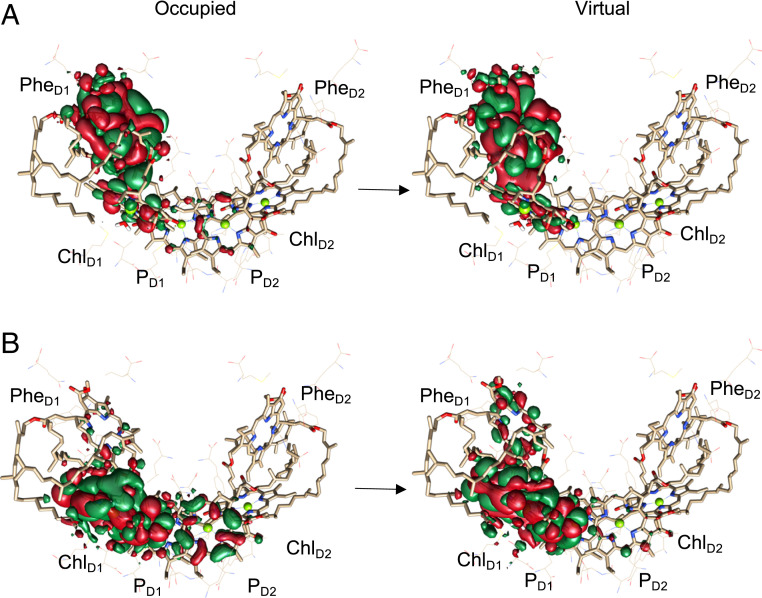
The molecular orbitals, states obtained from model 4 (23 amino acids and full phytol chains), participating in the most dominant transitions for the two brightest ESs in the Qy region, pictured with an iso surface value of ±0.002. (*A*) The transition contributing 64.9% to state 1 for model 4, involving Phe_D1_ excitation with delocalization across Chl_D1_ and P chlorophylls. (*B*) The transition contributing 50.6% to state 5 for model 4 involving Chl_D1_ excitation with delocalization across P chlorophylls and Phe_D1_.

Some states display a high degree of mixing, which is omitted for clarity in Table 1, and a complete picture for model 4 is presented in Fig. 2. The MOs for the transitions with the highest contribution to states 1 and 5 of W-T model 4 are presented in Fig. 3.

Individual transitions, such as those pictured in Fig. 3, were analyzed for the extent of CT between cofactors using MultiWfn (49) by examining the cofactor contributions to the orbitals. For example, consider a transition where the occupied and virtual MOs are delocalized over Chl_D1_ and Phe_D1_ on the active branch. If the Phe_D1_ contribution to the virtual MO is higher than the Phe_D1_ contribution to the occupied MO, we would identify this transition to show a degree of CT character, where electron density is directed up the active branch from Chl_D1_ to Phe_D1_ during the course of the transition (as in Fig. 1*C*). If the Chl_D1_ contribution increases from the occupied to the virtual MO, then we would identify that this transition has CT character where electron density is directed down the branch.

In the Qy region (comprising first six ESs), it was found that transitions for which CT travels up the D1 branch (from P chlorophylls to Phe_D1_) are dominant for two high oscillator-strength states (states 1 and 5) for model 4 (Fig. 2*B*). For models 1 to 3, transitions with CT up the D1 branch dominate one state only (*SI Appendix*, Figs. S1–S3*B*).

For whole ESs, which are described by a linear combination of transitions, we analyzed the extent of CT using the method described in (57). We found that the CT character of these vertical excitations was minimal at low energies (*SI Appendix*, Tables S1–S4*D*).

### Higher Energy ESs.

**Fig. 4.**
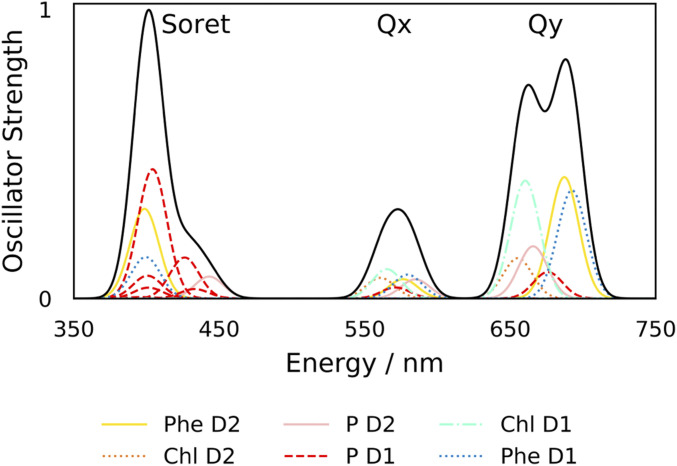
W-T Model 4 (23 amino acids, full phytol chains) calculated absorption spectrum includes three distinct bands, from lowest to highest energy, the Qy, Qx, and Soret regions. Black solid spectrum, overall calculated absorption spectrum; colored lines:, single ESs are colored according to the cofactor of the dominant transition as in Table 1 and Fig. 1: Phe_D1_, blue; Chl_D1_, aquamarine; P_D1_, red, P_D2_, pink; Chl_D2_, orange; and Phe_D2_, yellow.

For all models, higher-energy ESs were evaluated (22 ESs in total). The ES energies and oscillator strengths obtained from our models can be broadened by Gaussian functions to give a calculated absorption spectrum (Fig. 4).

We observe three distinct bands, labeled from lowest to highest energy as the Qy, Qx, and Soret bands in our calculated absorption spectrum (Fig. 4). The Qy region comprises ESs 1 to 6 and is described in detail in Table 1 and Fig. 2. The states in the Qx region mirror the Qy states, in that they correspond to an excitation of each of the six chlorins (see all results in *SI Appendix*, Tables S1–S4 *A*–*C* and Figs. S1–S4 *A* and *B* and MO diagrams for model 4 in *SI Appendix*, Fig. S4*C*). The remaining ESs, 13 to 22, form the Soret band. Six states out of the 10 in this region (443 to 396 nm) have their highest contributions from P_D1_ chlorophyll excitations (Fig. 4, Soret region; red dashed lines: states 14, 15, 17 to 19, and 22). The remaining four states have their highest contribution from P_D2_ (state 13 and 16) or Phe excitations (states 20 and 21).

### Modeling Single Residue Mutations.

**Fig. 5.**
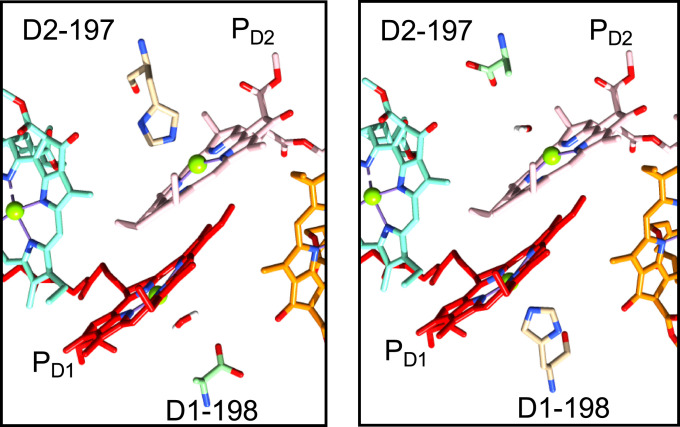
View of the P chlorophylls with either His at D1-198 (*Left*) or at D2-197 (*Right*) mutated to Ala with a water molecule inserted to ligate to the chlorophyll center for model 4 (23 amino acids, full phytol chains). Colored cofactors are as in Fig. 1; P_D1_ (red) and P_D2_ (pink) and mutated residue His to Ala (green).

Mutating the residues ligating to the P chlorophylls from His to alanine (His-198-Ala and His-197-Ala) on either the D1 or the D2 branches of the PSII RC, gave two mutant RC models (Fig. 5).

**Fig. 6.**
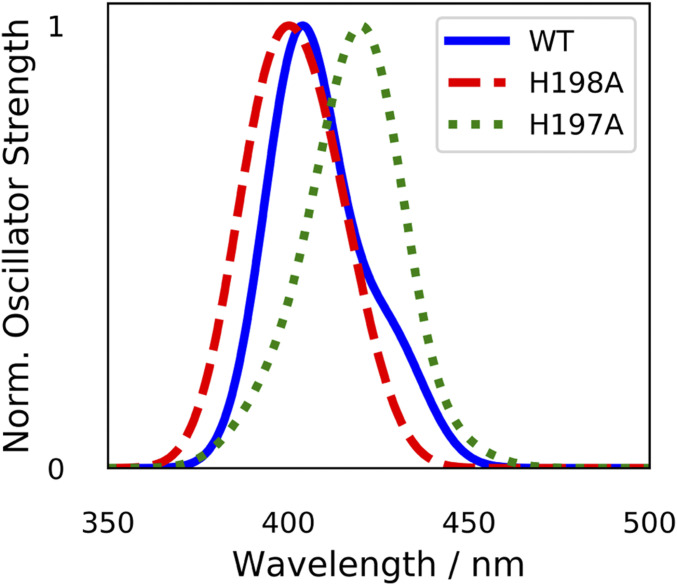
Simulated P_D1_ absorption bands in the Soret region obtained using model 4 for the W-T RC and two mutants His-198-Ala and His-197-Ala (all with 23 amino acids and full phytol chains): sum of the Gaussian-broadened ESs that are dominated by P_D1_ excitation for W-T (blue solid line), His-198-Ala (red dashed line), and His-197-Ala (green dotted line), where peak heights have been normalized to one for ease of comparison in the energy shifts. See *SI Appendix*, Figs. S1–S3*D* for model 1 to 3 results.

The high energy Soret band of the spectrum for our models 1 to 4 corresponds to a high density of P chlorophyll excitations (see W-T model 4 results in Fig. 4; also see *SI Appendix*, Tables S1–S4 *A*–*C*). When the states corresponding to P_D1_ excitations were isolated and the sum of these Gaussian-broadened states was plotted for the W-T and the two mutants it could be seen that the H198A mutation resulted in an increase in energy of the P_D1_ excitations overall, whereas the H197A mutation decreased the energy of the P_D1_ excitations overall (Fig. 6).

## Discussion

We set out to probe the ESs of the PSII RC core pigments, taking a large region (up to 1,299 atoms including H for model 4) of the crystal structure as our starting point; 22 ESs were calculated and the effects of including pigment phytol chains and surrounding protein environment in the form of whole residues was investigated.

### Active Branch Excitations Are Favored When the Local Environment Is Included.

We examined the effects of the environment on oscillator strengths of the ESs as well as the atom contributions to the occupied and virtual MOs. We find that our largest model (model 4), which includes the phytol chains and 23 amino acids, demonstrates the strongest preference for active branch excitations.

From Table 1, we see that for all our models the high oscillator-strength states are Phe and Chl_D1_ excitations. Upon inclusion of the phytol chains the Chl_D1_ (state 5) oscillator strength is increased. Investigation of each transition’s direction of CT (Fig. 2*B* and *SI Appendix*, Figs. S1–S3*B*) revealed that inclusion of both the phytol chains and the amino acids increases the contribution of transitions which direct CT up the active branch. Both state 1 and 5 are dominated by transitions of this nature for model 4 (Fig. 2*B*). Transitions that progress toward successful generation of the radical pair P_D1_^+^Phe_D1_^−^ are therefore favored when the local environment is included.

The lowest-energy ES has been assigned to Chl_D1_ excitation (27); our results obtained using model 4 are indicative of agreement as the lowest energy ES is identified as a Phe_D1_ excitation with delocalization across Chl_D1_ (Table 1 and Fig. 3). In contrast, models 1 to 3 have their lowest-energy ES attributed to Phe_D2_ on the inactive branch. Our calculations reveal the sensitivity of the site energies to cofactor configurations. We note that, while all cofactor excitations within the Qy region lie close in energy, the specific conformation of the pigments in the crystal structure (3) may lead to a higher site energy for localized Chl_D1_ excitations than the Phe_D1_ in our calculations.

### A Closer Examination of the Active Branch Excitations.

TDDFT allows us to examine the effect of initial excitation and thus the precursor states to the experimentally observed radical pair state intermediates. Two of the brightest states, obtained from model 4, are delocalized across the D1 branch cofactors (Fig. 3). This configuration is consistent with the exciton–CT ES (Chl_D1_^δ+^Phe_D1_^δ−^)* (Scheme 1), previously identified by stark spectroscopy on site-directed mutants (4). This state is the precursor to the dominant CS pathway.

Initial CS may proceed via one of two pathways (4): via either Scheme 1 or 2 in which CS originates in the P chlorophyll pair from (P_D2_^δ+^P_D1_^δ−^)*. Thus, we are unsurprised to find states involving excitation of the P chlorophylls in the darker states 3, 4, and 6, signifying the existence of another pathway (Fig. 2*A*; for MO diagrams for model 4, see *SI Appendix*, Fig. S4*C*), which may be more favorable for a different realization of the disorder of the RC (1, 7).

### Simulating the Absorption Spectrum of the RC.

The experimental low-temperature absorption spectrum of the smallest charge-separating complex that can be isolated from PSII, the D1/D2 cytochrome (Cyt) b559 PSII RC complex, has three distinct peaks in the region from 700 to 400 nm (58, 59) (Table 2). These are labeled, from low to high energy, the Qy, the Qx, and the Soret region. It should be noted that the D1/D2 Cyt b559 RC contains six Chl and two Phe and thus contains two peripheral chlorophyll molecules, which are not included in our models. These two additional chlorophyll molecules are very loosely coupled to the RC pigments and are thus commonly excluded from models of the RC (2, 6, 28).

**Table 2.** Experimental RC absorption spectrum at 10 K (from ref. 58)

Band	D1-D2-Cyt b559 complex absorption spectrum peak wavelengths (10 K)/nm	Approximate absorbance
Qy	679	0.9
	671	0.8
Qx	542	0.1
Soret	∼432	0.8
	∼416	1.0

Our calculated spectrum (Fig. 4) has three distinct bands in the same region 350 to 750 nm; states 1 to 6 make up the high oscillator-strength Qy band and are discussed in detail above; states 7 to 12 all have relatively low oscillator strength and fall within the Qx region; and states 13 to 22 comprise the Soret band (see *SI Appendix*, Fig. S4 *A* and *B* and Table S4 *A*–*C* for the nature of these ESs). As can be seen in Fig. 4, our model is able to reproduce the main features of the D1-D2-Cyt b559 complex low-temperature ultraviolet-visible absorption spectrum (58).

Diner et al. have shown that P^+^ − P absorption-difference spectroscopy on W-T RCs shows a bleach in the Soret region at 433 nm, indicating that the chlorophyll bearing the cation (P_D1_ and, to a lesser extent P_D2_) is absorbing in this region (11). Our model finds that eight states out of the ten in the region of 443 to 396 nm have their highest contributions from P chlorophyll excitations. Of these, six states are dominated by P_D1_ excitations. Gaussian broadened peaks for states in the Soret region have been summed to give the blue spectral line in Fig. 6.

### Mutant Reaction Centre Models.

With our chosen method of TDDFT we were able to investigate the effect on the ground state absorption of the cofactors following mutation of the residues ligating to the P chlorophylls from His to alanine (His-198-Ala and His-197-Ala). The energies and oscillator strengths of the states attributed to P_D1_ excitation were broadened using Gaussian functions and their sum plotted for model 4 W-T, His-198-Ala and His-197-Ala. The wavelengths of the center of the absorption peaks of these simulated P_D1_ bands were determined from the spectra obtained using model 4 for the W-T and mutant RCs (Fig. 6). It can be seen that the D1 branch mutation, His-198-Ala, has shifted the band to higher energies whereas the D2 branch mutant has had the opposite effect.

Experimental studies on these mutants have been previously reported by Diner et al. and have shown that the prominent bleaching band in the P^+^ − P absorption-difference spectrum for the W-T RC is blue-shifted from 433 nm to ∼431 nm upon mutation of D1 branch mutant His-198 to Ala, whereas for the D2 branch His-197-Ala mutant this band is red-shifted to ∼434 nm (11).

The results from our calculations, using Model 4, therefore show agreement with the observed shifts in the experimental P^+^ − P absorption band bleaching frequency energies for both of these mutants (Fig. 6), thus demonstrating the predictive capabilities of our model. Interestingly we did not see such an agreement for smaller models 1 to 3 (*SI Appendix*, Fig. S1–S3*D*), which emphasizes further the importance of including the phytol chains and amino acids in future models of the PSII RC.

In conclusion, we present the results of TDDFT calculations on the largest QM model of the PSII RC reported in the literature to date. Four models of different sizes were built and their ESs examined with TDDFT. The molecular orbitals contributing to two out of the three highest oscillator strength states for our largest model (model 4—full phytol chains and 23 amino acids) are delocalized over the D1 branch cofactors and with transitions having CT character directed up the active branch (Fig. 2*B*), which is in keeping with known precursor states to CS (4, 7). We observe two different states with this character, one localized on Chl_D1_ and the other on Phe_D1_.

Strikingly, the main features of the experimentally observed low-temperature absorption spectrum of isolated D1 D2 Cyt b559 (containing six Chl and two Phe) are reproduced by all of our models (Fig. 4 and *SI Appendix*, Figs. S1–S3*C*). Furthermore, models of the two mutant RCs, with phytol chains and amino acids included, are able to produce the expected shifts in the P_D1_ bands in the room temperature absorption spectrum (Fig. 6) compared with W-T, further validating our largest model and demonstrating deeply promising predictive capabilities of using TDDFT.

## Materials and Methods

### Methods.

#### Model preparation.

In total, 12 different model structures have been prepared, based upon the 1.9-Å resolution crystal structure of PS II from *Thermosynechococcus vulcanus* (3). The RC residues of interest were identified using Chimera (60) and hydrogens were added in GaussView (61). Prior to calculation, the hydrogen positions for each model were optimized using Austin model 1 (AM1) semiempirical theory in Gaussian (62), with heavy atom positions frozen.

For all model structures, the six-core RC chlorins (P_D1_, P_D2_, Chl_D1_, Chl_D2_, Phe_D1_, and Phe_D2_) were included, as were two water molecules coordinating the Chl_D1_ and Chl_D2_ chlorophylls. For models 1 and 3, the phytol chains of the chlorins were truncated up to the methyl group on C7, and for models 2 and 4, they were left as full-length chains (Fig. 1 *A* and *B*). For all models, the His ligands (or mutated residues in their place) coordinating the two P chlorophylls, residues D1-198 and D2-197, were included. For half of all models, a further 21 amino acids were included to simulate the surrounding protein environment; 19 of these were chosen as the nearest whole residues to the centers of the 6 chlorin porphyrin rings that lay within 6 Å of the chlorophyll Mg atoms or Phe nitrogen atoms in the crystal structure. In addition, two residues Gln-130 (8 Å away from Phe_D1_ ring) and Gln-129 (7 Å away from Phe_D2_ ring) were included in the larger models as these are known to affect radical pair formation having been targets for site-directed mutations (22). For a list of residues included please, see *SI Appendix*, Table S1. All amino acid residues were neutrally charged for ease of computation with TDDFT, residues were terminated -NH_2_/-COOH, and the COOH dihedral angles were set to 0° prior to hydrogen optimization.

Details of the W-T model structures used are presented in Table 1, and the structure of two models (models 1 and 4) can be seen in Fig. 1.

In addition to the W-T models detailed above, each of the four model structures in Table 1 were reproduced as two mutants, H198A and H197A. A mutation from D1-198/D2-197-His to Ala was created by replacing the imidazole side chain with a hydrogen. A water molecule was inserted in place of the His nitrogen to ligate the P chlorophyll (see Fig. 5 for the position of these ligands in mutant models 4: H198A and H197A; similar figures are included in *SI Appendix*, Figs. S1–S3*E* for other models). The atomic positions of the new methyl side chain and water molecule were optimized using AM1 in Gaussian (62) while holding fixed all other atoms in the system.

#### Computational methods.

TDDFT was used to map the 22 lowest energy ESs of each model. ES calculations were carried out with wB97X-D (47, 48)/6-31G (d, p) (50) using Gaussian (62). Model 1 calculations were repeated with a diffuse function using 6-31+G (d, p) (50–56) (for results, see *SI Appendix*, Table S7 *A*–*C*). Models 1 and 4 calculations were repeated with CAM-b3LYP (63) for comparative purposes (for results, see *SI Appendix*, Tables S8 and S9 *A*–*C*).

For each molecular orbital appearing in the TDDFT output, the percentage contribution of each atom in the model to the orbital was analyzed with MultiWfn (49) using Mulliken population analysis (64). The atom contributions were tabulated and the sum of all atom contributions over all of the atoms in each cofactor was calculated for the six cofactors. As such, each orbital can be described by its percentage contributions from each cofactor (*SI Appendix*, Tables S1–S4*C*). To produce the molecular orbital images in this work, the Cubegen function in Gaussian (62) was used to generate molecular orbital surfaces; these were then viewed using Chimera (60), with the chosen isovalue of +0.002/−0.002 (*SI Appendix*, Fig. S4*C*).

The extent of CT (57) was evaluated for each state calculated for all models in Gaussian (62) using keyword “pop = DCT.” The identity (element, cofactor) of the nearest atom to the center of density depletion and increment from ground state to excited state are presented in *SI Appendix*, Tables S1–S4*D*. A state was considered CT if the nearest atoms to the tail and head of the vector representing CT extent were located on different cofactors.

## Supplementary Material

Supplementary File

## Data Availability

All data with respect of Gaussian output files (.log and .fchk) are freely available at the Zenodo repository (DOI: 10.5281/zenodo.3581084).
