# Supplementary material for: A TDDFT investigation of the Photosystem II reaction center: Insights into the precursors to charge separation
Source: Proc Natl Acad Sci U S A. 2020 Aug 3;117(33):19705–12. doi: 10.1073/pnas.1922158117 (PMC7443915; doi:10.1073/pnas.1922158117)
Supplement: Supplementary File [file pnas.1922158117.sapp.pdf]

Supplementary Information

Table S0 : Residues in 23 amino acid models

D1 branch	D2 branch
D1-Gln-130	D2-Gln-129
D1-Tyr-147	D2-Phe-146
D1-Pro-150	D2-Pro-149
D1-Met-172	D2-Ile-178
D1-Thr-179	D2-Leu-182
D1-Met-183	D2-Trp-191
D1-Ile-192	D2-Thr-192
D1-Leu-193	D2-His-197
D1-His-198	D2-Val-201
D1-Val-202	D2-Leu-205
D1-Phe-206	D2-Ile-213
D1-Met-214	

TD-DFT Results

Model 1 : wB97x-D/6-31Gdp (2 amino acids, truncated phytol chains)
Table S1 a) State Energies and Oscillator Strengths for Model 1 (2 amino acids, truncated phytol chains) wb97xd/6-31G(d,p)

State	Energy / nm	Oscillator Strength
1	673.74	0.3895
2	668.9	0.401
3	667.17	0.1259
4	655.77	0.0792
5	651.55	0.3688
6	645.97	0.1525
7	570.99	0.0603
8	566.15	0.0663
9	563.35	0.05
10	559.28	0.0229
11	556.25	0.1384
12	555.23	0.0623
13	442.34	0.1095
14	429.72	0.0071
15	422.06	0.0047
16	417.87	0.0828
17	406.76	0.001
18	402.78	0.0062
19	401.13	0.3973

20	397.18	0.1957
21	396.64	0.064
22	393.73	0.2271

Table S1 b) Transitions and Transition Contributions for Model 1 (2 amino acids, truncated phytol chains) wb97xd/6-31G(d,p)

State	Occupied MO	Virtual MO	Transition Contribution	Transition %
1	1141	1147	0.10657	2.3
1	1142	1148	0.49368	48.7
1	1142	1154	-0.17153	5.9
1	1144	1149	0.16544	5.5
1	1144	1150	-0.11654	2.7
2	1134	1147	0.15317	4.7
2	1134	1152	-0.12707	3.2
2	1135	1147	-0.17832	6.4
2	1135	1152	0.2022	8.2
2	1138	1145	-0.1739	6.0
2	1141	1145	-0.17059	5.8
2	1141	1147	0.49306	48.6
2	1141	1152	0.14353	4.1
3	1136	1148	0.11013	2.4
3	1137	1149	0.10782	2.3
3	1138	1145	0.17334	6.0
3	1140	1155	-0.12721	3.2
3	1140	1156	-0.10088	2.0
3	1141	1147	0.11135	2.5
3	1142	1148	-0.19145	7.3
3	1144	1149	0.4361	38.0
3	1144	1150	-0.31128	19.4
4	1133	1151	-0.14276	4.1
4	1138	1145	-0.14795	4.4
4	1139	1146	0.35964	25.9
4	1140	1150	-0.14091	4.0
4	1141	1147	-0.11766	2.8
4	1143	1149	0.24199	11.7
4	1143	1150	0.34324	23.6
4	1144	1149	0.10877	2.4
5	1133	1151	-0.11529	2.7
5	1134	1145	-0.10536	2.2
5	1134	1153	0.18087	6.5
5	1135	1153	0.10756	2.3
5	1137	1145	0.19409	7.5

5	1138	1145	0.44123	38.9
5	1138	1147	0.12184	3.0
5	1139	1146	0.2771	15.4
5	1141	1147	0.12731	3.2
5	1144	1149	-0.13285	3.5
6	1133	1146	-0.10254	2.1
6	1133	1151	-0.17219	5.9
6	1137	1156	0.10847	2.4
6	1138	1145	-0.14209	4.0
6	1138	1146	-0.1009	2.0
6	1139	1146	0.38292	29.3
6	1140	1150	0.13881	3.9
6	1140	1155	-0.10482	2.2
6	1143	1149	-0.2525	12.8
6	1143	1150	-0.32237	20.8
7	1137	1149	0.22271	9.9
7	1137	1150	0.2886	16.7
7	1138	1149	-0.10095	2.0
7	1138	1150	-0.13553	3.7
7	1140	1149	0.22716	10.3
7	1140	1150	0.28547	16.3
7	1143	1150	0.17927	6.4
7	1143	1155	-0.1845	6.8
7	1143	1156	0.19323	7.5
7	1144	1149	-0.15044	4.5
7	1144	1150	-0.20379	8.3
8	1136	1148	0.50021	50.0
8	1136	1154	-0.11455	2.6
8	1142	1148	0.3449	23.8
8	1142	1154	0.28341	16.1
9	1137	1149	-0.23288	10.8
9	1137	1150	0.20218	8.2
9	1140	1149	0.36428	26.5
9	1140	1150	-0.22296	9.9
9	1143	1149	0.23479	11.0
9	1143	1150	-0.14567	4.2
9	1144	1149	0.10562	2.2
9	1144	1155	0.20744	8.6
9	1144	1156	0.18697	7.0
10	1134	1145	-0.17322	6.0
10	1134	1147	-0.29111	16.9
10	1135	1145	-0.28404	16.1

10	1135	1147	0.33909	23.0
10	1138	1153	0.11724	2.7
10	1141	1147	0.25395	12.9
10	1141	1152	-0.26279	13.8
11	1134	1145	0.45714	41.8
11	1135	1145	0.20882	8.7
11	1135	1147	0.29662	17.6
11	1138	1145	0.1061	2.3
11	1138	1153	-0.21983	9.7
11	1141	1147	0.12368	3.1
11	1141	1152	-0.16145	5.2
12	1133	1146	0.57899	67.0
12	1139	1146	0.16676	5.6
12	1139	1151	0.28194	15.9
12	1139	1157	0.10121	2.0
13	1140	1149	-0.22333	10.0
13	1140	1150	0.25018	12.5
13	1140	1155	-0.11633	2.7
13	1143	1149	0.41689	34.8
13	1143	1150	-0.19883	7.9
13	1143	1155	0.2353	11.1
13	1144	1150	-0.20758	8.6
14	1137	1149	0.15775	5.0
14	1137	1150	0.27882	15.5
14	1138	1150	-0.12066	2.9
14	1140	1149	-0.12979	3.4
14	1143	1150	-0.13201	3.5
14	1144	1149	0.30867	19.1
14	1144	1150	0.40694	33.1
14	1144	1156	0.14562	4.2
15	1140	1145	0.11282	2.5
15	1144	1145	0.64006	81.9
15	1144	1147	0.16943	5.7
15	1144	1153	0.10399	2.2
16	1132	1149	-0.12747	3.2
16	1137	1149	-0.18761	7.0
16	1140	1149	-0.2497	12.5
16	1140	1150	0.15991	5.1
16	1143	1150	0.13852	3.8
16	1143	1155	-0.19476	7.6
16	1143	1156	0.18536	6.9
16	1144	1145	-0.14453	4.2

16	1144	1155	0.37107	27.5
16	1144	1156	0.23691	11.2
17	1143	1146	0.61318	75.2
17	1144	1146	-0.29461	17.4
18	1103	1146	-0.39981	32.0
18	1103	1151	0.17276	6.0
18	1103	1157	-0.44548	39.7
18	1103	1163	0.11162	2.5
18	1118	1146	0.17149	5.9
18	1118	1157	0.12346	3.0
19	1133	1146	-0.19586	7.7
19	1133	1151	-0.10464	2.2
19	1133	1157	-0.10791	2.3
19	1137	1155	0.12696	3.2
19	1138	1151	-0.11106	2.5
19	1138	1153	-0.11352	2.6
19	1139	1151	0.43532	37.9
19	1139	1157	-0.11466	2.6
19	1140	1149	-0.10326	2.1
19	1140	1156	-0.17922	6.4
19	1142	1154	-0.15607	4.9
19	1143	1150	-0.14679	4.3
19	1144	1149	-0.10937	2.4
20	1132	1150	-0.13024	3.4
20	1137	1149	-0.12469	3.1
20	1137	1150	-0.13627	3.7
20	1138	1153	0.10484	2.2
20	1139	1151	0.18204	6.6
20	1140	1146	-0.10556	2.2
20	1140	1149	0.17459	6.1
20	1140	1150	0.23069	10.6
20	1140	1156	0.12152	3.0
20	1143	1146	0.17627	6.2
20	1143	1150	0.12099	2.9
20	1144	1146	0.32351	20.9
20	1144	1149	0.15977	5.1
20	1144	1150	0.14701	4.3
21	1137	1150	0.12698	3.2
21	1139	1151	-0.11851	2.8
21	1140	1146	-0.1591	5.1
21	1140	1149	-0.13869	3.8
21	1140	1150	-0.15192	4.6

21	1143	1146	0.24464	12.0
21	1144	1146	0.48617	47.3
21	1144	1150	-0.10694	2.3
22	1137	1149	-0.14299	4.1
22	1137	1150	0.27058	14.6
22	1137	1155	-0.15237	4.6
22	1138	1150	-0.11786	2.8
22	1138	1153	0.17577	6.2
22	1139	1151	0.15132	4.6
22	1140	1149	-0.13278	3.5
22	1141	1152	-0.16424	5.4
22	1142	1154	-0.17466	6.1
22	1143	1149	-0.19423	7.5
22	1143	1150	0.16262	5.3
22	1143	1156	-0.18631	6.9
22	1144	1150	-0.10611	2.3

Table S1 c) Cofactor % Contributions* to Molecular Orbitals for Model 1 (2 amino acids, truncated phytol chains) wb97xd/6-31G(d, p)

MO	Phe $_{D1}$	Phe $_{D2}$	P $_{D1}$	P $_{D2}$	Chl $_{D1}$	Chl $_{D2}$
1133	0.0	0.9	0.0	0.2	0.0	98.8
1134	28.7	0.0	0.2	0.1	70.9	0.0
1135	71.3	0.0	0.2	0.1	28.4	0.0
1136	0.0	98.5	0.0	0.1	0.0	1.3
1137	0.1	0.0	38.2	44.8	16.3	0.3
1138	0.3	0.0	5.5	9.2	79.2	5.6
1139	0.0	0.5	1.0	1.3	3.9	93.2
1140	0.2	0.0	50.2	48.7	0.2	0.1
1141	99.0	0.0	0.3	0.1	0.6	0.0
1142	0.0	99.8	0.0	0.1	0.0	0.1
1143	0.0	0.0	14.2	85.4	0.1	0.0
1144	0.0	0.0	90.3	9.3	0.1	0.0
1145	8.6	0.0	0.1	0.2	91.0	0.1
1146	0.0	2.1	0.0	0.2	0.1	97.6
1147	91.2	0.0	0.2	0.0	8.6	0.0
1148	0.0	97.7	0.0	0.1	0.0	2.0
1149	0.0	0.0	64.6	35.2	0.1	0.1
1150	0.0	0.0	34.3	65.4	0.1	0.1
1151	0.0	0.2	0.1	0.3	0.0	99.4
1152	97.3	0.0	0.2	0.0	2.4	0.0
1153	2.4	0.0	0.8	0.2	96.6	0.0
1154	0.0	99.5	0.0	0.2	0.0	0.2
1155	0.0	0.0	57.5	41.6	0.3	0.1
1156	0.0	0.0	42.5	56.5	0.5	0.2

* as determined by the Mulliken Charges method in MultiWfn

Table S1 d) Nearest atoms to centers of density depletion and increment from ground state to excited state (method as described in reference 57) for Model 1 (2 amino acids, truncated phytol chains) wb97xd/6-31G(d, p)

State	Nearest Atom to Center of Density Depletion			Nearest Atom to Center of Density Increment		
	Atom	Element	Cofactor	Atom	Element	Cofactor
1	295	C	Phe D2	317	C	Phe D2
2	206	C	Phe D1	228	C	Phe D1
3	45	N	P D1	45	N	P D1
4	52	C	P D1	52	C	P D1
5	534	H	P D2	534	H	P D2
6	94	H	P D1	94	H	P D1
7	473	N	P D2	500	N	P D2
8	297	N	Phe D2	355	H	Phe D2
9	34	N	P D1	29	Mg	P D1
10	220	C	Phe D1	223	C	Phe D1
11	270	H	Phe D1	271	H	Phe D1
12	121	N	Chl D2	148	N	Chl D2
13	468	Mg	P D2	45	N	P D1
14	29	Mg	P D1	468	Mg	P D2
15	29	Mg	P D1	412	N	Chl D1
16	488	C	P D2	53	N	P D1
17	468	Mg	P D2	148	N	Chl D2
18	155	O	Chl D2	151	C	Chl D2
19	149	C	Chl D2	120	C	Chl D2
20	45	N	P D1	94	H	P D1
21	45	N	P D1	149	C	Chl D2
22	470	C	P D2	46	C	P D1

The table above describes movement of density from the ground state to the excited state as defined by single points of density depletion (nearest atom described in columns 2-4) and increment (nearest atom described in columns 5-7). The two points define a vector which gives extent and direction of CT for each state. For ES 1-12 we find no CT states, as the centers of depletion and increment of density from ground to excited state are localised on the same cofactor.

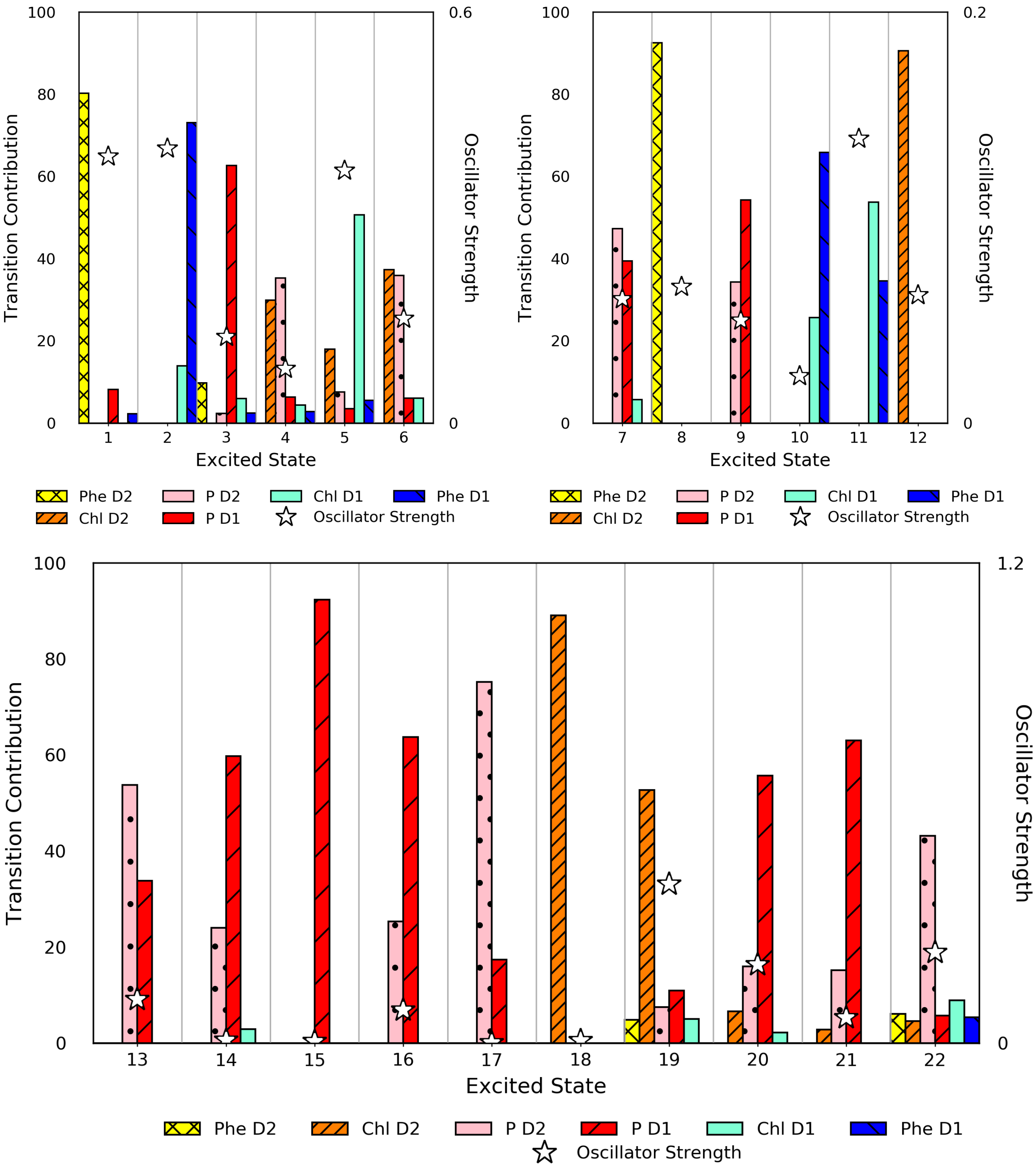

Figure S1 a) Showing the oscillator strengths (white stars) and % contribution of transitions from each cofactor excitation for W-T model 1 (2 amino acids and truncated phytol chains). Occupied histograms for model 1 (top left) states 1-6, (top right) 7-12 and (bottom) states 13-22

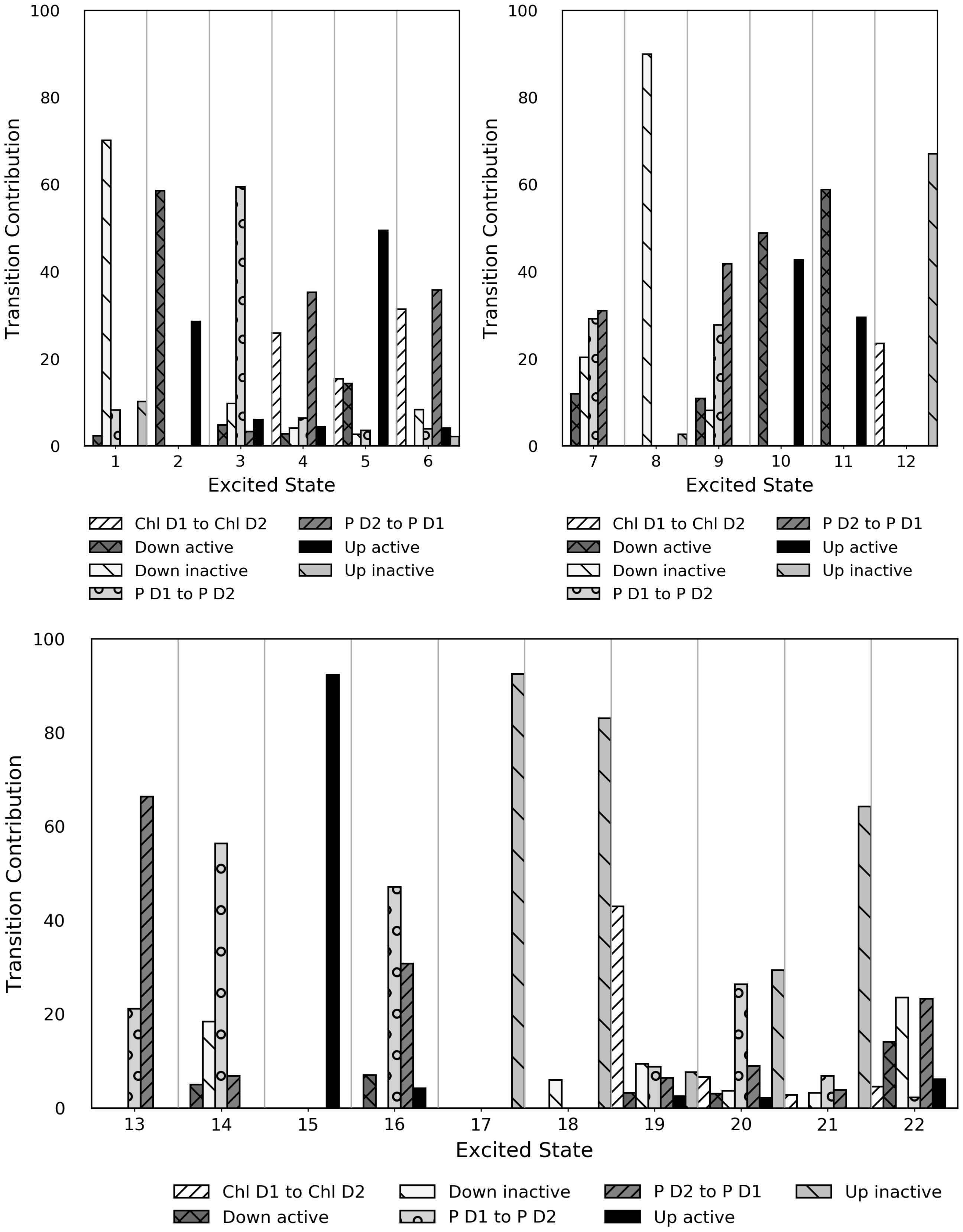

Figure S1 b) Showing % contribution of each type of transition for W-T model 1 (with 2 amino acids and truncated phytol chains): (top left) states 1-6, (top right) 7-12 and (bottom) states 13-22.

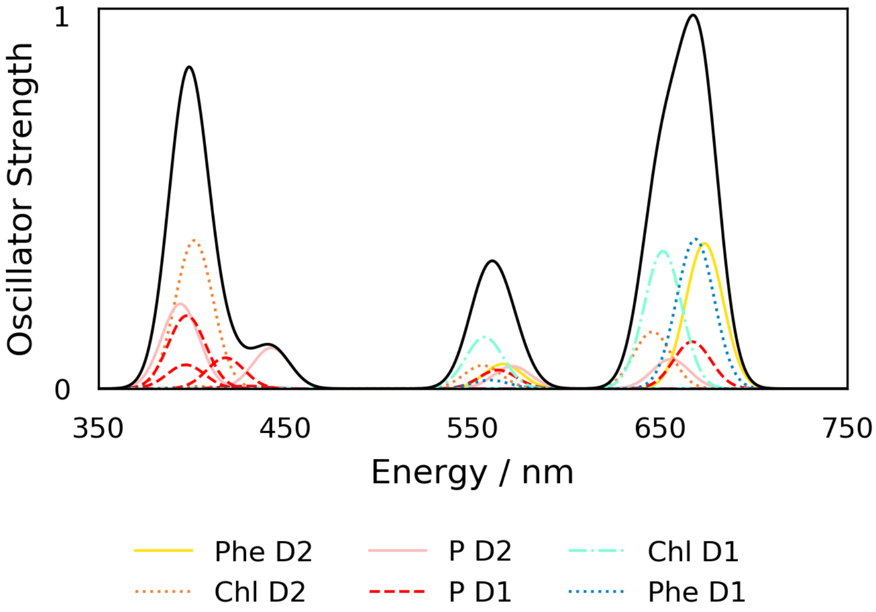

Figure S1 c) Simulated Absorption spectrum for model 1 (2 amino acids, truncated phytol chains) : Black solid spectrum – overall calculated absorption spectrum, Colored lines – single excited states are colored according to the cofactor of the dominant transition as in table 1 and figure 2: Phe_{D1} – Blue, Chl_{D1} – Aquamarine, P_{D1} – Red, P_{D2} – Pink, Chl_{D2} – Orange and Phe_{D2} – Orange.

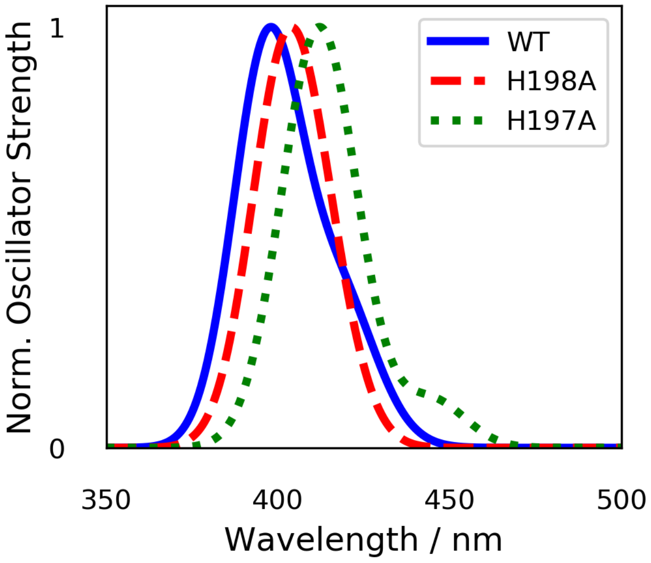

Figure S1 d) Simulated P_{D1} absorption bands in the Soret region obtained using model 1 (2 amino acids, truncated phytol chains) for the W-T RC and two mutants His-198-Ala and His-197-Ala. Sum of the gaussian broadened excited states which involve P_{D1} excitation for W-T (blue solid line), His-198-Ala (red dashed line) and His-197-Ala (green dotted line) where peak heights have been normalized to one for ease of comparison in the energy shifts.

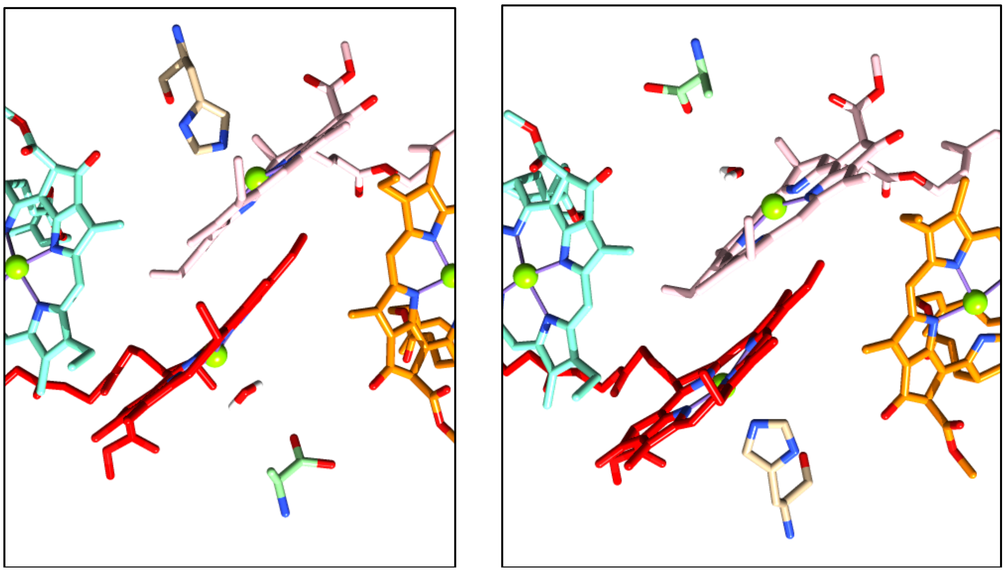

Figure S1 e) View of the P chlorophylls with either His at D1-198 (left) or at D2-197 (right) mutated to Ala with a water molecule inserted to ligate to the chlorophyll center for model 1 (2 amino acids, truncated phytol chains): (left) H198A mutant and (right) H197A mutant. P_{D1} (red), P_{D2} (pink), mutated residue His to Ala (green)

Model 2: wB97x-D/6-31Gdp (2 amino acids, full phytol chains)

Table S2 a) State Energies and Oscillator Strengths for Model 2 (2 amino acids, full phytol chains) wb97xd/6-31G(d,p)

State	Energy / nm	Oscillator Strength
1	677.29	0.3075
2	670.53	0.4045
3	669.01	0.0977
4	657.06	0.0603
5	654.72	0.5015
6	646.4	0.1468
7	572.88	0.0688
8	569.13	0.0585
9	564.27	0.0561
10	562.26	0.0306
11	559.69	0.0891
12	556.92	0.1074
13	440.74	0.1045
14	432.94	0.0001
15	431.37	0.0105
16	418.72	0.1027
17	411.42	0.0003
18	399.83	0.3993
19	399.23	0.0906
20	399.04	0.0119
21	396.51	0.1682
22	394.07	0.3778

Table S2 b) Transitions and Transition Contributions for Model 2 (2 amino acids, full phytol chains) wb97xd/6-31G(d, p)

State	Occupied MO	Virtual MO	Transition Contribution	Transition %
1	1520	1532	-0.29337	17.2
1	1520	1538	-0.23476	11.0
1	1526	1532	0.52063	54.2
1	1526	1538	-0.17957	6.4
1	1528	1533	0.12648	3.2
2	1517	1531	0.13088	3.4
2	1519	1531	-0.22961	10.5
2	1519	1535	0.21424	9.2
2	1521	1529	-0.14309	4.1
2	1524	1529	-0.13293	3.5
2	1524	1531	0.52535	55.2

2	1524	1535	0.15683	4.9
3	1521	1529	0.23777	11.3
3	1525	1539	0.14642	4.3
3	1526	1532	-0.12073	2.9
3	1528	1533	0.50437	50.9
3	1528	1534	-0.2366	11.2
4	1518	1530	-0.12599	3.2
4	1518	1536	-0.17598	6.2
4	1521	1529	-0.19575	7.7
4	1522	1530	0.3105	19.3
4	1523	1530	0.32484	21.1
4	1525	1534	-0.12316	3.0
4	1527	1533	0.14121	4.0
4	1527	1534	0.28096	15.8
4	1528	1533	0.1398	3.9
5	1517	1529	-0.11198	2.5
5	1517	1537	0.1827	6.7
5	1518	1530	-0.10985	2.4
5	1518	1536	-0.12803	3.3
5	1521	1529	0.47295	44.7
5	1521	1531	0.10166	2.1
5	1522	1530	0.21529	9.3
5	1523	1530	0.22536	10.2
5	1524	1531	0.1189	2.8
5	1528	1533	-0.14472	4.2
6	1518	1536	0.11713	2.7
6	1521	1529	0.17078	5.8
6	1522	1530	-0.17712	6.3
6	1522	1540	-0.10672	2.3
6	1523	1530	-0.18577	6.9
6	1525	1534	-0.18978	7.2
6	1525	1539	-0.10459	2.2
6	1527	1533	0.22557	10.2
6	1527	1534	0.44465	39.5
7	1522	1533	0.12461	3.1
7	1522	1534	0.25088	12.6
7	1523	1533	-0.11618	2.7
7	1523	1534	-0.23548	11.1
7	1525	1533	0.1828	6.7
7	1525	1534	0.31798	20.2
7	1527	1534	0.18198	6.6
7	1527	1539	0.14589	4.3

7	1527	1540	0.21249	9.0
7	1528	1533	-0.11319	2.6
7	1528	1534	-0.25107	12.6
8	1520	1532	0.5003	50.1
8	1520	1538	-0.11179	2.5
8	1526	1532	0.3423	23.4
8	1526	1538	0.28051	15.7
9	1522	1533	-0.20615	8.5
9	1522	1534	0.11398	2.6
9	1523	1533	0.21258	9.0
9	1523	1534	-0.11575	2.7
9	1525	1533	0.38027	28.9
9	1525	1534	-0.15563	4.8
9	1527	1533	0.25389	12.9
9	1527	1534	-0.10344	2.1
9	1528	1539	-0.23267	10.8
9	1528	1540	0.14653	4.3
10	1517	1529	-0.10483	2.2
10	1517	1531	-0.2199	9.7
10	1519	1529	-0.17987	6.5
10	1519	1531	0.43714	38.2
10	1524	1531	0.29657	17.6
10	1524	1535	-0.27775	15.4
11	1518	1530	0.56686	64.3
11	1522	1530	0.14358	4.1
11	1522	1536	0.2012	8.1
11	1523	1530	0.15234	4.6
11	1523	1536	0.21086	8.9
12	1517	1529	0.53098	56.4
12	1519	1529	0.21714	9.4
12	1519	1531	0.17824	6.4
12	1521	1529	0.13318	3.5
12	1521	1537	-0.26041	13.6
13	1525	1533	-0.25585	13.1
13	1525	1534	0.19524	7.6
13	1525	1539	0.11844	2.8
13	1527	1533	0.42977	36.9
13	1527	1534	-0.13253	3.5
13	1527	1539	-0.23039	10.6
13	1528	1534	-0.24337	11.8
14	1525	1529	0.1141	2.6
14	1527	1529	0.12927	3.3

14	1528	1529	0.64754	83.9
14	1528	1531	0.13018	3.4
15	1522	1534	0.23725	11.3
15	1523	1534	-0.22479	10.1
15	1525	1533	-0.16594	5.5
15	1527	1533	0.11898	2.8
15	1528	1529	0.11938	2.9
15	1528	1533	0.22502	10.1
15	1528	1534	0.42939	36.9
15	1528	1540	0.14179	4.0
16	1516	1533	0.13139	3.5
16	1522	1533	0.14528	4.2
16	1523	1533	-0.1257	3.2
16	1525	1533	0.28112	15.8
16	1525	1534	-0.11018	2.4
16	1527	1533	0.11754	2.8
16	1527	1534	-0.12998	3.4
16	1527	1539	-0.13255	3.5
16	1527	1540	-0.21034	8.8
16	1528	1539	0.43349	37.6
16	1528	1540	-0.15987	5.1
17	1527	1530	0.59014	69.7
17	1528	1530	-0.33922	23.0
18	1518	1530	0.12301	3.0
18	1521	1537	0.18204	6.6
18	1522	1536	-0.18366	6.7
18	1522	1539	0.1063	2.3
18	1523	1536	-0.19005	7.2
18	1523	1539	-0.10225	2.1
18	1525	1533	0.11484	2.6
18	1525	1534	0.13862	3.8
18	1525	1540	0.19006	7.2
18	1526	1538	0.14201	4.0
18	1527	1529	0.29379	17.3
18	1527	1534	0.16644	5.5
18	1528	1533	0.12971	3.4
19	1522	1529	-0.13104	3.4
19	1522	1536	0.10631	2.3
19	1523	1529	0.1289	3.3
19	1523	1536	0.11255	2.5
19	1525	1540	-0.10122	2.0
19	1527	1529	0.53698	57.7

19	1527	1531	0.10787	2.3
19	1528	1529	-0.13367	3.6
20	1525	1530	-0.19293	7.4
20	1527	1530	0.33766	22.8
20	1528	1530	0.551	60.7
21	1516	1534	-0.1795	6.4
21	1518	1530	-0.12892	3.3
21	1520	1532	0.11094	2.5
21	1522	1534	-0.19349	7.5
21	1522	1536	0.18808	7.1
21	1523	1534	0.18125	6.6
21	1523	1536	0.19928	7.9
21	1525	1533	0.14652	4.3
21	1525	1534	0.27363	15.0
21	1526	1538	-0.22333	10.0
21	1527	1533	0.15062	4.5
21	1527	1540	0.14472	4.2
21	1528	1534	0.17966	6.5
22	1520	1532	-0.15753	5.0
22	1521	1537	-0.10476	2.2
22	1522	1533	0.1683	5.7
22	1522	1534	-0.16258	5.3
22	1522	1536	-0.11014	2.4
22	1522	1539	-0.11927	2.8
22	1523	1533	-0.1621	5.3
22	1523	1534	0.15874	5.0
22	1523	1536	-0.1192	2.8
22	1523	1539	0.1134	2.6
22	1526	1538	0.31185	19.5
22	1527	1533	0.20642	8.5
22	1527	1534	-0.13253	3.5
22	1527	1540	0.18358	6.7

Table S2 c) Cofactor % Contributions* to Molecular Orbitals for Model 2 (2 amino acids, full phytol chains) wb97xd/6-31G(d, p)

MO	Phe D1	Phe D2	P D1	P D2	Chl D1	Chl D2
1517	17.1	0.0	0.4	0.1	82.4	0.0
1518	0.0	0.5	0.0	0.2	0.0	99.2
1519	82.3	0.0	0.3	0.0	17.3	0.0
1520	0.0	98.9	0.0	0.3	0.0	0.8
1521	0.5	0.0	2.7	2.1	94.7	0.0
1522	0.0	0.3	23.2	26.5	2.5	47.4
1523	0.0	0.3	22.5	23.3	2.0	51.7
1524	99.2	0.0	0.4	0.0	0.4	0.0
1525	0.0	0.0	48.8	50.7	0.2	0.1
1526	0.0	99.7	0.0	0.2	0.0	0.1
1527	0.0	0.0	13.8	85.9	0.1	0.0
1528	0.0	0.0	89.4	10.4	0.1	0.0
1529	5.3	0.0	0.4	0.2	94.1	0.0
1530	0.0	2.4	0.0	0.2	0.0	97.4
1531	94.4	0.0	0.4	0.0	5.2	0.0
1532	0.0	97.5	0.0	0.3	0.0	2.2
1533	0.0	0.0	81.2	18.6	0.1	0.1
1534	0.0	0.0	17.9	81.8	0.1	0.1
1535	94.6	0.0	0.3	0.0	5.1	0.0
1536	0.0	0.5	0.0	0.4	0.0	99.1
1537	5.3	0.0	0.5	0.2	94.0	0.0
1538	0.0	99.2	0.0	0.6	0.0	0.2
1539	0.0	0.0	73.4	26.2	0.2	0.1
1540	0.0	0.0	27.2	72.2	0.3	0.2

* as determined by the Mulliken Charges method in MultiWfn

Table S2 d) Nearest atoms to centers of density depletion and increment from ground state to excited state (method as described in reference 57) for Model 2 (2 amino acids, full phytol chains) wb97xd/6-31G(d, p)

State	Nearest Atom to Center of Density Depletion			Nearest Atom to Center of Density Increment		
	Atom	Element	Cofactor	Atom	Element	Cofactor
1	750	N	Phe D2	750	N	Phe D2
2	665	H	Phe D1	612	N	Phe D1
3	41	Mg	P D1	41	Mg	P D1
4	64	C	P D1	64	C	P D1
5	475	C	P D2	475	C	P D2
6	452	Mg	P D2	452	Mg	P D2
7	457	N	P D2	452	Mg	P D2
8	731	N	Phe D2	816	H	Phe D2
9	46	N	P D1	41	Mg	P D1

10	665	H	Phe D1	665	H	Phe D1
11	183	N	Chl D2	178	Mg	Chl D2
12	320	N	Chl D1	315	Mg	Chl D1
13	452	Mg	P D2	57	N	P D1
14	41	Mg	P D1	347	N	Chl D1
15	41	Mg	P D1	468	N	P D2
16	472	C	P D2	65	N	P D1
17	452	Mg	P D2	210	N	Chl D2
18	59	C	P D1	474	C	P D2
19	60	C	P D1	347	N	Chl D1
20	61	C	P D1	210	N	Chl D2
21	59	C	P D1	123	H	P D1
22	526	H	P D2	62	C	P D1

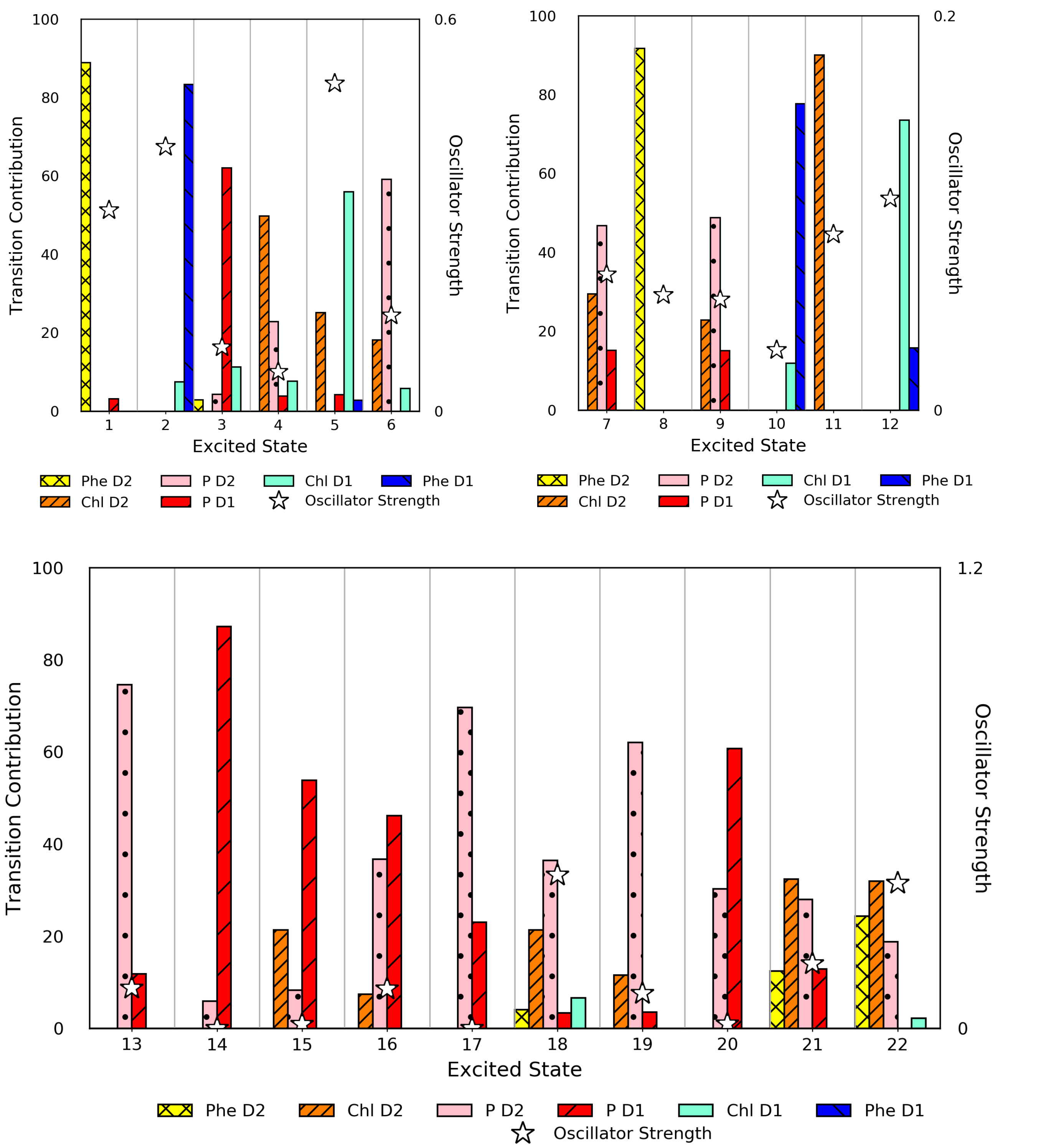

Figure S2 a) Showing the oscillator strengths (white stars) and % contribution of transitions from each cofactor excitation for W-T model 2 (2 amino acids and full phytol chains) : 2 (top left) states 1-6, (top right) 7-12 and (bottom) states 13-22

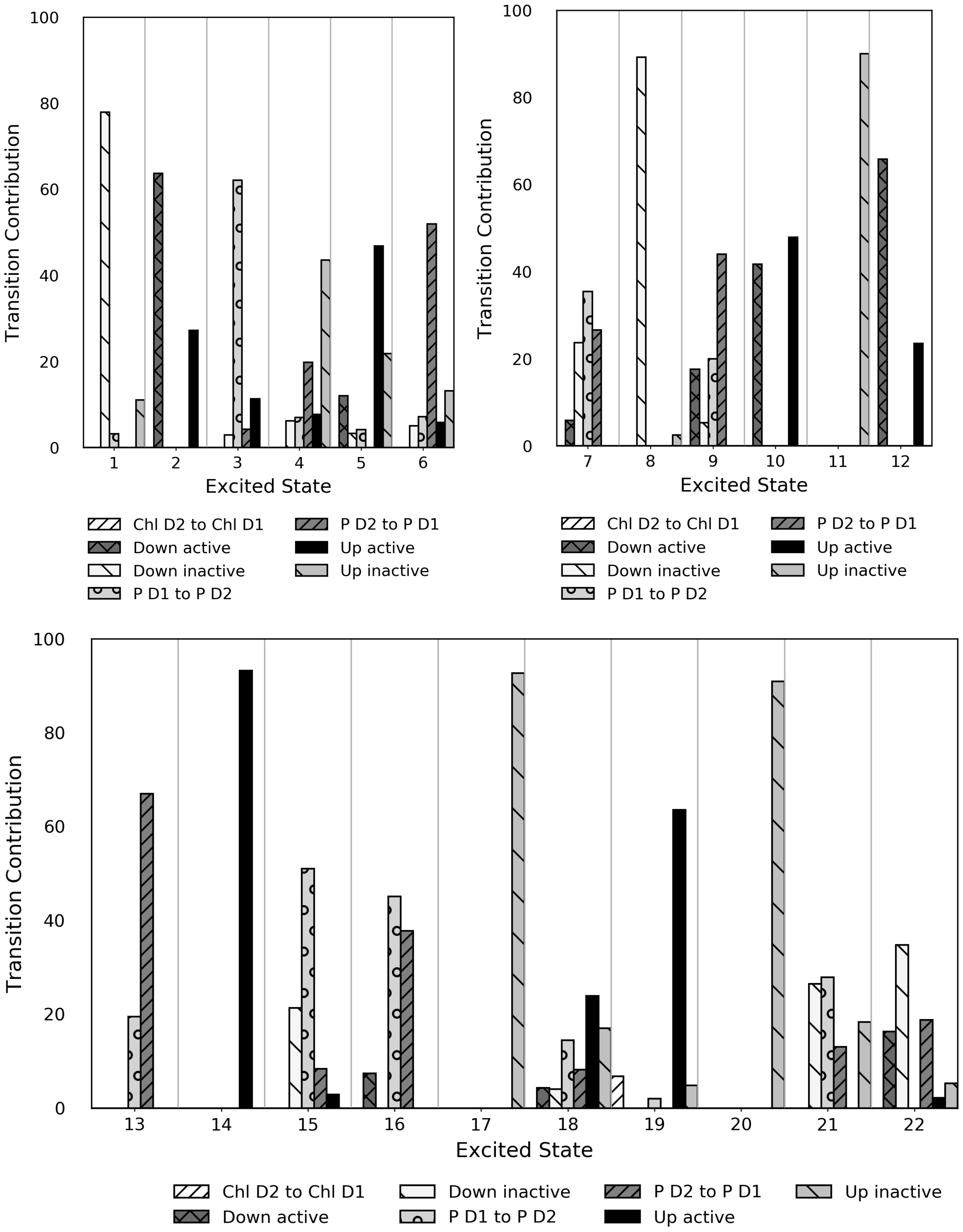

Figure S2 b) Showing % contribution of each type of transition for W-T model 2 (with 2 amino acids and full phytol chains) : (top left) states 1-6, (top right) 7-12 and (bottom) states 13-22.

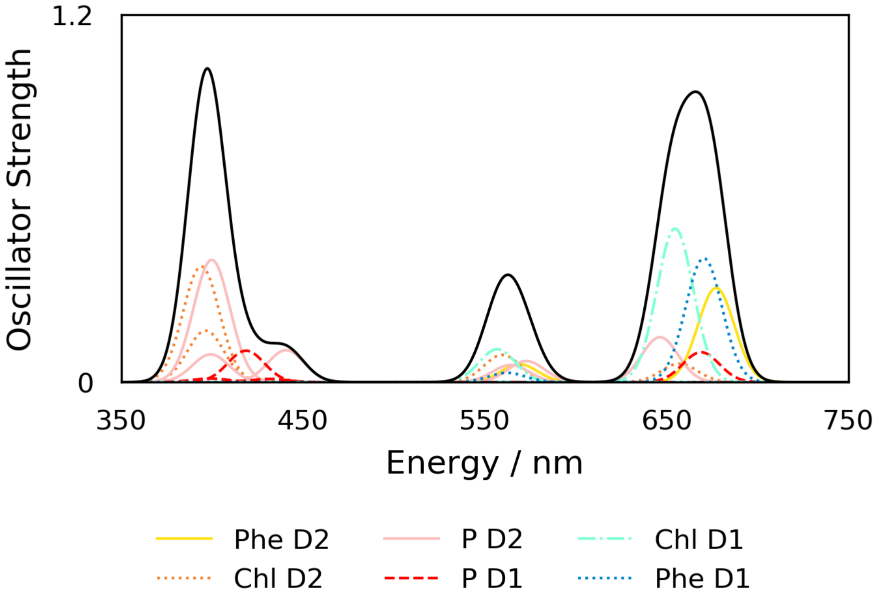

Figure S2 c) Simulated Absorption spectrum for model 2 (2 amino acids, full phytol chains) : Black solid spectrum – overall calculated absorption spectrum, Colored lines – single excited states are colored according to the cofactor of the dominant transition as in table 1 and figure 2: Phe_{D1} – Blue, Chl_{D1} – Aquamarine, P_{D1} – Red, P_{D2} – Pink, Chl_{D2} – Orange and Phe_{D2} – Orange.

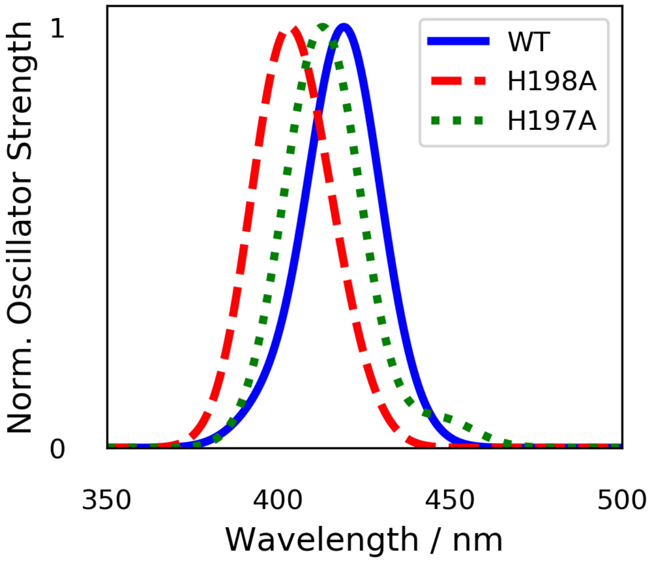

Figure S2 d) Simulated P_{D1} absorption bands in the Soret region obtained using model 2 (2 amino acids, full phytol chains) for the W-T RC and two mutants His-198-Ala and His-197-Ala. Sum of the gaussian broadened excited states which involve P_{D1} excitation for W-T (blue solid line), His-198-Ala (red dashed line) and His-197-Ala (green dotted line) where peak heights have been normalized to one for ease of comparison in the energy shifts.

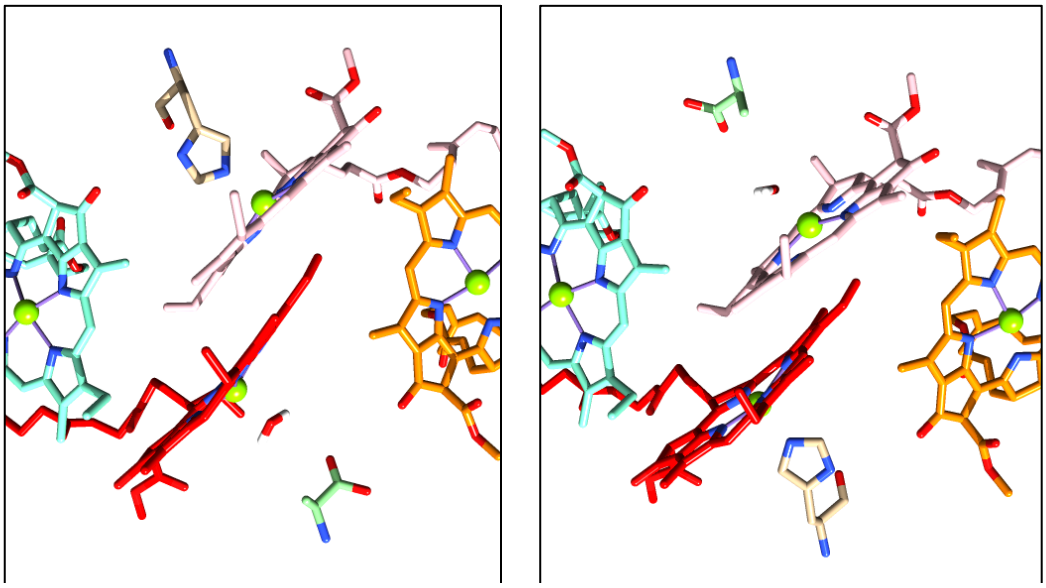

Figure S2 e) View of the P chlorophylls with either His at D1-198 (left) or at D2-197 (right) mutated to Ala with a water molecule inserted to ligate to the chlorophyll center for model 2 (2 amino acids, full phytol chains): (left) H198A mutant and (right) H197A mutant. P_{D1} (red), P_{D2} (pink), mutated residue His to Ala (green)

Model 3: wB97x-D/6-31Gdp (23 amino acids, truncated phytol chains)

Table S3 a) State Energies and Oscillator Strengths for Model 3 (23 amino acids, truncated phytol chains) wb97xd/6-31G(d,p)

State	Energy / nm	Oscillator Strength
1	684.92	0.4114
2	681.07	0.5523
3	673.9	0.0733
4	667.67	0.1158
5	657.23	0.3809
6	654.59	0.0847
7	583.28	0.0656
8	575.54	0.0796
9	570.86	0.0545
10	567.57	0.0371
11	564.05	0.1306
12	559.71	0.067
13	442.88	0.0728
14	435.25	0.0175
15	425.34	0.1684
16	411.55	0.0008
17	406.44	0.2816
18	404.36	0.0126
19	404.01	0.1872
20	400.24	0.0022
21	398.18	0.2467
22	397.6	0.0651

Table S3 b) Transitions and Transition Contributions for Model 3 (23 amino acids, truncated phytol chains) wb97xd/6-31G(d,p)

State	Occupied MO	Virtual MO	Transition Contribution	Transition %
1	1922	1932	-0.26554	14.1
1	1922	1940	-0.2116	9.0
1	1924	1930	0.17716	6.3
1	1927	1932	0.50935	51.9
1	1927	1940	-0.15112	4.6
1	1928	1934	0.1307	3.4
2	1918	1930	-0.20714	8.6
2	1918	1936	0.20239	8.2
2	1924	1930	0.47761	45.6
2	1924	1931	-0.15465	4.8
2	1924	1936	0.11467	2.6

2	1925	1930	-0.20296	8.2
2	1926	1933	0.1017	2.1
2	1927	1932	-0.1822	6.6
3	1920	1934	0.15568	4.8
3	1923	1937	0.15931	5.1
3	1925	1931	0.1833	6.7
3	1927	1932	-0.12295	3.0
3	1928	1934	0.56339	63.5
4	1920	1939	-0.12492	3.1
4	1921	1929	0.24231	11.7
4	1923	1933	-0.21818	9.5
4	1923	1939	-0.11695	2.7
4	1924	1930	-0.11558	2.7
4	1926	1933	0.50674	51.4
4	1928	1934	0.11047	2.4
5	1917	1935	-0.11198	2.5
5	1919	1931	-0.11691	2.7
5	1919	1938	0.1836	6.7
5	1921	1929	0.27352	15.0
5	1924	1930	0.139	3.9
5	1924	1931	0.12001	2.9
5	1925	1930	0.10354	2.1
5	1925	1931	0.49081	48.2
5	1928	1934	-0.13346	3.6
6	1917	1929	-0.1121	2.5
6	1917	1935	-0.20335	8.3
6	1921	1929	0.48341	46.7
6	1922	1929	-0.10391	2.2
6	1923	1933	0.11166	2.5
6	1925	1931	-0.21976	9.7
6	1926	1933	-0.25721	13.2
6	1928	1934	0.11344	2.6
7	1920	1933	-0.41465	34.4
7	1923	1933	-0.3584	25.7
7	1926	1933	-0.16057	5.2
7	1926	1937	-0.10326	2.1
7	1926	1939	-0.20878	8.7
7	1928	1933	0.25056	12.6
8	1922	1932	0.52712	55.6
8	1927	1932	0.34	23.1
8	1927	1940	0.24267	11.8
8	1927	1943	0.11502	2.6

9	1920	1934	-0.31295	19.6
9	1923	1934	0.41784	34.9
9	1926	1934	0.28116	15.8
9	1928	1934	0.12648	3.2
9	1928	1937	-0.24656	12.2
9	1928	1939	0.12708	3.2
10	1918	1930	0.41855	35.0
10	1918	1931	-0.20552	8.4
10	1919	1930	-0.28156	15.9
10	1919	1931	-0.18345	6.7
10	1924	1930	0.22481	10.1
10	1924	1931	-0.11272	2.5
10	1924	1936	-0.21832	9.5
10	1925	1938	0.10911	2.4
11	1918	1930	0.27048	14.6
11	1918	1931	0.13852	3.8
11	1919	1931	0.51331	52.7
11	1924	1930	0.13241	3.5
11	1925	1931	0.12433	3.1
11	1925	1938	-0.21602	9.3
12	1917	1929	-0.59764	71.4
12	1921	1929	-0.16613	5.5
12	1921	1935	-0.28353	16.1
12	1921	1941	-0.10175	2.1
13	1920	1937	0.10394	2.2
13	1923	1934	-0.2814	15.8
13	1923	1937	0.10003	2.0
13	1926	1933	0.14505	4.2
13	1926	1934	0.38442	29.6
13	1926	1937	-0.22117	9.8
13	1928	1933	-0.27277	14.9
13	1928	1934	0.13435	3.6
13	1928	1939	-0.11432	2.6
14	1920	1933	0.33422	22.3
14	1920	1934	-0.11962	2.9
14	1923	1934	-0.19744	7.8
14	1926	1934	0.20481	8.4
14	1928	1933	0.45697	41.8
14	1928	1939	0.11595	2.7
15	1920	1933	0.16414	5.4
15	1923	1934	0.31721	20.1
15	1926	1934	0.13222	3.5

15	1926	1939	-0.20719	8.6
15	1928	1937	0.4465	39.9
15	1928	1939	-0.13658	3.7
16	1926	1929	0.60456	73.1
16	1928	1929	-0.31442	19.8
17	1910	1933	0.12157	3.0
17	1917	1929	-0.11568	2.7
17	1920	1933	0.19917	7.9
17	1920	1937	-0.11397	2.6
17	1921	1935	0.26104	13.6
17	1923	1933	-0.31981	20.5
17	1923	1939	-0.19887	7.9
17	1926	1933	-0.17709	6.3
17	1928	1933	-0.24892	12.4
17	1928	1937	-0.13796	3.8
18	1847	1929	0.39374	31.0
18	1847	1935	-0.1545	4.8
18	1847	1941	0.42622	36.3
18	1870	1929	0.15711	4.9
18	1870	1941	0.10786	2.3
18	1921	1935	0.11404	2.6
19	1917	1929	0.18927	7.2
19	1917	1941	0.1068	2.3
19	1920	1933	0.1536	4.7
19	1921	1935	-0.42146	35.5
19	1921	1941	0.11918	2.8
19	1923	1933	-0.22368	10.0
19	1926	1933	-0.10666	2.3
19	1927	1940	0.17095	5.8
19	1928	1933	-0.13827	3.8
20	1923	1929	0.19363	7.5
20	1926	1929	-0.30772	18.9
20	1928	1929	-0.57493	66.1
21	1920	1934	0.11515	2.7
21	1922	1932	-0.11248	2.5
21	1922	1940	-0.11058	2.4
21	1922	1943	-0.10279	2.1
21	1925	1938	-0.10915	2.4
21	1926	1934	0.13062	3.4
21	1926	1939	0.10773	2.3
21	1927	1929	-0.41767	34.9
21	1927	1940	0.3395	23.1

21	1927	1943	-0.16509	5.5
22	1918	1930	0.10699	2.3
22	1919	1930	-0.1027	2.1
22	1919	1931	-0.1067	2.3
22	1921	1935	-0.10405	2.2
22	1924	1936	0.3481	24.2
22	1925	1938	-0.31851	20.3
22	1927	1929	0.19864	7.9
22	1928	1931	0.24772	12.3

Table S3 c) Cofactor % Contributions* to Molecular Orbitals for Model 3 (23 amino acids, truncated phytol chains) wb97xd/6-31G(d, p)

MO	Phe $_{D1}$	Phe $_{D2}$	P $_{D1}$	P $_{D2}$	Chl $_{D1}$	Chl $_{D2}$
1917	0.00	0.36	0.03	0.16	0.00	98.98
1918	84.25	0.00	0.26	0.04	14.96	0.00
1919	15.77	0.00	0.19	0.63	82.53	0.00
1920	0.02	0.02	42.00	54.78	0.78	1.25
1921	0.00	3.77	0.60	0.83	0.01	94.54
1922	0.00	95.69	0.01	0.10	0.00	3.96
1923	0.02	0.00	50.26	48.29	0.42	0.06
1924	88.97	0.00	0.19	0.18	10.26	0.00
1925	9.74	0.00	0.10	1.83	88.07	0.00
1926	0.00	0.00	15.39	82.23	1.62	0.03
1927	0.00	99.31	0.01	0.07	0.00	0.08
1928	0.00	0.01	90.31	8.56	0.07	0.05
1929	0.00	0.64	0.02	0.18	0.00	98.78
1930	92.73	0.00	0.23	0.02	6.47	0.00
1931	6.47	0.00	0.20	0.68	92.10	0.00
1932	0.00	98.72	0.00	0.23	0.00	0.62
1933	0.01	0.11	3.50	94.57	0.56	0.09
1934	0.00	0.00	94.36	4.41	0.15	0.05
1935	0.00	0.14	0.06	0.31	0.00	99.10
1936	92.71	0.00	2.82	0.31	3.70	0.00
1937	4.38	0.00	75.96	14.11	4.33	0.09
1938	2.21	0.00	2.43	7.10	87.44	0.01
1939	0.03	0.28	19.04	75.82	3.59	0.15
1940	0.00	99.06	0.04	0.43	0.00	0.20

* as determined by the Mulliken Charges method in MultiWfn

Table S3 d) Nearest atoms to centers of density depletion and increment from ground state to excited state (method as described in reference 57) for Model 3 (23 amino acids, truncated phytol chains) wb97xd/6-31G(d, p)

State	Nearest Atom to Center of Density Depletion			Nearest Atom to Center of Density Increment		
	Atom	Element	Cofactor	Atom	Element	Cofactor
1	651	C	Phe D2	651	C	Phe D2
2	562	C	Phe D1	562	C	Phe D1
3	363	Mg	P D1	363	Mg	P D1
4	386	C	P D1	386	C	P D1
5	868	H	P D2	868	H	P D2
6	501	H	Chl D2	501	H	Chl D2
7	802	Mg	P D2	834	N	P D2
8	631	N	Phe D2	689	H	Phe D2
9	368	N	P D1	363	Mg	P D1
10	553	N	Phe D1	602	H	Phe D1
11	604	H	Phe D1	605	H	Phe D1
12	455	N	Chl D2	482	N	Chl D2
13	826	N	P D2	383	C	P D1
14	363	Mg	P D1	802	Mg	P D2
15	383	C	P D1	387	N	P D1
16	802	Mg	P D2	482	N	Chl D2
17	379	N	P D1	386	C	P D1
18	489	O	Chl D2	485	C	Chl D2
19	187	H	Val 202 (nearest Chl_{D2})	501	H	Chl D2
20	383	C	P D1	482	N	Chl D2
21	651	C	Phe D2	692	H	Phe D2
22	361	H	Leu 205 (nearest Chl_{D1})	358	H	Leu 205 (nearest Chl_{D1})

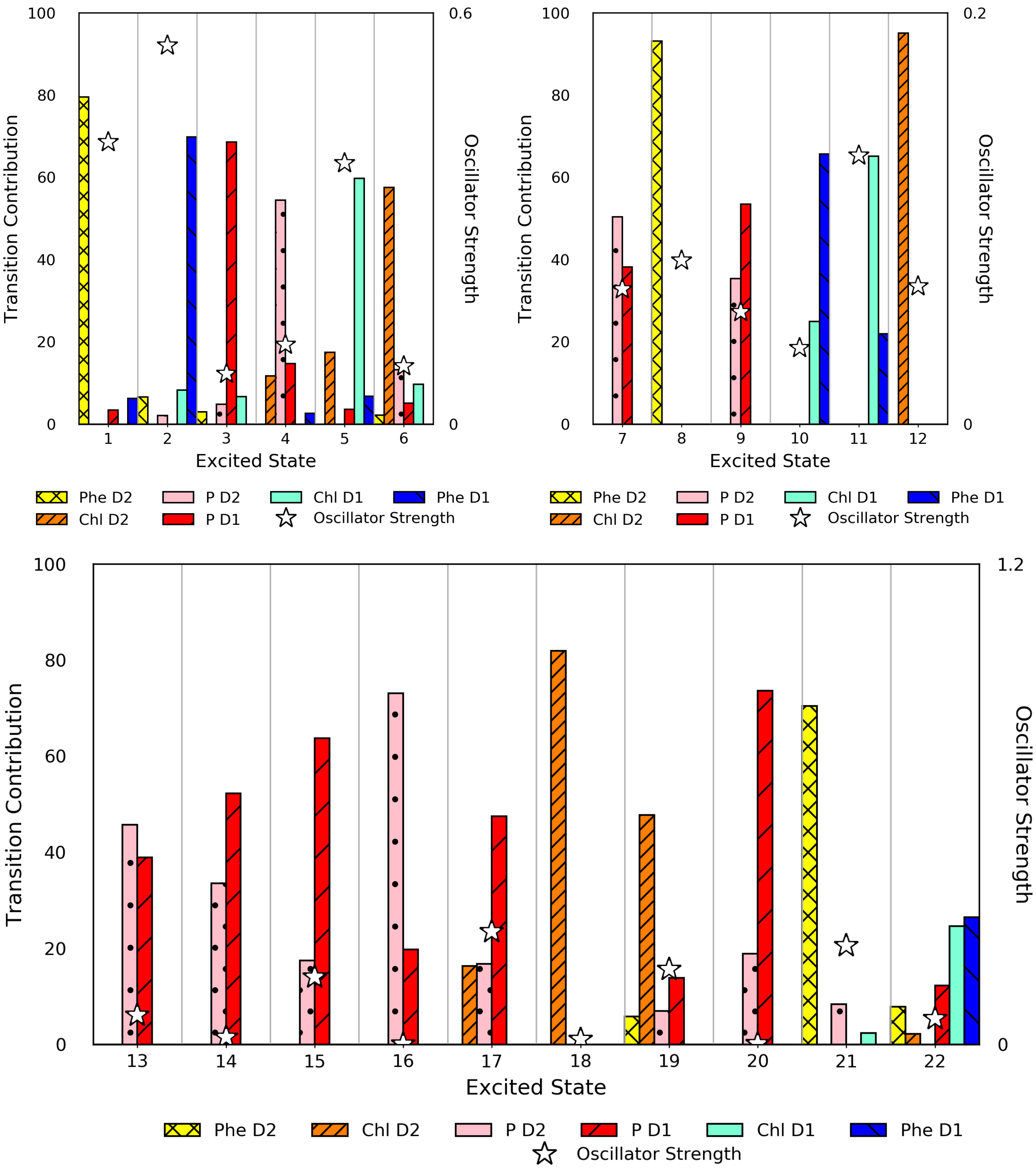

Figure S3 a) Showing the oscillator strengths (white stars) and % contribution of transitions from each cofactor excitation for W-T Model 3 (23 amino acids, truncated phytol chains) : (top left) states 1-6, (top right) 7-12 and (bottom) states 13-22

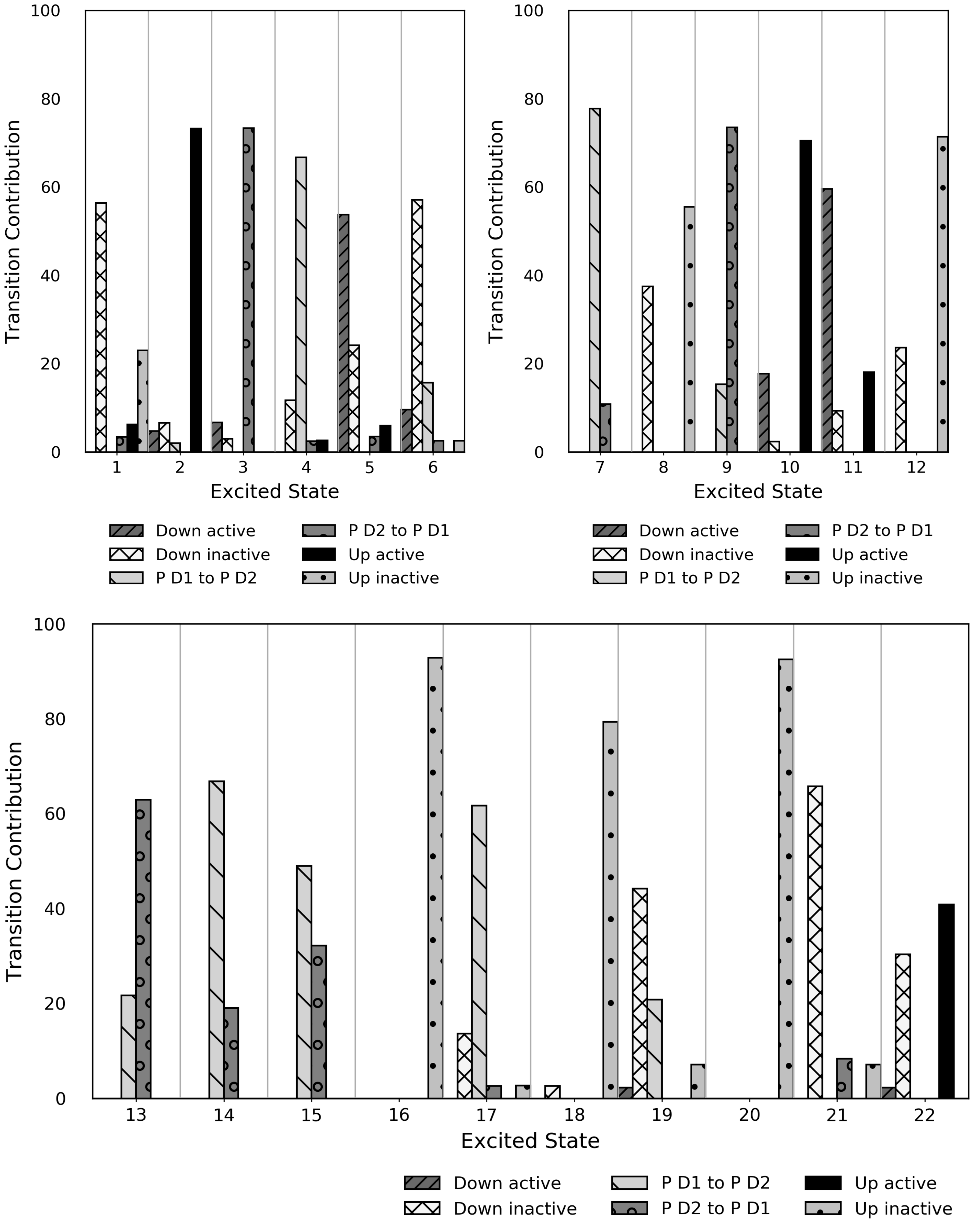

Figure S3 b) Showing % contribution of each type of transition for W-T Model 3 (23 amino acids, truncated phytol chains): (top left) states 1-6, (top right) 7-12 and (bottom) states 13-22.

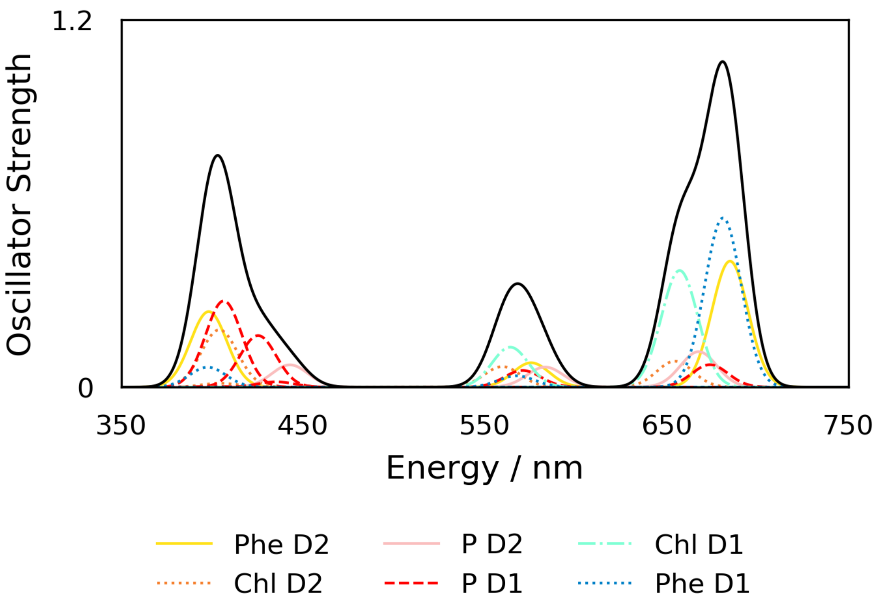

Figure S3 c) Simulated Absorption spectrum for Model 3 (23 amino acids, truncated phytol chains) : Black solid spectrum – overall calculated absorption spectrum, Colored lines – single excited states are colored according to the cofactor of the dominant transition as in table 1 and figure 2: Phe_{D1} – Blue, Chl_{D1} – Aquamarine, P_{D1} – Red, P_{D2} – Pink, Chl_{D2} – Orange and Phe_{D2} – Orange.

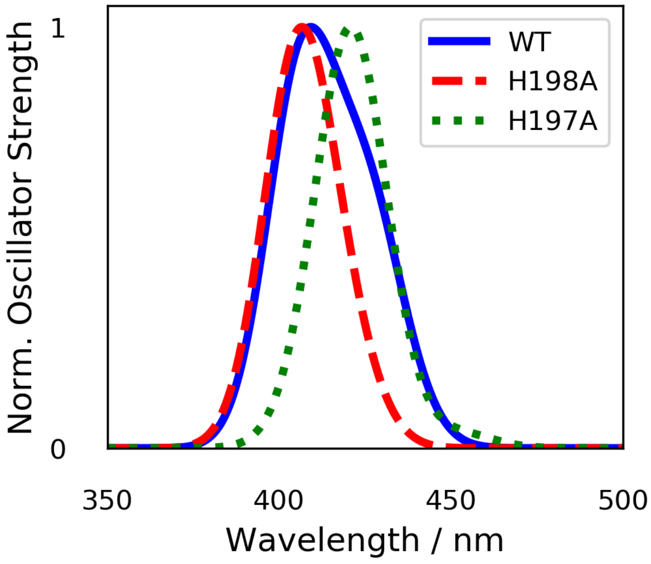

Figure S3 d) Simulated P_{D1} absorption bands in the Soret region obtained using Model 3 (23 amino acids, truncated phytol chains) for the W-T RC and two mutants His-198-Ala and His-197-Ala. Sum of the gaussian broadened excited states which involve P_{D1} excitation for W-T (blue solid line), His-198-Ala (red dashed line) and His-197-Ala (green dotted line) where peak heights have been normalized to one for ease of comparison in the energy shifts.

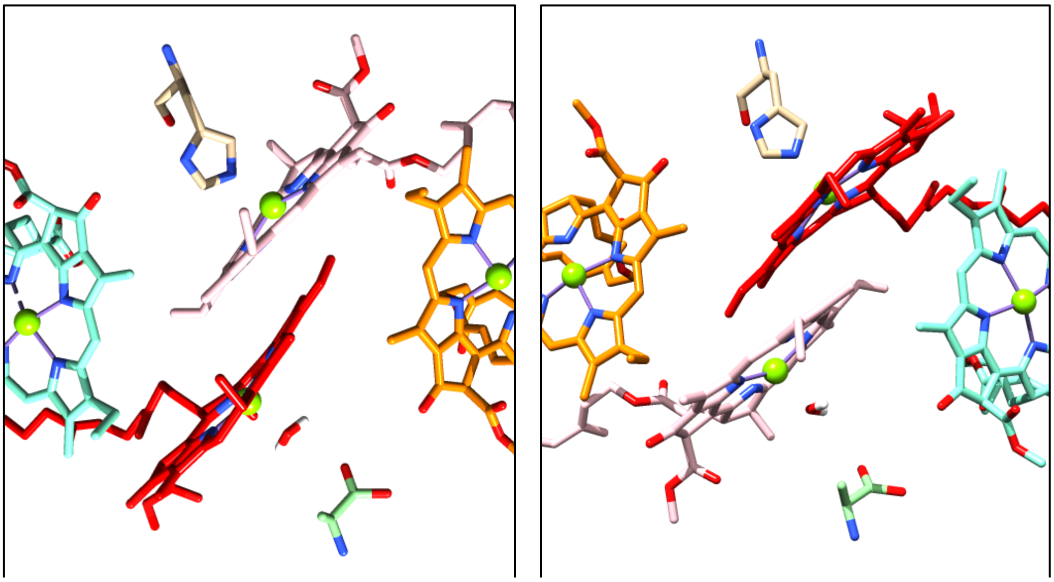

Figure S3 e) View of the P chlorophylls with either His at D1-198 (left) or at D2-197 (right) mutated to Ala with a water molecule inserted to ligate to the chlorophyll center for Model 3 (23 amino acids, truncated phytol chains): (left) H198A mutant and (right) H197A mutant. P_{D1} (red), P_{D2} (pink), mutated residue His to Ala (green)

Model 4: wB97x-D/6-31Gdp (23 amino acids, full phytol chains)

Table S4 a) State Energies and Oscillator Strengths for Model 4 (23 amino acids, full phytol chains) wb97xd/6-31G(d,p)

State	Energy/ nm	Oscillator Strength
**1**	692.46	0.3746
**2**	687.23	0.4202
**3**	675.93	0.0915
**4**	665.76	0.1809
**5**	660.4	0.4078
**6**	655.24	0.1396
**7**	584.79	0.0663
**8**	579.51	0.0825
**9**	576.34	0.0668
**10**	571.17	0.0383
**11**	565.19	0.1015
**12**	560.76	0.0715
**13**	443.03	0.0757
**14**	432.99	0.0329
**15**	426.15	0.1421
**16**	410.44	0.0008
**17**	404.14	0.4472
**18**	401.23	0.0377
**19**	400.68	0.0787
**20**	399.46	0.1436
**21**	398.55	0.3109
**22**	396	0.0051

Table S4 b) Transitions and Transition Contributions for Model 4 (23 amino acids, full phytol chains) wb97xd/6-31G(d,p)

State	Occupied MO	Virtual MO	Transition	Corrected transition %
1	2302	2313	-0.2421	11.7
1	2302	2320	0.22886	10.5
1	2308	2313	0.5695	64.9
1	2308	2320	0.12803	3.3
1	2310	2316	0.12381	3.1
2	2304	2316	-0.28135	15.8
2	2304	2323	-0.22127	9.8
2	2308	2313	-0.13326	3.6
2	2310	2316	0.5368	57.6
2	2310	2323	-0.16332	5.3
3	2305	2317	0.11999	2.9

3	2305	2318	-0.10539	2.2
3	2305	2322	-0.11314	2.6
3	2307	2322	0.13725	3.8
3	2309	2315	0.20869	8.7
3	2312	2317	0.43604	38.0
3	2312	2318	-0.37859	28.7
4	2301	2319	-0.11163	2.5
4	2306	2314	0.30362	18.4
4	2307	2317	-0.104	2.2
4	2307	2318	-0.15554	4.8
4	2307	2324	-0.12227	3.0
4	2309	2315	-0.20903	8.7
4	2311	2317	0.3183	20.3
4	2311	2318	0.33627	22.6
5	2301	2319	-0.12884	3.3
5	2303	2315	-0.13445	3.6
5	2303	2321	0.1768	6.3
5	2306	2314	0.32388	21.0
5	2309	2315	0.50323	50.6
6	2301	2314	-0.11915	2.8
6	2301	2319	-0.17642	6.2
6	2305	2324	0.10577	2.2
6	2306	2314	0.41857	35.0
6	2307	2318	0.1152	2.7
6	2309	2315	-0.21057	8.9
6	2311	2317	-0.25307	12.8
6	2311	2318	-0.27315	14.9
7	2305	2317	0.22874	10.5
7	2305	2318	0.26166	13.7
7	2307	2317	0.30204	18.2
7	2307	2318	0.28643	16.4
7	2311	2318	0.168	5.6
7	2311	2324	0.22537	10.2
7	2312	2317	-0.17912	6.4
7	2312	2318	-0.2221	9.9
8	2302	2313	0.55956	62.6
8	2303	2313	0.10336	2.1
8	2308	2313	0.2825	16.0
8	2308	2320	-0.24867	12.4
8	2308	2325	-0.11424	2.6
9	2304	2316	0.52004	54.1
9	2306	2316	0.10391	2.2

9	2310	2316	0.33836	22.9
9	2310	2323	0.25856	13.4
9	2310	2326	-0.10208	2.1
10	2305	2317	0.2916	17.0
10	2305	2318	-0.26813	14.4
10	2307	2317	-0.29812	17.8
10	2307	2318	0.24355	11.9
10	2311	2317	-0.206	8.5
10	2311	2318	0.1668	5.6
10	2312	2321	-0.12918	3.3
10	2312	2322	0.23331	10.9
11	2303	2315	-0.59612	71.1
11	2309	2315	-0.17527	6.1
11	2309	2321	0.23872	11.4
11	2309	2322	0.12181	3.0
12	2301	2314	-0.592	70.1
12	2306	2314	-0.18302	6.7
12	2306	2319	-0.28922	16.7
13	2298	2318	0.10966	2.4
13	2305	2322	0.10369	2.2
13	2307	2317	-0.19968	8.0
13	2307	2318	0.25601	13.1
13	2307	2322	0.10527	2.2
13	2311	2317	0.36923	27.3
13	2311	2318	-0.25881	13.4
13	2311	2321	0.10058	2.0
13	2311	2322	-0.20082	8.1
13	2312	2318	-0.20013	8.0
14	2298	2317	0.10201	2.1
14	2305	2317	-0.17986	6.5
14	2305	2318	-0.26612	14.2
14	2307	2317	0.15793	5.0
14	2307	2318	-0.16933	5.7
14	2311	2324	-0.10366	2.1
14	2312	2317	-0.34067	23.2
14	2312	2318	-0.34748	24.1
14	2312	2324	-0.14993	4.5
15	2298	2317	-0.11309	2.6
15	2305	2317	-0.20264	8.2
15	2305	2318	-0.12253	3.0
15	2307	2317	-0.22628	10.2
15	2307	2318	0.15877	5.0

15	2311	2318	0.16771	5.6
15	2311	2324	0.18963	7.2
15	2312	2318	-0.14906	4.4
15	2312	2321	0.22246	9.9
15	2312	2322	-0.41341	34.2
16	2311	2314	-0.61953	76.8
16	2311	2319	0.10009	2.0
16	2312	2314	0.29285	17.2
17	2298	2318	-0.15031	4.5
17	2305	2317	-0.20644	8.5
17	2305	2318	-0.10371	2.2
17	2305	2322	0.15815	5.0
17	2306	2319	-0.17813	6.3
17	2307	2317	0.18121	6.6
17	2307	2318	0.15666	4.9
17	2307	2324	0.18267	6.7
17	2309	2321	0.19384	7.5
17	2311	2318	0.17295	6.0
17	2312	2315	-0.18046	6.5
17	2312	2317	0.19512	7.6
17	2312	2322	0.11897	2.8
18	2306	2319	0.15178	4.6
18	2307	2315	-0.10238	2.1
18	2308	2320	0.13301	3.5
18	2309	2321	-0.12437	3.1
18	2310	2323	-0.1195	2.9
18	2311	2315	-0.12527	3.1
18	2312	2315	-0.58044	67.4
19	2298	2317	-0.11291	2.5
19	2298	2318	-0.17356	6.0
19	2301	2314	-0.11553	2.7
19	2305	2318	-0.27454	15.1
19	2306	2319	0.24346	11.9
19	2307	2317	0.17388	6.0
19	2307	2318	0.13073	3.4
19	2310	2323	-0.19576	7.7
19	2311	2317	0.18249	6.7
19	2311	2324	0.16304	5.3
19	2312	2315	0.23131	10.7
19	2312	2318	0.14503	4.2
20	2302	2313	0.16627	5.5
20	2302	2320	-0.14197	4.0

20	2302	2325	-0.1108	2.5
20	2305	2317	-0.12915	3.3
20	2306	2319	-0.12734	3.2
20	2308	2320	0.46564	43.4
20	2308	2325	-0.14601	4.3
20	2309	2321	-0.20803	8.7
20	2309	2322	-0.11324	2.6
20	2310	2323	0.10223	2.1
20	2312	2315	0.1213	2.9
21	2304	2316	0.14421	4.2
21	2305	2317	-0.19039	7.2
21	2305	2318	0.22044	9.7
21	2305	2322	0.15281	4.7
21	2306	2319	0.20248	8.2
21	2310	2323	-0.34734	24.1
21	2310	2326	-0.1003	2.0
21	2311	2317	-0.13469	3.6
21	2311	2318	0.19263	7.4
21	2311	2324	-0.13153	3.5
21	2312	2317	0.10392	2.2
22	2307	2314	0.2069	8.6
22	2311	2314	-0.2949	17.4
22	2312	2314	-0.5812	67.6

Table S4 c) Cofactor % Contributions* to Molecular Orbitals for Model 4 (23 amino acids, full phytol chains) wb97xd/6-31G(d, p)

MO	Phe $_{D1}$	Phe $_{D2}$	P $_{D1}$	P $_{D2}$	Chl $_{D1}$	Chl $_{D2}$
2298	0.0	0.0	70.3	26.7	0.0	0.3
2301	0.0	0.3	0.0	0.2	0.0	99.0
2302	96.9	0.0	0.7	0.0	2.0	0.0
2303	2.6	0.0	0.2	0.2	96.0	0.0
2304	0.0	94.9	0.4	0.6	0.0	3.8
2305	0.0	0.9	56.9	40.2	0.6	0.2
2306	0.0	3.6	0.4	0.3	0.0	95.4
2307	0.3	0.0	40.7	57.6	0.2	0.2
2308	97.9	0.0	0.5	0.2	1.0	0.0
2309	0.4	0.0	0.4	0.9	98.0	0.0
2310	0.0	99.1	0.0	0.3	0.0	0.1
2311	0.0	0.1	12.0	86.6	0.5	0.0
2312	0.0	0.0	87.7	11.2	0.1	0.1
2313	98.3	0.0	0.4	0.0	0.7	0.0
2314	0.0	0.8	0.0	0.2	0.0	98.6
2315	0.8	0.0	0.5	0.4	97.8	0.0
2316	0.0	98.7	0.0	0.2	0.0	0.7
2317	0.0	0.0	56.1	42.5	0.3	0.1
2318	0.0	0.0	42.2	56.7	0.1	0.1
2319	0.0	0.5	0.1	0.3	0.0	98.8
2320	98.4	0.0	0.3	0.0	0.8	0.0
2321	0.8	0.0	20.4	0.9	76.9	0.0
2322	0.2	0.0	69.1	9.2	20.4	0.1
2323	0.0	99.0	0.0	0.6	0.0	0.2
2324	0.0	0.0	10.4	87.9	0.6	0.1
2325	98.0	0.0	0.2	0.0	0.2	0.0
2326	0.0	98.4	0.0	0.2	0.0	0.3

* as determined by the Mulliken Charges method in MultiWfn

Table S4 d) Nearest atoms to centers of density depletion and increment from ground state to excited state (method as described in reference 57) for Model 4 (full phytol chains, 23 amino acids)

State	Nearest Atom to Center of Density Depletion			Nearest Atom to Center of Density Increment		
	Atom	Element	Cofactor	Atom	Element	Cofactor
1	709	N	Phe D1	709	N	Phe D1
2	847	N	Phe D2	847	N	Phe D2
3	138	Mg	P D1	138	Mg	P D1
4	161	C	P D1	160	C	P D1
5	572	C	P D2	572	C	P D2
6	161	C	P D1	161	C	P D1
7	549	Mg	P D2	549	Mg	P D2
8	690	N	Phe D1	777	H	Phe D1
9	828	N	Phe D2	913	H	Phe D2
10	143	N	P D1	170	N	P D1
11	417	N	Chl D1	412	Mg	Chl D1
12	280	N	Chl D2	275	Mg	Chl D2
13	549	Mg	P D2	154	N	P D1
14	138	Mg	P D1	549	Mg	P D2
15	158	C	P D1	141	C	P D1
16	549	Mg	P D2	307	N	Chl D2
17	154	N	P D1	569	C	P D2
18	138	Mg	P D1	444	N	Chl D1
19	156	C	P D1	566	C	P D2
20	688	C	Phe D1	688	C	Phe D1
21	623	H	P D2	216	H	P D1
22	158	C	P D1	307	N	Chl D2

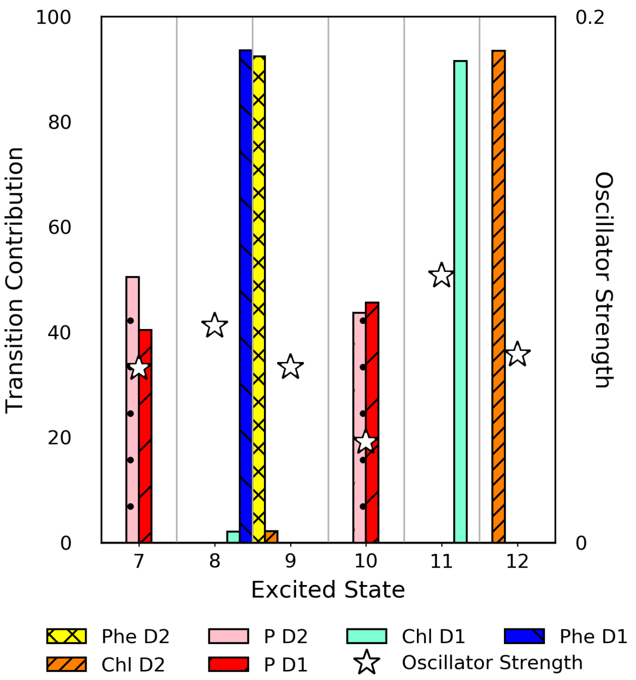

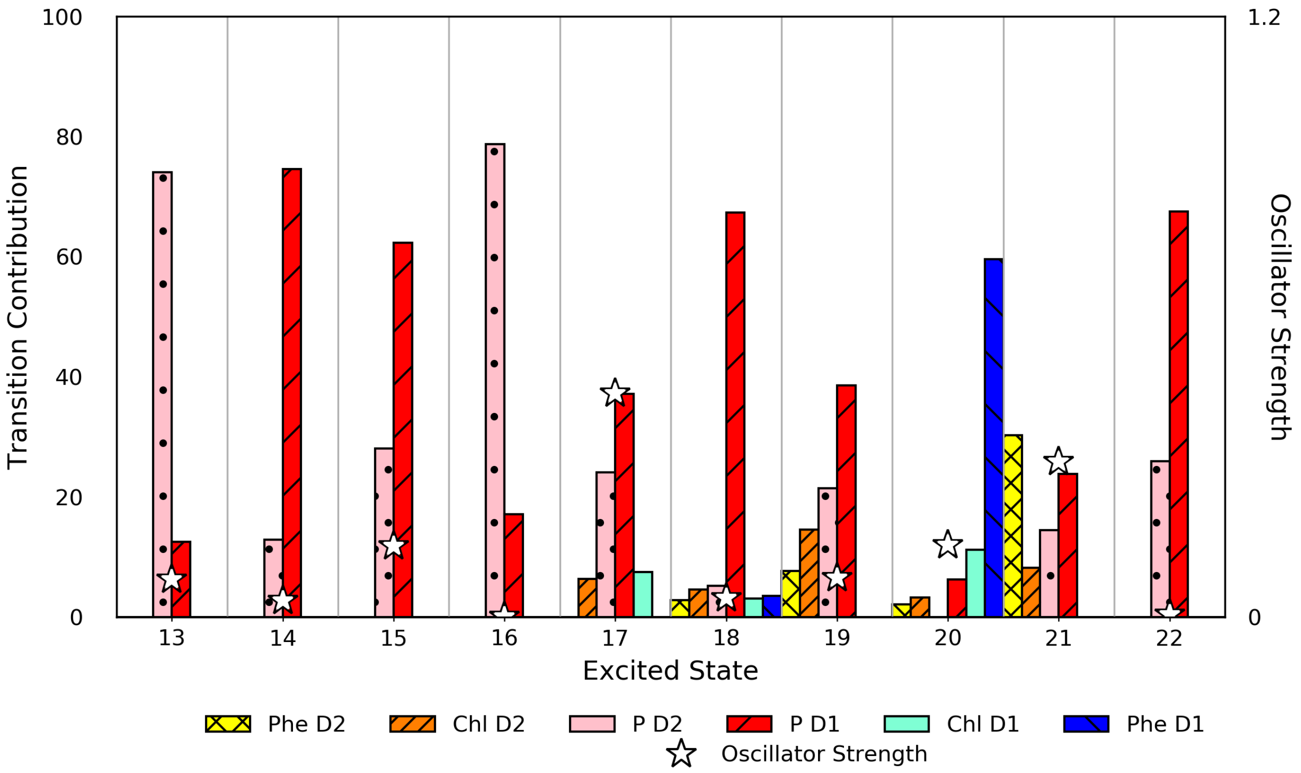

Figure S4 a) Showing the oscillator strengths (white stars) and % contribution of transitions from each cofactor excitation for W-T model 4 (23 amino acids and full phytol chains) : 4 (top) states 7-12 and (bottom) states 13-22

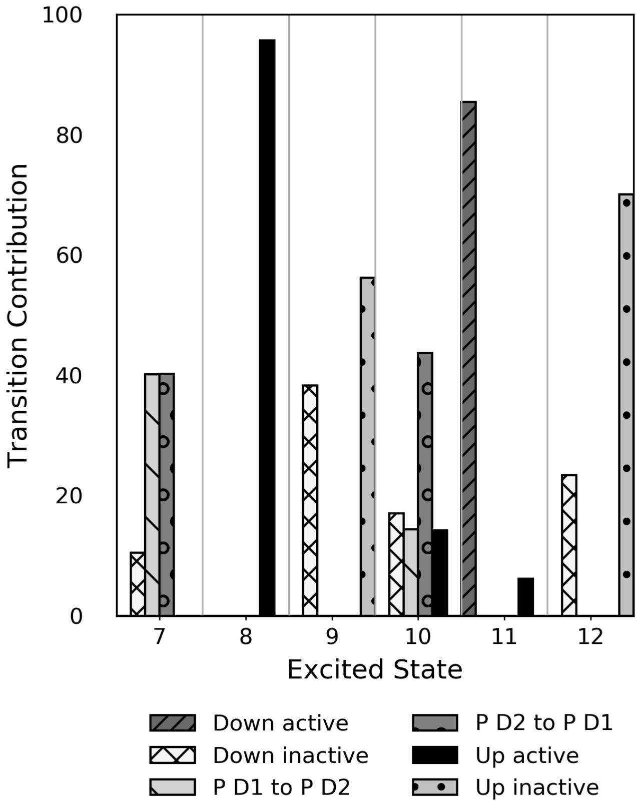

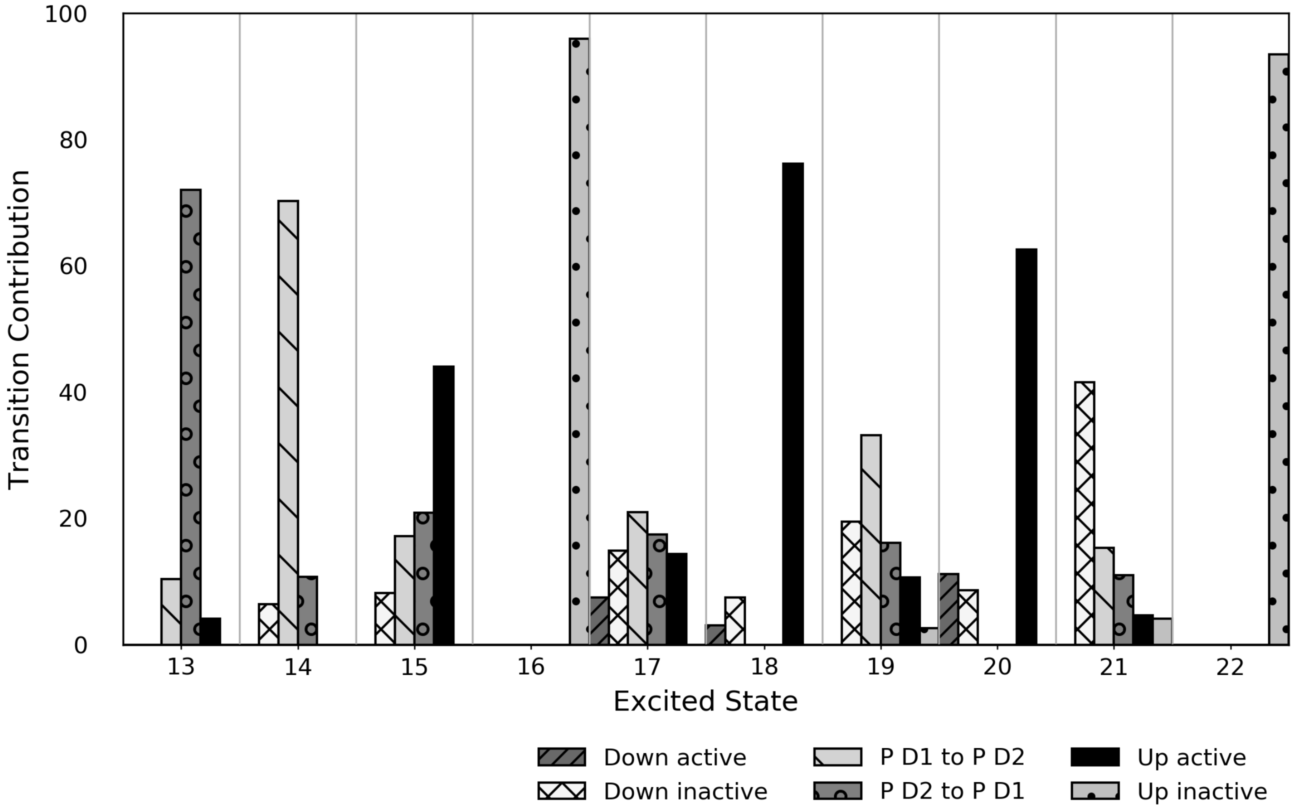

Figure S4 b) Showing % contribution of each type of transition for W-T model 4 (23 amino acids and full phytol chains) : (top) states 7-12 and (bottom) states 13-22

Occupied orbital: 2298

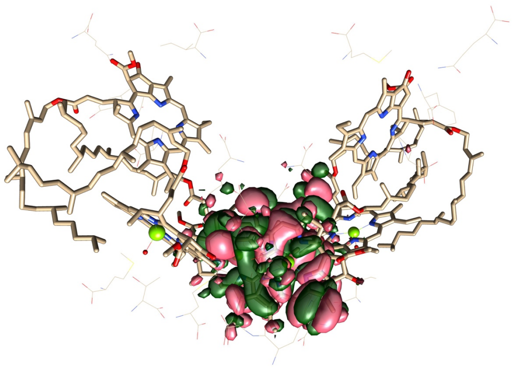

Occupied orbital: 2301

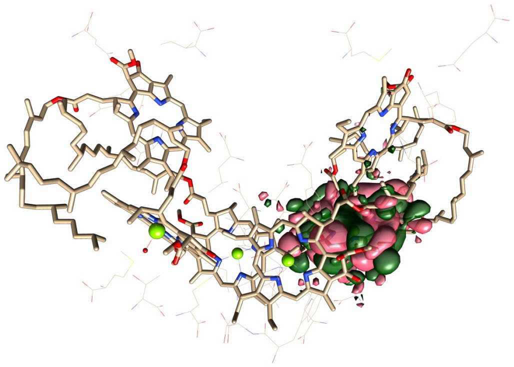

Occupied orbital: 2302

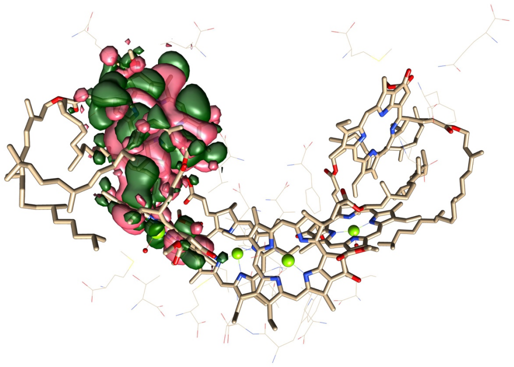

Occupied orbital: 2303

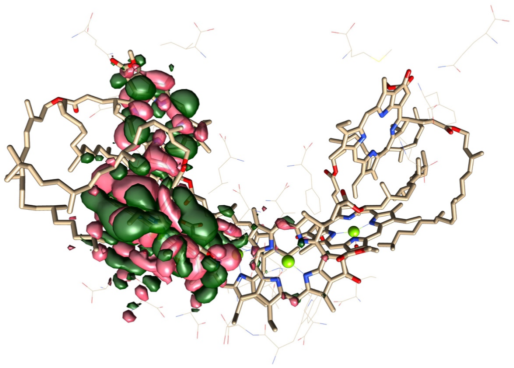

Occupied orbital: 2304

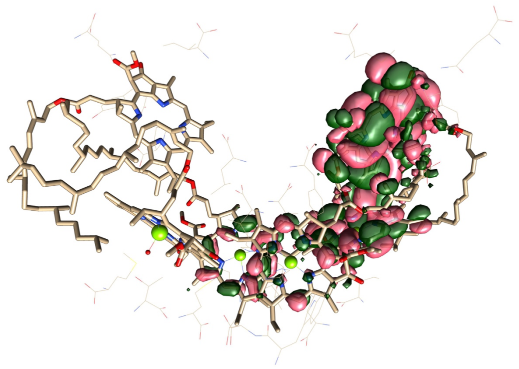

Occupied orbital: 2305

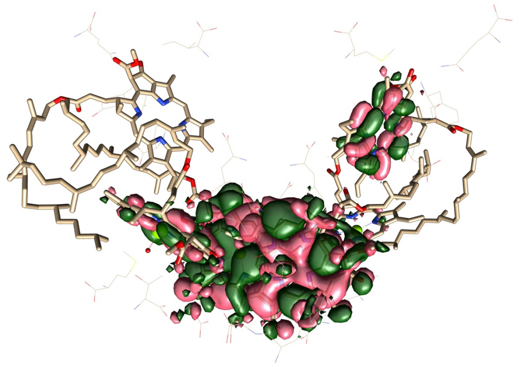

Occupied orbital: 2306

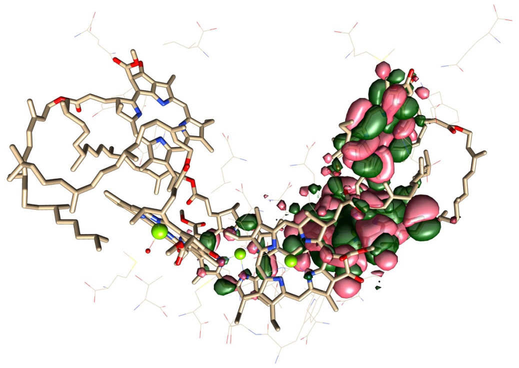

Occupied orbital: 2307

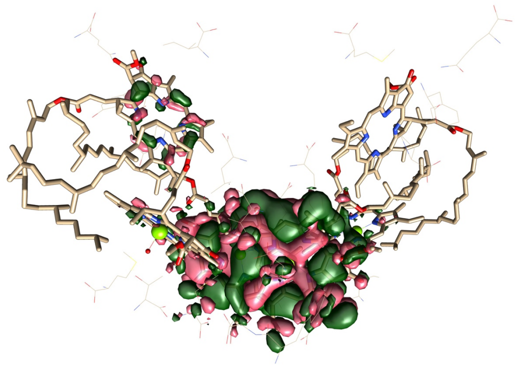

Figure S4 c) (page 1): Molecular Orbital Surfaces for Model 4 (23 amino acids, full phytol chains) wb97xd/6-31G(d, p) with iso-surface value +/- 0.002

Occupied orbital: 2308

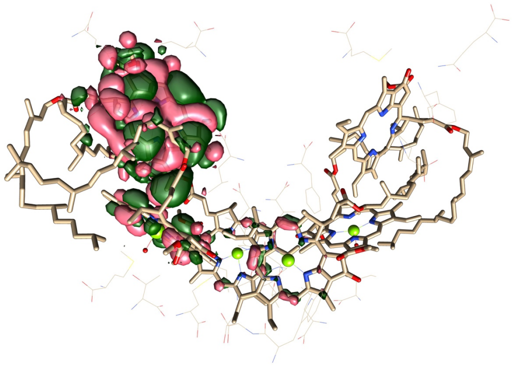

Occupied orbital: 2309

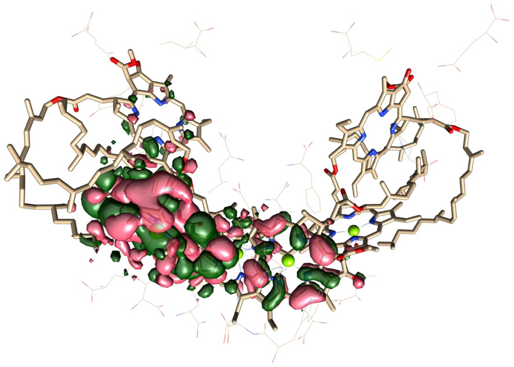

Occupied orbital: 2310

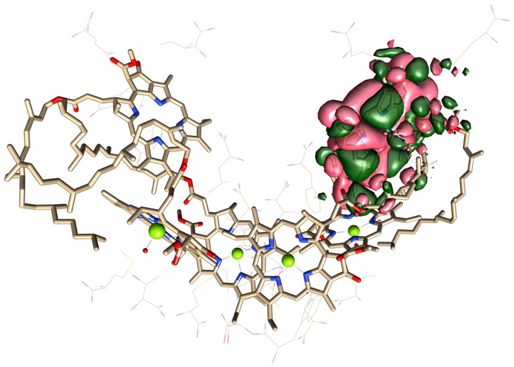

Occupied orbital: 2311

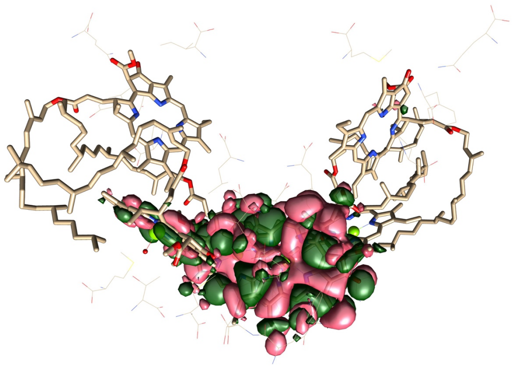

Occupied orbital: 2312

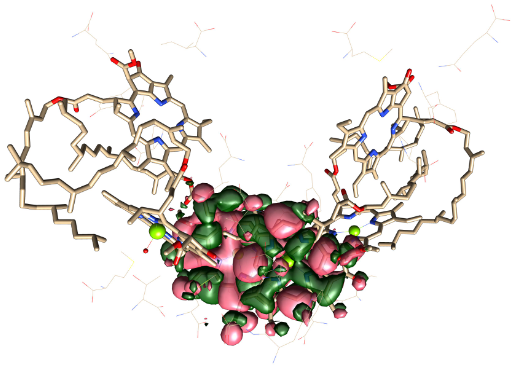

Figure S4 c) (page 2): Molecular Orbital Surfaces for Model 4 (23 amino acids, full phytol chains) wb97xd/6-31G(d, p) with iso-surface value +/- 0.002

Virtual orbital: 2313

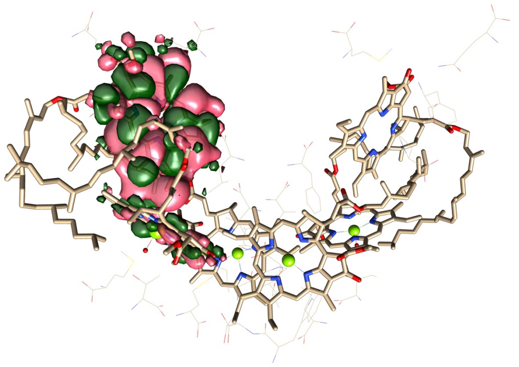

Virtual orbital: 2314

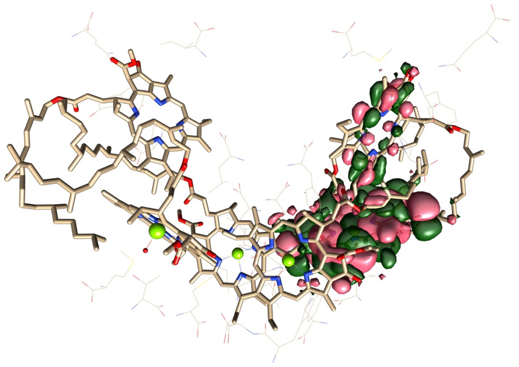

Virtual orbital: 2315

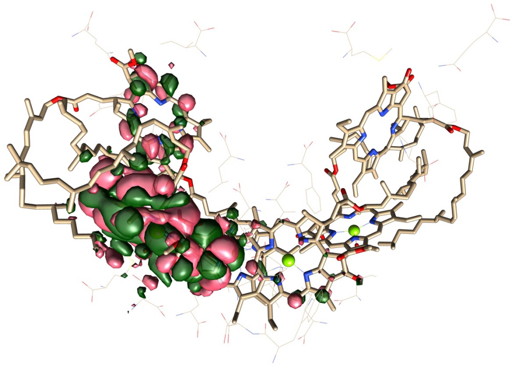

Virtual orbital: 2316

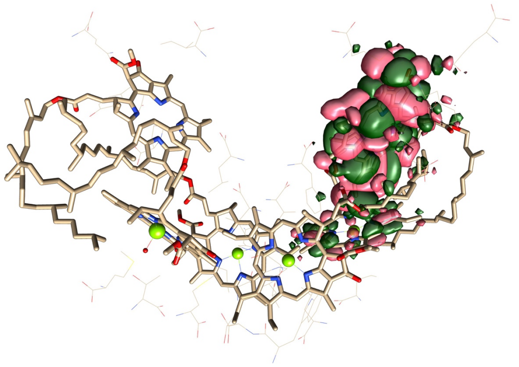

Virtual orbital: 2317

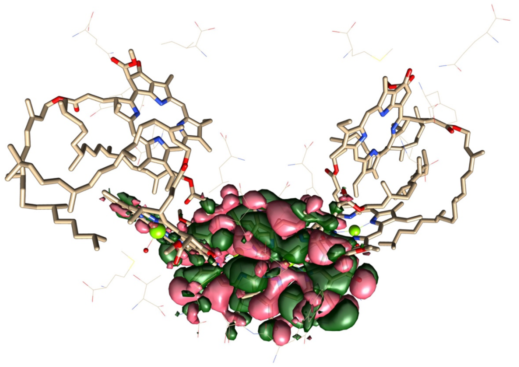

Virtual orbital: 2318

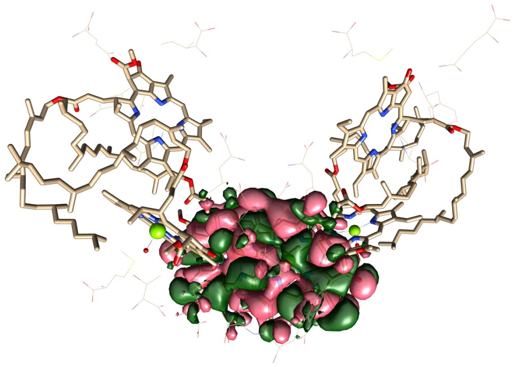

Virtual orbital: 2319

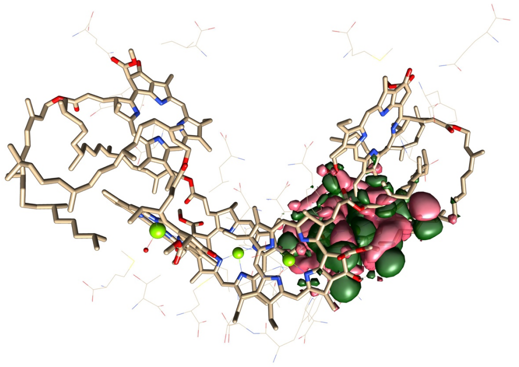

Virtual orbital: 2320

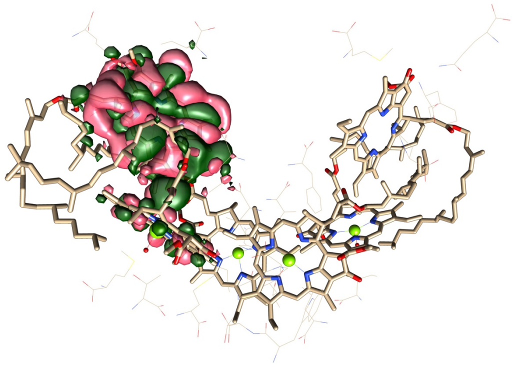

Figure S4 c) (page 3): Molecular Orbital Surfaces for Model 4 (23 amino acids, full phytol chains) wb97xd/6-31G(d, p) with iso-surface value +/- 0.002

Virtual orbital: 2321

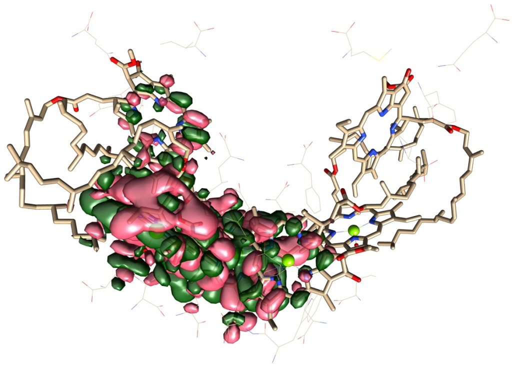

Virtual orbital: 2322

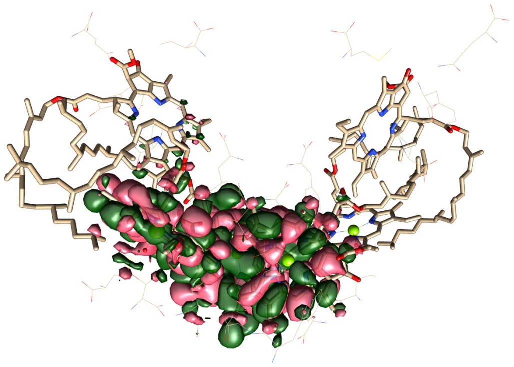

Virtual orbital: 2323

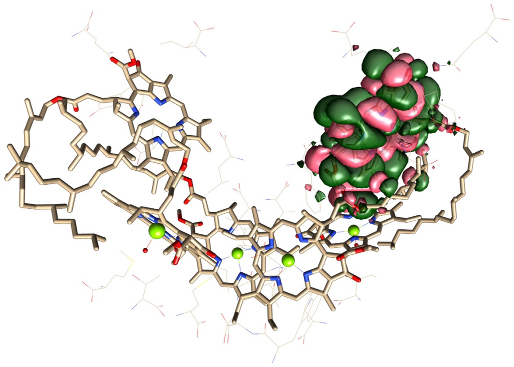

Virtual orbital: 2325

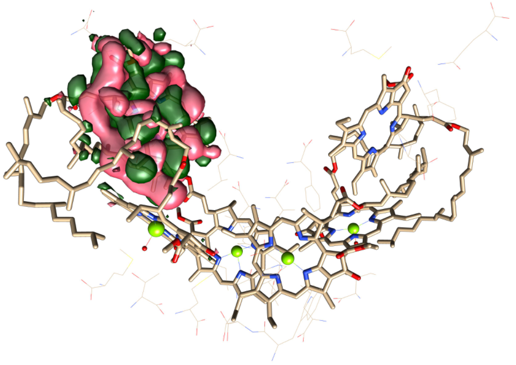

Virtual orbital: 2324

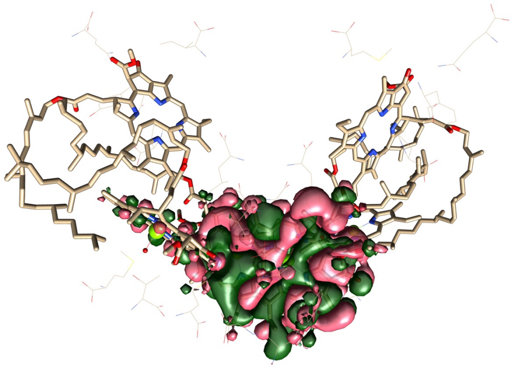

Virtual orbital: 2326

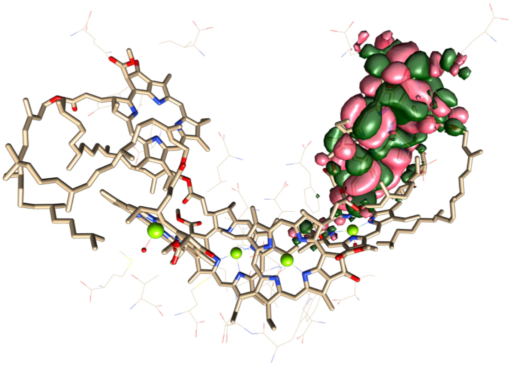

Figure S4 c) (page 4): Molecular Orbital Surfaces for Model 4 (23 amino acids, full phytol chains) wb97xd/6-31G(d, p) with iso-surface value +/- 0.002

H198A: wB97x-D/6-31Gdp (23 amino acids, full phytol chains)

Table S5 a) State Energies and Oscillator Strengths for H198A (23 amino acids, full phytol chains) wb97xd/6-31G(d,p)

State	Energy/ nm	Oscillator Strength
**1**	692.27	0.3681
**2**	687.13	0.4074
**3**	671.81	0.0499
**4**	665.33	0.2393
**5**	659.91	0.4322
**6**	654.85	0.1167
**7**	584.35	0.0676
**8**	579.21	0.0815
**9**	576.51	0.0651
**10**	565.51	0.1046
**11**	561.12	0.0299
**12**	559.59	0.0768
**13**	456.51	0.0636
**14**	430.1	0.0743
**15**	416.84	0.0723
**16**	405	0.1661
**17**	401.35	0.1063
**18**	399.43	0.2949
**19**	397.29	0.0026
**20**	395.68	1.2042
**21**	393.8	0.9662
**22**	392.44	0.1772

Table S5 b) Transitions and Transition Contributions for H198A (23 amino acids, full phytol chains) wb97xd/6-31G(d,p)

State	Occupied MO	Virtual MO	Transition	Corrected transition %
1	2290	2301	-0.24205	11.7
1	2290	2308	0.22834	10.4
1	2296	2301	0.57027	65.0
1	2296	2308	0.12866	3.3
1	2300	2305	0.12273	3.0
2	2293	2305	-0.2877	16.6
2	2293	2311	-0.22712	10.3
2	2296	2301	-0.13022	3.4
2	2300	2305	0.54008	58.3
2	2300	2311	-0.16495	5.4
3	2291	2304	-0.13226	3.5

3	2291	2309	0.17097	5.8
3	2294	2304	0.181	6.6
3	2294	2309	0.10437	2.2
3	2295	2302	-0.10368	2.1
3	2297	2303	0.29249	17.1
3	2298	2304	0.48538	47.1
3	2299	2304	-0.1214	2.9
4	2294	2306	-0.14921	4.5
4	2294	2312	-0.14811	4.4
4	2295	2302	0.25367	12.9
4	2297	2303	-0.13047	3.4
4	2298	2304	0.2118	9.0
4	2298	2306	0.12704	3.2
4	2299	2306	0.47985	46.1
5	2289	2307	-0.12139	2.9
5	2292	2303	-0.13281	3.5
5	2292	2310	0.1933	7.5
5	2295	2302	0.30821	19.0
5	2297	2303	0.49083	48.2
5	2298	2304	-0.16256	5.3
6	2289	2302	-0.12945	3.4
6	2289	2307	-0.1909	7.3
6	2294	2312	0.10558	2.2
6	2295	2302	0.45865	42.1
6	2297	2303	-0.19435	7.6
6	2298	2304	0.11622	2.7
6	2298	2306	-0.11746	2.8
6	2299	2306	-0.31572	19.9
7	2291	2306	-0.16648	5.5
7	2294	2306	0.45557	41.5
7	2298	2306	-0.319	20.4
7	2299	2306	0.24688	12.2
7	2299	2312	0.23819	11.3
8	2290	2301	0.55719	62.1
8	2292	2301	0.11188	2.5
8	2296	2301	0.2836	16.1
8	2296	2308	-0.24956	12.5
8	2296	2313	-0.11404	2.6
9	2293	2305	0.52674	55.5
9	2300	2305	0.33825	22.9
9	2300	2311	0.25848	13.4
9	2300	2314	-0.10153	2.1

10	2292	2303	-0.59011	69.6
10	2297	2303	-0.17293	6.0
10	2297	2310	0.26526	14.1
11	2289	2302	0.28815	16.6
11	2291	2304	0.45307	41.1
11	2294	2304	0.22136	9.8
11	2295	2307	0.14134	4.0
11	2298	2309	-0.22942	10.5
11	2299	2304	0.16469	5.4
12	2289	2302	0.51419	52.9
12	2291	2304	-0.24886	12.4
12	2294	2304	-0.13011	3.4
12	2295	2302	0.16598	5.5
12	2295	2307	0.26079	13.6
12	2298	2309	0.12189	3.0
13	2294	2304	-0.32885	21.6
13	2294	2309	0.13353	3.6
13	2298	2304	0.24263	11.8
13	2299	2304	0.4513	40.7
13	2299	2309	-0.19877	7.9
14	2294	2304	0.36786	27.1
14	2298	2304	-0.12393	3.1
14	2298	2306	-0.15843	5.0
14	2298	2309	0.35793	25.6
14	2298	2312	-0.10069	2.0
14	2299	2304	0.26005	13.5
14	2299	2309	-0.16742	5.6
14	2299	2312	-0.19449	7.6
15	2291	2304	-0.11859	2.8
15	2291	2306	-0.33661	22.7
15	2294	2306	0.20787	8.6
15	2298	2306	0.43518	37.9
15	2298	2312	0.17792	6.3
15	2299	2304	0.13172	3.5
15	2299	2306	-0.17108	5.9
16	2291	2304	-0.27605	15.2
16	2291	2306	0.17191	5.9
16	2291	2309	0.20685	8.6
16	2294	2304	0.11242	2.5
16	2294	2306	-0.1008	2.0
16	2294	2309	-0.16096	5.2
16	2298	2304	-0.10872	2.4

16	2298	2309	-0.24721	12.2
16	2299	2304	0.25543	13.0
16	2299	2312	0.25105	12.6
17	2289	2302	-0.13628	3.7
17	2290	2301	0.11735	2.8
17	2292	2303	-0.12151	3.0
17	2293	2305	0.10361	2.1
17	2295	2307	0.28749	16.5
17	2296	2308	0.32258	20.8
17	2297	2310	-0.29749	17.7
17	2300	2311	-0.24556	12.1
18	2289	2302	0.14083	4.0
18	2290	2301	0.12846	3.3
18	2290	2308	-0.10446	2.2
18	2293	2305	-0.13561	3.7
18	2295	2307	-0.29192	17.0
18	2296	2308	0.35586	25.3
18	2296	2313	-0.11098	2.5
18	2297	2310	-0.17898	6.4
18	2300	2311	0.32369	21.0
19	2299	2302	-0.68401	93.6
19	2299	2307	0.11046	2.4
20	2281	2306	0.13653	3.7
20	2290	2301	-0.10567	2.2
20	2290	2308	0.10512	2.2
20	2291	2306	-0.12153	3.0
20	2291	2309	0.10608	2.3
20	2292	2303	-0.16189	5.2
20	2296	2308	-0.28837	16.6
20	2296	2313	0.12805	3.3
20	2297	2310	-0.36685	26.9
20	2298	2306	-0.10649	2.3
20	2299	2312	-0.11999	2.9
20	2300	2311	0.16993	5.8
21	2289	2302	0.18142	6.6
21	2289	2307	0.11003	2.4
21	2291	2304	-0.10803	2.3
21	2293	2305	0.14653	4.3
21	2293	2311	0.11852	2.8
21	2295	2307	-0.36464	26.6
21	2298	2309	-0.13286	3.5
21	2299	2312	-0.13975	3.9

21	2300	2311	-0.33883	23.0
21	2300	2314	-0.14263	4.1
22	2281	2306	0.22025	9.7
22	2282	2306	0.16026	5.1
22	2291	2304	-0.14518	4.2
22	2291	2306	-0.1562	4.9
22	2292	2303	0.1083	2.3
22	2294	2309	-0.11343	2.6
22	2295	2307	0.12029	2.9
22	2296	2308	0.12194	3.0
22	2297	2310	0.24025	11.5
22	2298	2306	-0.12477	3.1
22	2298	2309	-0.23711	11.2
22	2299	2312	-0.26856	14.4
22	2300	2311	0.11796	2.8

Table S5 c) Cofactor % Contributions* to Molecular Orbitals for H198A (23 amino acids, full phytol chains) wb97xd/6-31G(d, p)

MO	Phe $_{D1}$	Phe $_{D2}$	P $_{D1}$	P $_{D2}$	Chl $_{D1}$	Chl $_{D2}$
2281	0.0	0.0	43.8	23.3	0.0	0.2
2282	0.0	0.1	20.5	11.8	0.0	0.1
2289	0.0	0.3	0.0	0.2	0.0	99.0
2290	96.5	0.0	0.7	0.0	2.4	0.0
2291	0.0	0.0	84.8	13.7	0.6	0.0
2292	3.0	0.0	0.3	0.4	95.4	0.0
2293	0.0	97.6	0.0	0.3	0.0	1.7
2294	0.0	0.1	32.8	65.2	0.3	0.4
2295	0.0	1.7	0.0	0.7	0.0	97.3
2296	98.3	0.0	0.4	0.0	0.9	0.0
2297	0.4	0.0	0.4	0.7	98.1	0.0
2298	0.0	0.0	72.9	25.8	0.3	0.1
2299	0.0	0.0	7.5	91.3	0.4	0.0
2300	0.0	99.1	0.0	0.2	0.0	0.1
2301	98.2	0.0	0.4	0.0	0.8	0.0
2302	0.0	1.1	0.0	0.2	0.0	98.3
2303	0.9	0.0	1.3	0.4	96.8	0.0
2304	0.0	0.0	95.9	1.9	1.2	0.0
2305	0.0	98.4	0.0	0.3	0.0	1.0
2306	0.0	0.0	1.7	97.0	0.2	0.1
2307	0.0	0.5	0.4	0.3	0.0	98.4
2308	97.9	0.0	0.5	0.0	1.2	0.0
2309	0.3	0.0	92.0	3.6	2.5	0.4

2310	1.2	0.0	2.4	1.0	94.6	0.0
2311	0.0	98.9	0.0	0.7	0.0	0.2
2312	0.0	0.1	4.8	93.4	0.6	0.2
2313	98.0	0.0	0.2	0.0	0.3	0.0
2314	0.0	98.6	0.0	0.2	0.0	0.2

* as determined by the Mulliken Charges method in MultiWfn

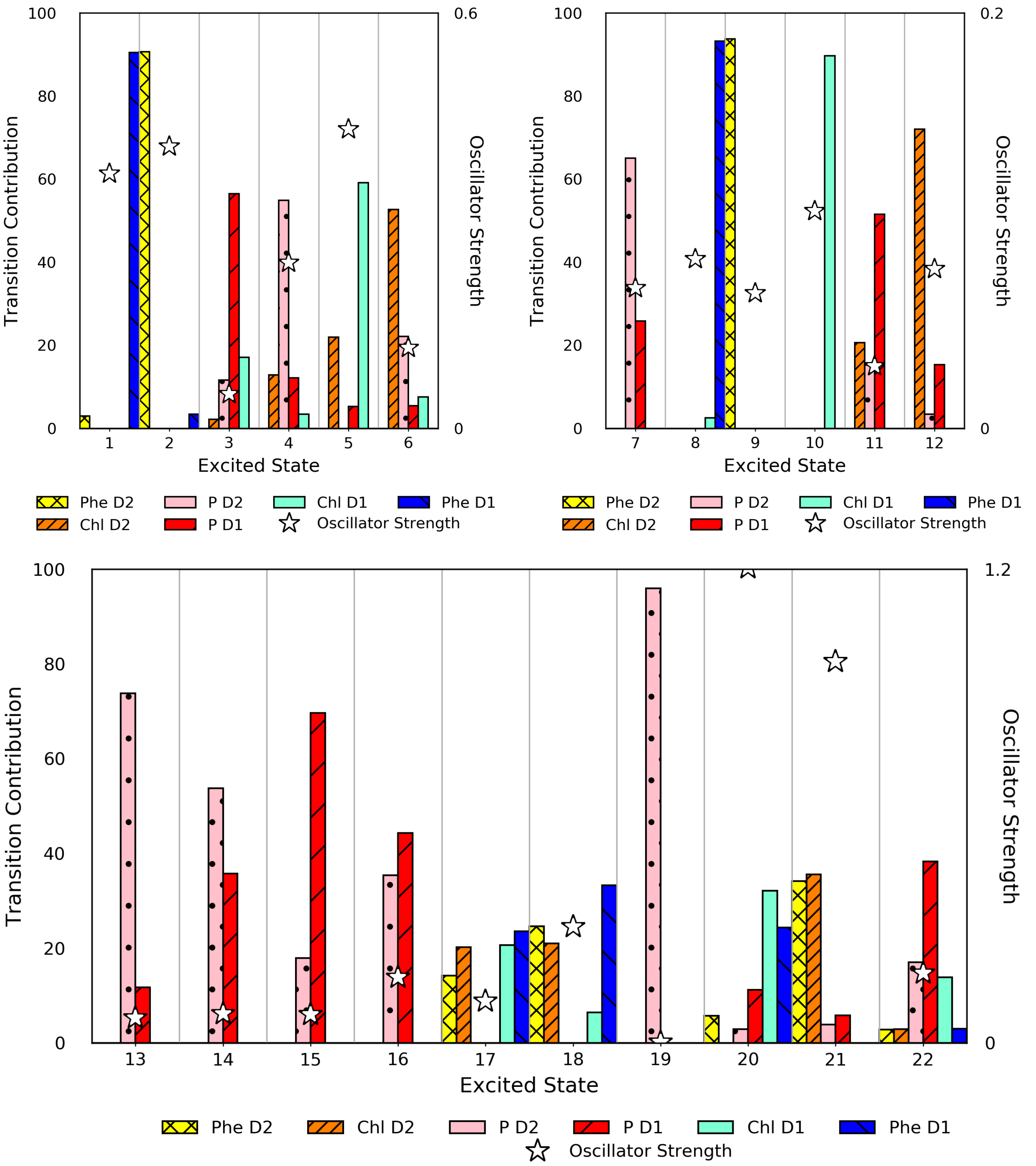

Figure S5 a) Showing the oscillator strengths (white stars) and % contribution of transitions from each cofactor excitation for Model 4 H198A (23 amino acids and full phytol chains) : (top left) states 1-6, (top right) 7-12 and (bottom) states 13-22

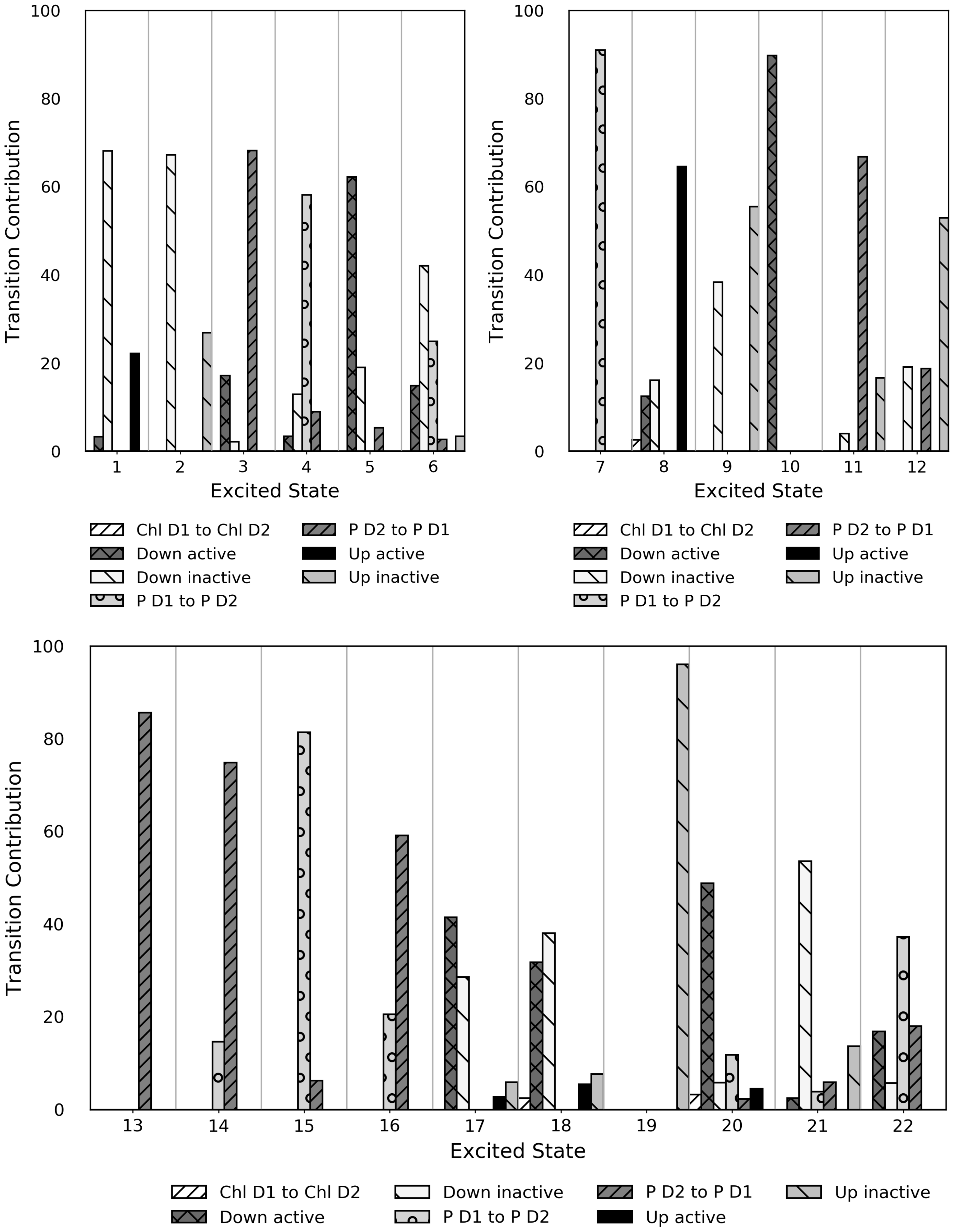

Figure S5 b) Showing % contribution of each type of transition for Model 4 H198A (23 amino acids and full phytol chains) : (top left) states 1-6, (top right) 7-12 and (bottom) states 13-22.

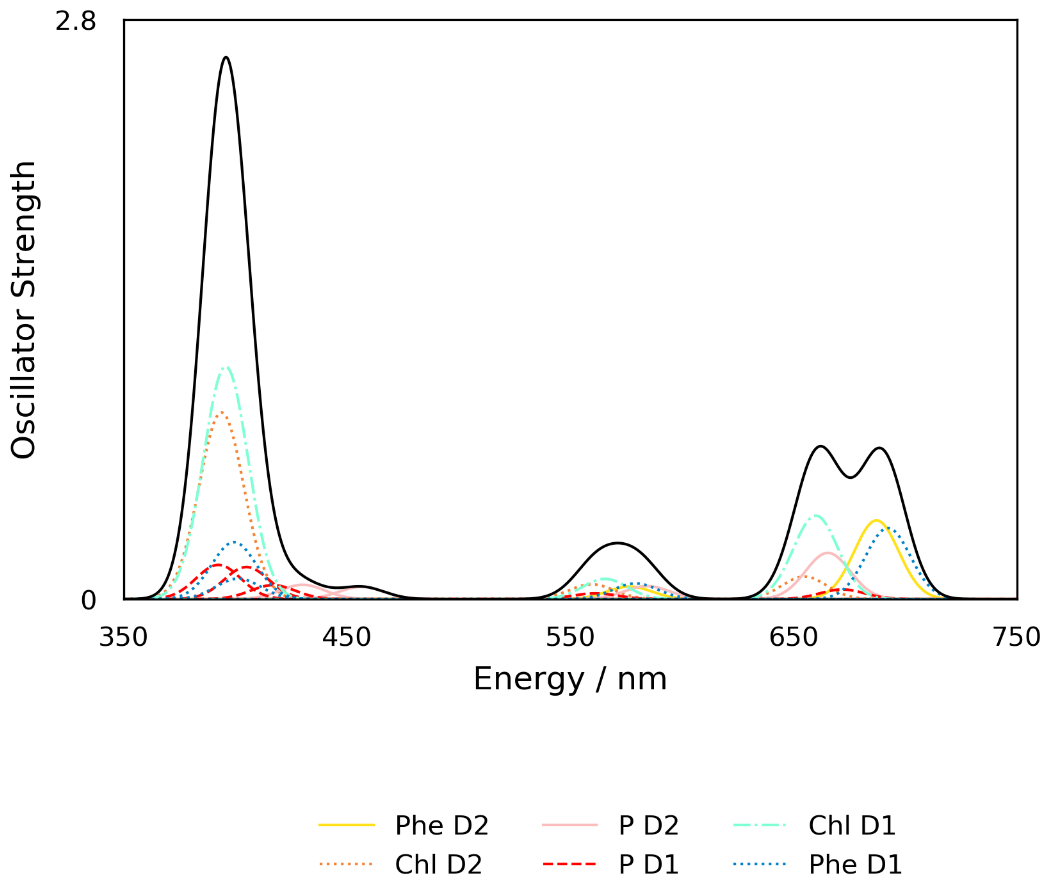

Figure S5 c) Simulated Absorption spectrum for model 4 H198A (23 amino acids, full phytol chains) : Black solid spectrum – overall calculated absorption spectrum, Colored lines – single excited states are colored according to the cofactor of the dominant transition as in table 1 and figure 2: Phe_{D1} – Blue, Chl_{D1} – Aquamarine, P_{D1} – Red, P_{D2} – Pink, Chl_{D2} – Orange and Phe_{D2} – Orange.

His-197-Ala: wB97x-D/6-31Gdp (23 amino acids, full phytol chains)

Table S6 a) State Energies and Oscillator Strengths for His-197-Ala (23 amino acids, full phytol chains) wb97xd/6-31G(d,p)

State	Energy/ nm	Oscillator Strength
**1**	692.27	0.3703
**2**	687.16	0.4181
**3**	675.49	0.0949
**4**	665.27	0.1796
**5**	660.04	0.4057
**6**	654.69	0.1405
**7**	579.36	0.0725
**8**	577.93	0.1128
**9**	576.24	0.0296
**10**	570.92	0.0376
**11**	564.75	0.1021
**12**	561.09	0.0713
**13**	446.14	0.0162
**14**	431.1	0.0495
**15**	423.59	0.2646
**16**	411.07	0.141
**17**	401.18	0.1081
**18**	399.56	0.2612
**19**	398.81	0.0095
**20**	395.24	1.5715
**21**	393.79	0.2008
**22**	393.1	0.0361

Table S6 b) Transitions and Transition Contributions for His-197-Ala (23 amino acids, full phytol chains) wb97xd/6-31G(d,p)

State	Occupied MO	Virtual MO	Transition	Corrected transition %
1	2290	2301	-0.24169	11.7
1	2290	2308	0.22982	10.6
1	2296	2301	0.57006	65.0
1	2296	2308	0.12796	3.3
1	2299	2305	0.12427	3.1
2	2293	2305	-0.27581	15.2
2	2293	2311	0.11559	2.7
2	2293	2312	-0.18553	6.9
2	2296	2301	-0.13558	3.7
2	2299	2305	0.53006	56.2
2	2299	2312	-0.13903	3.9
3	2291	2306	0.13359	3.6

3	2294	2309	0.15015	4.5
3	2297	2304	-0.12647	3.2
3	2298	2303	-0.12809	3.3
3	2300	2306	0.58263	67.9
4	2289	2307	-0.11416	2.6
4	2291	2309	-0.10859	2.4
4	2294	2303	-0.15832	5.0
4	2294	2304	-0.1597	5.1
4	2295	2302	0.29577	17.5
4	2297	2304	0.3164	20.0
4	2298	2303	0.32549	21.2
4	2298	2304	0.13781	3.8
4	2300	2306	0.13543	3.7
5	2289	2302	-0.10085	2.0
5	2289	2307	-0.13306	3.5
5	2292	2310	0.18248	6.7
5	2295	2302	0.31963	20.4
5	2297	2303	0.30533	18.6
5	2297	2304	-0.22177	9.8
5	2298	2303	-0.21527	9.3
5	2298	2304	0.25202	12.7
5	2300	2306	-0.1185	2.8
6	2289	2302	-0.11708	2.7
6	2289	2307	-0.17101	5.8
6	2293	2302	0.10264	2.1
6	2294	2303	0.12198	3.0
6	2294	2304	0.14279	4.1
6	2295	2302	0.38709	30.0
6	2297	2303	-0.28123	15.8
6	2298	2304	-0.29482	17.4
6	2300	2306	0.10375	2.2
7	2290	2301	0.55608	61.8
7	2296	2301	0.28005	15.7
7	2296	2308	-0.24765	12.3
7	2296	2313	-0.11301	2.6
8	2291	2303	-0.2938	17.3
8	2291	2304	-0.31691	20.1
8	2293	2305	-0.22573	10.2
8	2294	2303	-0.14635	4.3
8	2294	2304	-0.16357	5.4
8	2298	2311	-0.10117	2.0
8	2299	2305	-0.14541	4.2

8	2300	2303	0.14393	4.1
8	2300	2304	0.15733	5.0
9	2291	2303	-0.14281	4.1
9	2291	2304	-0.15427	4.8
9	2293	2305	0.4558	41.6
9	2295	2305	-0.12043	2.9
9	2299	2305	0.30377	18.5
9	2299	2311	-0.1231	3.0
9	2299	2312	0.19779	7.8
10	2291	2306	-0.22637	10.2
10	2294	2306	0.44291	39.2
10	2295	2306	0.10385	2.2
10	2297	2306	0.19365	7.5
10	2298	2306	0.26333	13.9
10	2300	2306	0.12092	2.9
10	2300	2309	-0.23261	10.8
10	2300	2311	0.14199	4.0
11	2292	2303	-0.44139	39.0
11	2292	2304	0.39201	30.7
11	2297	2304	0.10121	2.0
11	2297	2310	0.18641	6.9
11	2298	2303	0.10117	2.0
11	2298	2310	-0.17195	5.9
12	2289	2302	-0.59125	69.9
12	2295	2302	-0.17593	6.2
12	2295	2307	-0.27568	15.2
13	2291	2303	0.19163	7.3
13	2291	2304	0.20618	8.5
13	2300	2303	0.36662	26.9
13	2300	2304	0.39221	30.8
13	2300	2311	0.11759	2.8
14	2282	2306	-0.12549	3.1
14	2291	2303	0.12902	3.3
14	2291	2304	0.14381	4.1
14	2291	2306	-0.16149	5.2
14	2291	2309	0.13509	3.6
14	2294	2306	-0.25709	13.2
14	2297	2306	0.26404	13.9
14	2297	2309	-0.15888	5.0
14	2298	2306	0.32367	21.0
14	2298	2309	-0.20007	8.0
14	2300	2309	0.17519	6.1

15	2282	2306	-0.14335	4.1
15	2291	2306	-0.1538	4.7
15	2294	2306	-0.33652	22.6
15	2300	2309	-0.41394	34.3
15	2300	2311	0.17761	6.3
15	2300	2312	0.10749	2.3
16	2282	2303	-0.10124	2.0
16	2282	2304	-0.1145	2.6
16	2291	2303	-0.11954	2.9
16	2291	2304	-0.13371	3.6
16	2294	2303	0.28588	16.3
16	2294	2304	0.30103	18.1
16	2294	2311	0.13771	3.8
16	2298	2303	0.13695	3.8
16	2298	2304	0.14853	4.4
16	2300	2303	0.21022	8.8
16	2300	2304	0.18073	6.5
16	2300	2309	0.16858	5.7
17	2289	2302	0.14857	4.4
17	2290	2301	-0.11168	2.5
17	2293	2305	-0.10476	2.2
17	2295	2307	-0.30282	18.3
17	2296	2308	-0.30352	18.4
17	2297	2310	0.19554	7.6
17	2298	2310	-0.15693	4.9
17	2299	2311	-0.13931	3.9
17	2299	2312	0.22304	9.9
18	2289	2302	-0.12655	3.2
18	2290	2301	-0.14226	4.0
18	2290	2308	0.11602	2.7
18	2293	2305	0.10528	2.2
18	2295	2307	0.25473	13.0
18	2296	2308	-0.39271	30.8
18	2296	2313	0.12201	3.0
18	2297	2310	0.13592	3.7
18	2298	2310	-0.1093	2.4
18	2299	2311	0.14065	4.0
18	2299	2312	-0.22528	10.2
19	2297	2302	0.33989	23.1
19	2298	2302	0.40346	32.6
19	2300	2302	-0.40508	32.8
20	2291	2309	-0.1056	2.2

20	2292	2303	-0.11349	2.6
20	2292	2304	0.10948	2.4
20	2293	2305	-0.10982	2.4
20	2296	2308	-0.25087	12.6
20	2296	2313	0.11679	2.7
20	2297	2310	-0.26399	13.9
20	2298	2306	0.10468	2.2
20	2298	2310	0.21063	8.9
20	2299	2311	-0.1423	4.0
20	2299	2312	0.22823	10.4
20	2299	2314	-0.1022	2.1
21	2289	2302	-0.1599	5.1
21	2289	2307	-0.12351	3.1
21	2293	2305	-0.12803	3.3
21	2295	2307	0.31024	19.2
21	2296	2308	0.12627	3.2
21	2297	2310	0.18362	6.7
21	2298	2310	-0.19506	7.6
21	2299	2311	-0.15824	5.0
21	2299	2312	0.25375	12.9
21	2299	2314	-0.11863	2.8
21	2300	2303	-0.10472	2.2
22	2300	2303	0.47105	44.4
22	2300	2304	-0.45556	41.5

Table S6 c) Cofactor % Contributions* to Molecular Orbitals for His-197-Ala (23 amino acids, full phytol chains) wb97xd/6-31G(d, p)

	Phe D1	Phe D2	P D1	P D2	Chl D1	Chl D2
2282	0.0	0.0	76.8	20.7	0.0	0.4
2289	0.0	0.3	0.0	0.2	0.0	99.0
2290	97.2	0.0	0.7	0.0	1.7	0.0
2291	0.0	0.0	25.7	72.4	0.7	0.1
2292	2.5	0.0	0.9	0.3	95.4	0.0
2293	0.0	92.3	0.0	0.3	0.0	7.1
2294	0.0	0.5	55.1	37.6	0.6	5.4
2295	0.0	6.6	3.1	3.1	0.0	86.9
2296	98.1	0.0	0.4	0.0	1.1	0.0
2297	0.2	0.0	7.9	34.9	56.5	0.0
2298	0.2	0.0	13.8	43.2	42.4	0.0
2299	0.0	99.1	0.0	0.2	0.0	0.1
2300	0.0	0.0	92.6	6.3	0.1	0.1
2301	98.4	0.0	0.4	0.0	0.6	0.0
2302	0.0	0.7	0.0	0.2	0.0	98.7
2303	0.4	0.1	1.4	43.6	53.8	0.0
2304	0.3	2.2	0.9	52.3	43.6	0.0
2305	0.0	96.5	0.0	1.9	0.6	0.6
2306	0.0	0.0	96.2	2.6	0.2	0.0
2307	0.0	0.5	0.1	0.4	0.0	98.8
2308	98.5	0.0	0.4	0.0	0.7	0.0
2309	0.0	0.0	67.0	30.4	1.5	0.1
2310	0.8	0.0	1.8	12.7	83.9	0.0
2311	0.1	27.6	22.8	39.1	9.5	0.2
2312	0.0	71.4	8.5	16.2	3.2	0.2
2313	98.0	0.0	0.2	0.0	0.2	0.0

* as determined by the Mulliken Charges method in MultiWfn

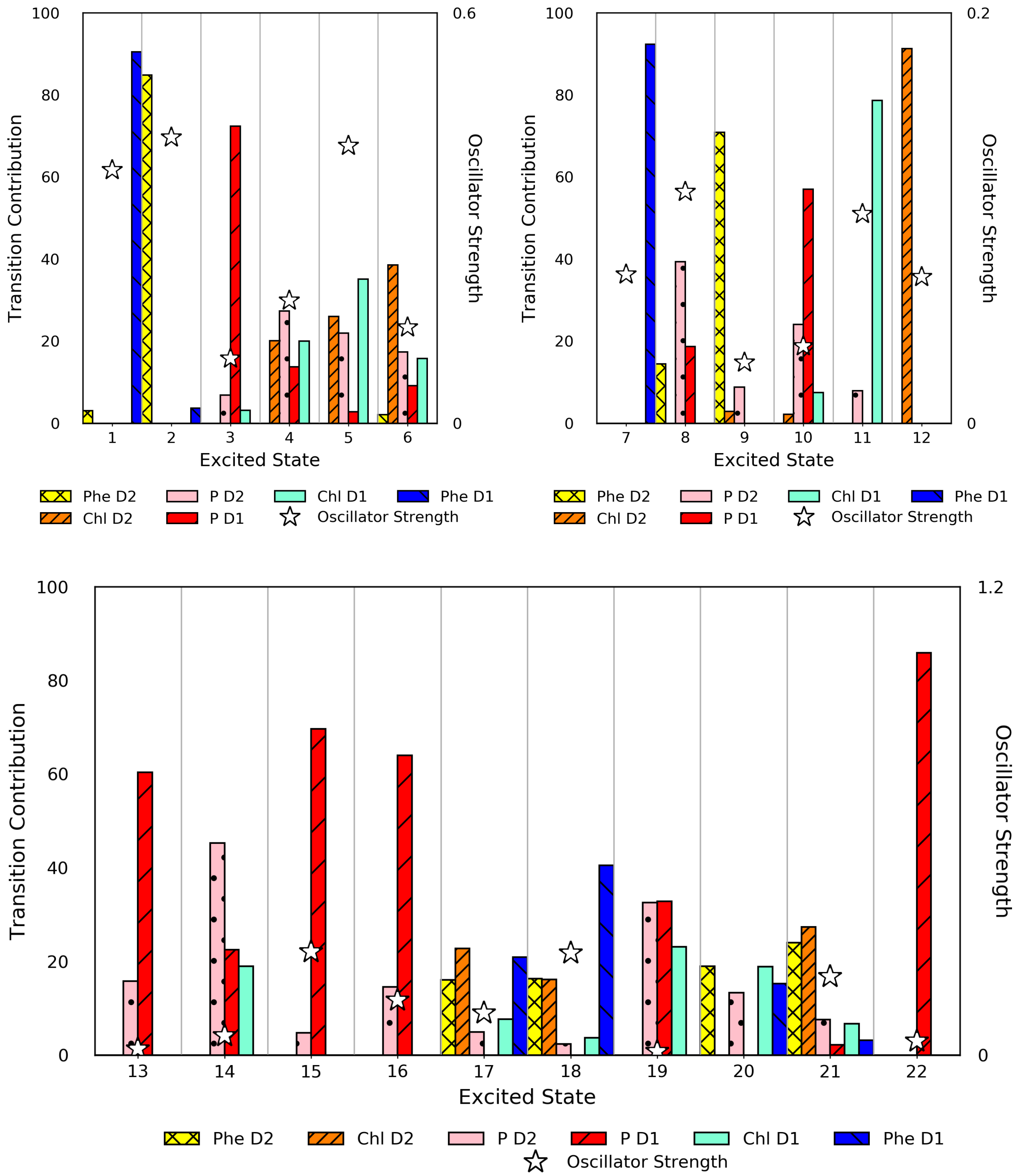

Figure S6 a) Showing the oscillator strengths (white stars) and % contribution of transitions from each cofactor excitation for model 4 H197A (23 amino acids and full phytol chains) : (top left) states 1-6, (top right) 7-12 and (bottom) states 13-22

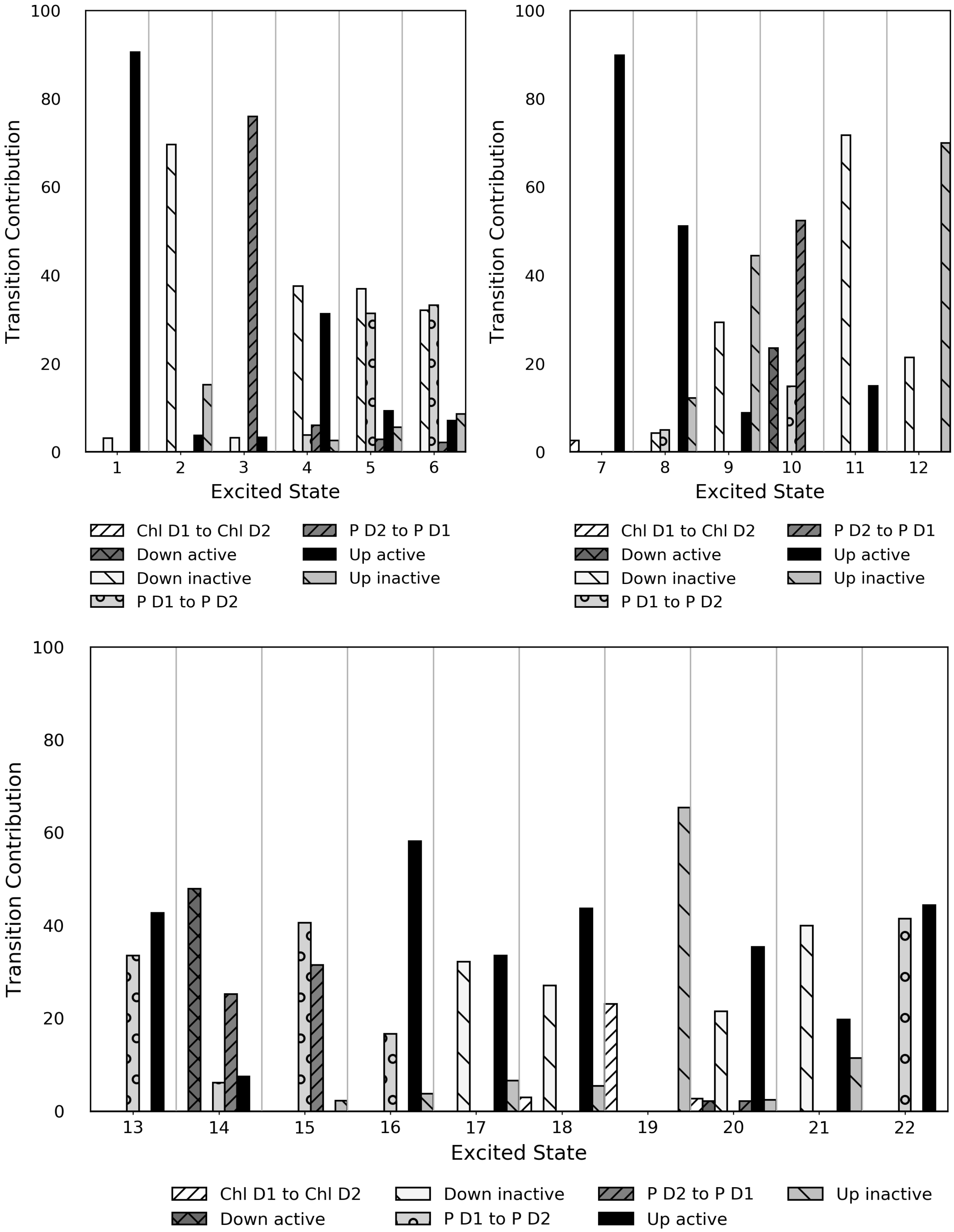

Figure S6 b) Showing % contribution of each type of transition for model 4 H197A (with 23 amino acids and full phytol chains): (top left) states 1-6, (top right) 7-12 and (bottom) states 13-22.

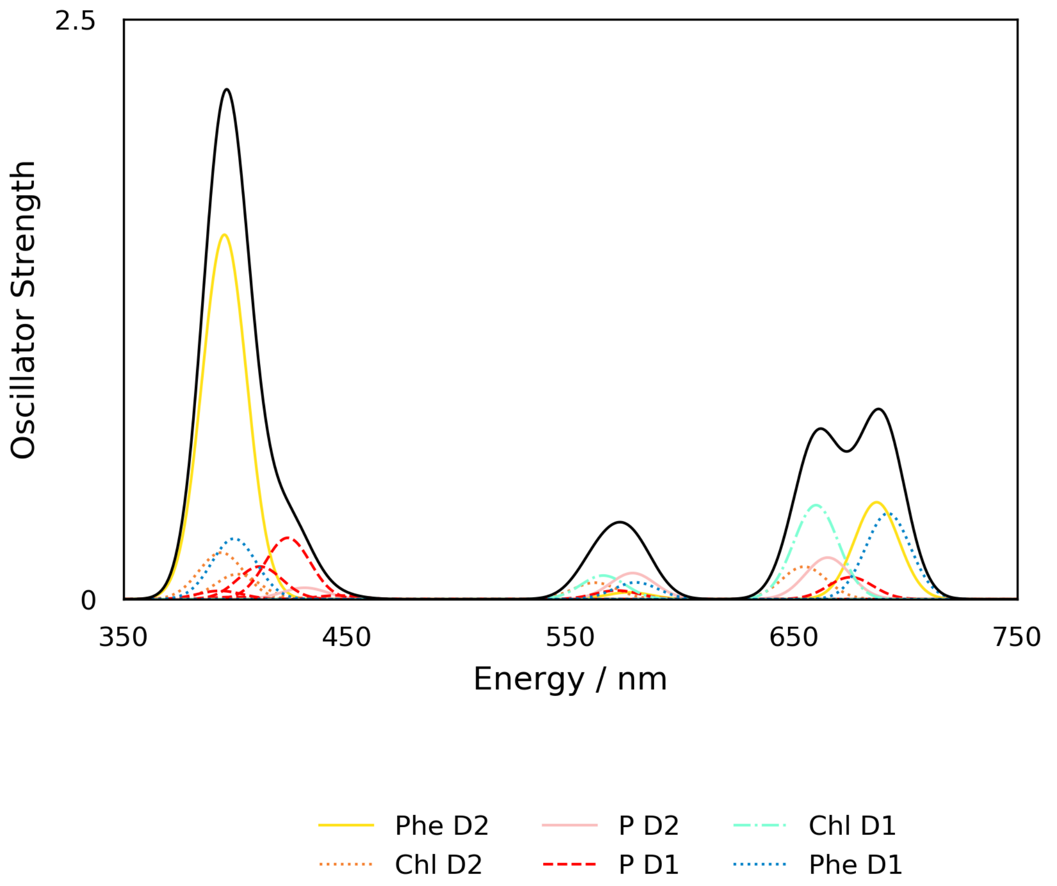

Figure S6 c) Simulated Absorption spectrum for model 4 H197A (23 amino acids, full phytol chains) : Black solid spectrum – overall calculated absorption spectrum, Colored lines – single excited states are colored according to the cofactor of the dominant transition as in table 1 and figure 2: Phe_{D1} – Blue, Chl_{D1} – Aquamarine, P_{D1} – Red, P_{D2} – Pink, Chl_{D2} – Orange and Phe_{D2} – Orange.

Additional Methods TD-DFT Results
Diffuse Basis Function - Model 1 (2 amino acids, truncated phytol chains) wB97x-D/6-31+Gdp

Table S7 a) State Energies and Oscillator Strengths for Model 1 (2 amino acids, truncated phytol chains) wb97xd/6-31+G(d, p)

State	Energy/ nm	Oscillator Strength
1	684.33	0.435
2	680.06	0.1199
3	678.72	0.4169
4	667.88	0.0535
5	663.12	0.375
6	657.15	0.1573

Table S7 b) Transitions and Transition Contributions for Model 1 (2 amino acids, truncated phytol chains) wb97xd/6-31+G(d, p)

State	Occupied MO	Virtual MO	Transition Contribution	Transition %
1	1136	1148	-0.24974	12.4740135
1	1136	1154	-0.20849	8.69361602
1	1139	1146	-0.10879	2.36705282
1	1140	1147	0.10095	2.0381805
1	1142	1148	0.46158	42.6112193
1	1142	1154	-0.15041	4.52463362
1	1144	1149	-0.21097	8.90166818
1	1144	1150	0.20035	8.0280245
2	1134	1147	-0.1078	2.324168
2	1135	1147	-0.11753	2.76266018
2	1135	1151	-0.13529	3.66067682
2	1137	1145	-0.14398	4.14604808
2	1138	1145	0.25176	12.6766195
2	1140	1145	0.12096	2.92626432
2	1140	1147	0.32575	21.2226125
2	1142	1148	0.12304	3.02776832
2	1144	1149	0.25289	12.7906704
2	1144	1150	-0.25406	12.9092967
3	1134	1147	0.10088	2.03535488
3	1135	1147	0.12839	3.29679842
3	1135	1151	0.15924	5.07147552
3	1136	1148	-0.13592	3.69484928
3	1136	1154	-0.11631	2.70560322
3	1140	1145	-0.11632	2.70606848
3	1140	1147	-0.36711	26.9539504
3	1142	1148	0.2469	12.191922
3	1144	1149	0.25642	13.1502433

3	1144	1150	-0.23728	11.2603597
4	1133	1152	0.14812	4.38790688
4	1138	1145	0.11519	2.65374722
4	1139	1146	0.38871	30.2190928
4	1140	1147	-0.1278	3.266568
4	1141	1150	0.11818	2.79330248
4	1143	1149	-0.28879	16.6799328
4	1143	1150	-0.30254	18.3060903
4	1144	1149	-0.10214	2.08651592
5	1133	1152	-0.10475	2.1945125
5	1134	1145	0.1013	2.052338
5	1134	1153	-0.18317	6.71024978
5	1135	1153	0.1012	2.048288
5	1137	1145	-0.24474	11.9795335
5	1138	1145	0.43027	37.0264546
5	1138	1147	-0.11652	2.71538208
5	1139	1146	-0.25808	13.3210573
5	1140	1147	-0.14689	4.31533442
5	1144	1149	-0.12792	3.27270528
5	1144	1150	0.12585	3.1676445
6	1133	1152	0.17049	5.81336802
6	1137	1156	0.10393	2.16028898
6	1138	1145	0.13918	3.87421448
6	1139	1146	0.39429	31.0929208
6	1141	1149	-0.10233	2.09428578
6	1141	1150	-0.11726	2.74998152
6	1143	1149	0.30293	18.353317
6	1143	1150	0.28223	15.9307546
6	1144	1150	0.10847	2.35314818

Table S7 c) Cofactor % Contributions* to Molecular Orbitals for Model 1 (2 amino acids, truncated phytol chains) wb97xd/6-31+G(d, p)

	Phe D1	Phe D2	P D1	P D2	Chl D1	Chl D2
1133	0.0	0.9	0.0	0.2	0.0	98.9
1134	26.1	0.0	0.0	0.2	73.6	0.0
1135	74.1	0.0	0.0	0.0	25.8	0.0
1136	0.0	98.8	0.0	-0.1	0.0	1.2
1137	0.2	0.0	31.5	42.4	25.1	0.2
1138	0.5	0.0	9.1	15.6	73.3	1.2
1139	0.0	0.7	0.5	0.8	0.5	97.5
1140	94.4	0.0	3.0	2.4	0.3	0.0
1141	5.3	0.0	50.6	43.6	0.2	-0.1
1142	0.0	100.2	0.0	0.0	0.0	-0.2

1143	0.0	0.0	15.0	85.0	0.1	-0.3
1144	0.0	0.0	90.3	9.5	-0.1	-0.1
1145	8.5	0.0	-0.2	0.1	91.6	0.0
1146	0.0	2.6	-0.1	0.2	0.0	97.4
1147	92.1	0.0	0.0	0.0	7.9	0.0
1148	0.0	97.8	0.0	-0.1	0.0	2.2
1149	0.0	0.0	52.1	48.6	-0.3	0.0
1150	0.0	0.0	47.1	53.5	-0.2	-0.1
1151	98.5	0.0	-0.1	0.0	1.6	0.0
1152	0.0	0.1	0.1	0.3	0.0	99.3
1153	2.1	0.0	0.2	0.5	97.2	0.0
1154	0.0	99.8	0.0	-0.1	0.0	0.2
1156	0.0	0.0	42.5	57.2	0.4	0.0

* as determined by the Mulliken Charges method in MultiWfn

Alternative Functional CAM-B3LYP - Model 1 (2 amino acids, truncated phytol chains) CAM-B3LYP/6-31Gdp

Table S8 a) State Energies and Oscillator Strengths for Model 1 (2 amino acids, truncated phytol chains) CAM-B3LYP/6-31G(d, p)

State	Energy/ nm	Oscillator Strength
**1**	657.67	0.4463
**2**	654.06	0.0563
**3**	652.39	0.5234
**4**	643.95	0.0533
**5**	638.58	0.3514
**6**	634.50	0.1505

Table S8 b) Transitions and Transition Contributions for Model 1 (2 amino acids, truncated phytol chains) CAM-B3LYP/6-31G(d, p)

State	Occupied MO	Virtual MO	Transition Contribution	Transition %
1	1136	1148	-0.20969	8.79397922
1	1136	1154	-0.1609	5.177762
1	1142	1148	0.36754	27.0171303
1	1142	1154	-0.12489	3.11950242
1	1144	1149	0.30767	18.9321658
1	1144	1150	0.30104	18.1250163
2	1136	1148	-0.16644	5.54045472
2	1136	1154	-0.13027	3.39405458
2	1137	1146	-0.12924	3.34059552
2	1138	1146	0.14138	3.99766088
2	1139	1146	-0.17432	6.07749248
2	1140	1147	0.2387	11.395538
2	1142	1148	0.29064	16.8943219

2	1142	1154	-0.10345	2.1403805
2	1144	1149	-0.24575	12.0786125
2	1144	1150	-0.24891	12.3912376
3	1134	1147	0.12699	3.22529202
3	1134	1152	-0.10179	2.07224082
3	1135	1147	-0.15795	4.9896405
3	1135	1152	0.16971	5.76029682
3	1136	1148	0.14785	4.3719245
3	1136	1154	0.11497	2.64362018
3	1140	1146	-0.13281	3.52769922
3	1140	1147	0.41192	33.9356173
3	1140	1152	0.11882	2.82363848
3	1141	1147	-0.10566	2.23280712
3	1142	1148	-0.25012	12.5120029
3	1144	1149	0.1566	4.904712
3	1144	1150	0.14333	4.10869778
4	1133	1151	-0.12936	3.34680192
4	1138	1145	0.28216	15.9228531
4	1139	1145	0.21339	9.10705842
4	1140	1147	-0.1539	4.737042
4	1141	1149	-0.10247	2.10002018
4	1141	1150	0.12425	3.0876125
4	1143	1149	0.32001	20.48128
4	1143	1150	-0.32078	20.5799617
5	1134	1146	-0.10522	2.21424968
5	1134	1153	0.18103	6.55437218
5	1135	1153	0.10562	2.23111688
5	1137	1146	0.25407	12.910313
5	1138	1145	0.19798	7.83921608
5	1138	1146	-0.27508	15.1338013
5	1139	1145	0.14085	3.9677445
5	1139	1146	0.34437	23.7181394
5	1140	1147	0.156	4.8672
5	1144	1149	-0.11761	2.76642242
5	1144	1150	-0.11731	2.75232722
6	1133	1145	0.10613	2.25271538
6	1133	1151	0.1837	6.749138
6	1138	1145	-0.35066	24.5924871
6	1139	1145	-0.2659	14.140562
6	1139	1146	0.11261	2.53620242
6	1141	1150	0.10591	2.24338562
6	1143	1149	0.27332	14.9407645
6	1143	1150	-0.25112	12.6122509

6	1143	1149	0.27332	14.9407645
6	1143	1150	-0.25112	12.6122509

Table S8 c) Cofactor % Contributions* to Molecular Orbitals for Model 1 (2 amino acids, truncated phytol chains) CAM-B3LYP/6-31G(d, p)

	Phe D1	Phe D2	P D1	P D2	Chl D1	Chl D2
1133	0.0	0.9	0.0	0.2	0.0	98.9
1134	28.6	0.2	0.2	0.3	70.6	0.0
1135	70.8	0.3	0.2	0.1	28.6	0.0
1136	0.0	98.4	0.0	0.1	0.2	1.3
1137	0.1	0.0	34.3	38.5	26.2	0.5
1138	0.1	0.4	2.7	4.4	29.2	63.2
1139	0.2	0.2	8.5	11.5	44.1	35.5
1140	93.1	0.0	3.3	3.0	0.5	0.0
1141	6.1	0.0	46.1	46.9	0.3	0.1
1142	0.0	99.7	0.0	0.1	0.0	0.1
1143	0.0	0.0	14.2	85.4	0.1	0.0
1144	0.0	0.0	90.7	8.9	0.1	0.1
1145	0.0	1.8	0.0	0.2	0.0	97.9
1146	7.5	0.1	0.1	0.3	91.9	0.0
1147	91.9	0.3	0.2	0.0	7.6	0.0
1148	0.0	97.9	0.0	0.1	0.1	1.8
1149	0.0	0.0	51.2	48.2	0.1	0.1
1150	0.0	0.0	47.6	51.9	0.1	0.1
1151	0.0	0.2	0.1	0.3	0.0	99.4
1152	97.1	0.0	0.2	0.0	2.7	0.0
1153	2.6	0.0	0.8	0.3	96.3	0.0
1154	0.0	99.3	0.0	0.2	0.1	0.2

* as determined by the Mulliken Charges method in MultiWfn

Model 4 (23 amino acids, full phytol chains) CAM-B3LYP /6-31Gdp

Table S9 a) State Energies and Oscillator Strengths for Model 4 (23 amino acids, full phytol chains) CAM-B3LYP /6-31G(d, p)

State	Energy/ nm	Oscillator Strength
**1**	675.05	0.4194
**2**	669.89	0.4629
**3**	663.44	0.0683
**4**	653.84	0.1888
**5**	647.57	0.3882
**6**	643.13	0.1601
**7**	585.21	0.0621
**8**	576.59	0.0787
**9**	573.08	0.0694
**10**	572.29	0.0276
**11**	563.96	0.1009
**12**	559.03	0.0706
**13**	481.02	0.0003
**14**	479.56	0.0099
**15**	473.7	0.0214
**16**	469.1	0.0001

Table S9 b) Transitions and Transition Contributions for Model 4 (23 amino acids, full phytol chains) CAM-B3LYP /6-31G(d, p)

State	Occupied	Virtual	Transition Contribution	Transition %
1	2302	2313	-0.24203	11.71570418
1	2302	2320	0.2208	9.750528
1	2307	2313	0.56728	64.36131968
1	2307	2320	0.12426	3.08810952
1	2310	2316	0.12457	3.10353698
2	2304	2316	-0.27354	14.96482632
2	2304	2323	-0.20862	8.70446088
2	2307	2313	-0.1365	3.72645
2	2310	2316	0.52213	54.52394738
2	2310	2323	-0.15371	4.72535282
2	2312	2317	0.12128	2.94176768
2	2312	2318	0.12327	3.03909858
3	2305	2317	0.10222	2.08978568
3	2305	2318	0.1043	2.175698
3	2305	2322	-0.10988	2.41472288
3	2308	2322	0.13251	3.51178002
3	2309	2315	0.20257	8.20692098
3	2310	2316	-0.16518	5.45688648

3	2312	2317	0.404	32.6432
3	2312	2318	0.39806	31.69035272
4	2306	2314	0.27604	15.23961632
4	2308	2317	-0.11429	2.61244082
4	2308	2318	0.148	4.3808
4	2308	2324	-0.12907	3.33181298
4	2309	2315	-0.17197	5.91473618
4	2311	2317	0.3703	27.424418
4	2311	2318	-0.33664	22.66529792
5	2301	2319	-0.10822	2.34231368
5	2303	2315	-0.13634	3.71771912
5	2303	2321	0.1856	6.889472
5	2306	2314	0.28288	16.00421888
5	2309	2315	0.52823	55.80538658
6	2301	2314	-0.12876	3.31582752
6	2301	2319	-0.19051	7.25881202
6	2306	2314	0.47177	44.51338658
6	2309	2315	-0.19484	7.59252512
6	2311	2317	-0.24163	11.67701138
6	2311	2318	0.22363	10.00207538
7	2305	2317	0.23997	11.51712018
7	2305	2318	-0.2056	8.454272
7	2308	2317	0.31561	19.92193442
7	2308	2318	-0.27972	15.64865568
7	2311	2318	-0.1713	5.868738
7	2311	2324	0.22533	10.15472178
7	2312	2317	-0.22068	9.73993248
7	2312	2318	0.23347	10.90164818
8	2302	2313	0.55949	62.60581202
8	2303	2313	0.1003	2.012018
8	2307	2313	0.28028	15.71137568
8	2307	2320	-0.24814	12.31469192
8	2307	2325	-0.1112	2.473088
9	2304	2316	0.52072	54.22986368
9	2306	2316	0.10014	2.00560392
9	2310	2316	0.33467	22.40080178
9	2310	2323	0.25923	13.44003858
10	2305	2317	0.24835	12.3355445
10	2305	2318	0.29357	17.23666898
10	2308	2317	-0.2932	17.193248
10	2308	2318	-0.2284	10.433312
10	2311	2317	-0.21776	9.48388352
10	2311	2318	-0.18985	7.2086045

10	2312	2321	-0.11322	2.56375368
10	2312	2322	0.2431	11.819522
11	2303	2315	-0.59666	71.20063112
11	2309	2315	-0.16914	5.72166792
11	2309	2321	0.25302	12.80382408
11	2309	2322	0.10718	2.29751048
12	2301	2314	-0.59356	70.46269472
12	2306	2314	-0.17645	6.2269205
12	2306	2319	-0.29598	17.52083208
13	2311	2314	-0.58484	68.40756512
13	2312	2314	0.34906	24.36857672
14	2305	2317	-0.14125	3.9903125
14	2305	2318	0.11592	2.68748928
14	2308	2317	-0.19403	7.52952818
14	2308	2318	-0.13944	3.88870272
14	2311	2314	0.12458	3.10403528
14	2311	2317	0.26515	14.0609045
14	2311	2318	0.28032	15.71586048
14	2311	2322	-0.12162	2.95828488
14	2312	2317	-0.28851	16.64760402
14	2312	2318	0.32555	21.1965605
15	2305	2317	-0.17691	6.25942962
15	2305	2318	0.18433	6.79550978
15	2308	2317	0.10608	2.25059328
15	2308	2318	0.30644	18.78109472
15	2311	2317	-0.286	16.3592
15	2311	2318	-0.2129	9.065282
15	2312	2317	-0.28349	16.07331602
15	2312	2318	0.25594	13.10105672
16	2308	2314	-0.13578	3.68724168
16	2311	2314	0.35927	25.81498658
16	2312	2314	0.58235	67.8263045

Table S9 c) Cofactor % Contributions* to Molecular Orbitals for Model 4 (23 amino acids, full phytol chains) CAM-B3LYP /6-31G(d, p)

	Phe D1	Phe D2	P D1	P D2	Chl D1	Chl D2
2301	0.0	0.3	0.0	0.2	0.0	99.0
2302	97.1	0.0	0.7	0.0	1.8	0.0
2303	2.4	0.0	0.2	0.3	96.2	0.0
2304	0.0	96.0	0.0	0.3	0.0	3.4
2305	0.0	0.2	58.3	39.3	0.7	0.5
2306	0.0	3.3	0.5	0.4	0.0	95.5
2307	97.3	0.0	0.8	0.6	0.9	0.0

2308	0.9	0.0	39.1	58.5	0.3	0.1
2309	0.4	0.0	0.4	0.9	98.0	0.0
2310	0.0	99.1	0.0	0.3	0.0	0.1
2311	0.0	0.0	12.1	86.6	0.5	0.0
2312	0.0	0.0	88.0	10.9	0.1	0.1
2313	98.3	0.0	0.4	0.0	0.7	0.0
2314	0.0	0.8	0.0	0.2	0.0	98.6
2315	0.8	0.0	0.5	0.4	97.8	0.0
2316	0.0	98.7	0.0	0.2	0.0	0.7
2317	0.0	0.0	49.6	49.0	0.3	0.1
2318	0.0	0.0	48.7	50.1	0.1	0.1
2319	0.0	0.5	0.1	0.4	0.0	98.8
2320	98.4	0.0	0.3	0.0	0.8	0.0
2321	0.8	0.0	15.3	0.7	82.3	0.0
2322	0.1	0.0	73.9	9.7	15.1	0.1
2323	0.0	98.9	0.0	0.6	0.0	0.2
2324	0.0	0.0	10.6	87.6	0.6	0.1
2325	98.0	0.0	0.2	0.0	0.2	0.0

* as determined by the Mulliken Charges method in MultiWfn
